# Multiscale Model of Antiviral Timing, Potency, and Heterogeneity Effects on an Epithelial Tissue Patch Infected by SARS-CoV-2

**DOI:** 10.3390/v14030605

**Published:** 2022-03-14

**Authors:** Juliano Ferrari Gianlupi, Tarunendu Mapder, T. J. Sego, James P. Sluka, Sara K. Quinney, Morgan Craig, Robert E. Stratford, James A. Glazier

**Affiliations:** 1 Department of Intelligent Systems Engineering and Biocomplexity Institute, Indiana University, 2425 N Milo B Sampson Ln, Bloomington, IN 47408, USA; tjsego@iu.edu (T.J.S.); jsluka@indiana.edu (J.P.S.); glazier@indiana.edu (J.A.G.); 2 Division of Clinical Pharmacology, Department of Medicine, Indiana University School of Medicine, 950 W Walnut Street, Indianapolis, IN 46202, USA; squinney@iupui.edu (S.K.Q.); robstrat@iu.edu (R.E.S.J.); 3 Sainte-Justine University Hospital Research Centre and Department of Mathematics and Statistics, Université de Montréal, Montreal, QC H3T 1J4, Canada; morgan.craig@umontreal.ca

**Keywords:** SARS-CoV-2, antiviral therapy, remdesivir, mPBPK, virtual tissue simulation, agent-based model, multiscale model, tissue model

## Abstract

We extend our established agent-based multiscale computational model of infection of lung tissue by SARS-CoV-2 to include pharmacokinetic and pharmacodynamic models of remdesivir. We model remdesivir treatment for COVID-19; however, our methods are general to other viral infections and antiviral therapies. We investigate the effects of drug potency, drug dosing frequency, treatment initiation delay, antiviral half-life, and variability in cellular uptake and metabolism of remdesivir and its active metabolite on treatment outcomes in a simulated patch of infected epithelial tissue. Non-spatial deterministic population models which treat all cells of a given class as identical can clarify how treatment dosage and timing influence treatment efficacy. However, they do not reveal how cell-to-cell variability affects treatment outcomes. Our simulations suggest that for a given treatment regime, including cell-to-cell variation in drug uptake, permeability and metabolism increase the likelihood of uncontrolled infection as the cells with the lowest internal levels of antiviral act as super-spreaders within the tissue. The model predicts substantial variability in infection outcomes between similar tissue patches for different treatment options. In models with cellular metabolic variability, antiviral doses have to be increased significantly (>50% depending on simulation parameters) to achieve the same treatment results as with the homogeneous cellular metabolism.

## 1. Introduction

The COVID-19 pandemic has inspired the rapid discovery, development, and distribution of antiviral and immune-modulatory drugs and vaccines in the last two years. Computer simulations of within-host response have assisted in the rapid screening of candidate drug treatments [1]. Mathematical models and their computer simulations enable us to explore alternative treatment regimens using existing drugs rapidly [2]. Models of absorption, distribution, metabolism, and elimination (*ADME*) in specific organs and the body as a whole and the pharmacokinetics of drugs within individual cells, such as at the cellular infection level, and the immune system, can be leveraged to advise clinical trials for infectious diseases [3,4,5].

Several clinical trials of remdesivir as a possible treatment for COVID-19 followed the declaration of the 2020 pandemic by the World Health Organization [6,7,8]. Remdesivir is a single diastereomeric mono-phosphoramidate prodrug designed to arrest the replication of RNA viruses. Upon remdesivir administration, the patient’s body generates sequential metabolic intermediates before forming the active nucleoside triphosphate, GS-443902 (GS-441524-triphosphate). The active metabolite then binds to the elongating viral RNA synthesized by RNA-dependent RNA polymerase (*RdRp*) as a nucleoside analog and blocks viral replication [9]. The first clinical trials for remdesivir were as a treatment for the Ebola virus [8,10]. Most of these trials administered a 200 mg intravenous (*IV*) infusion loading dose followed by 100 mg IV daily infusions for five to ten days. However, the full breadth of therapeutic schedules remains unexplored, given the urgency required for drug development during the pandemic. To that end, we modelled remdesivir and its mechanism of action (*MOA*) on a patch of lung epithelial tissue infected by SARS-CoV-2 to provide a more comprehensive understanding of the interplay of remdisivir dose and timing, and outcomes.

Even though we frame our work in the context of SARS-CoV-2 and remdesivir treatment, our methods are general to other viral infections and antiviral treatments. We have developed our own MOA model for remdesivir. There are several antiviral drugs with similar MOAs [11,12], and previous modeling works simulated treatment with these drugs [13,14]. Experiments on SARS-CoV-2 infection in non-human primates (rhesus macaques) and associated mathematical models have shown that an antiviral drug treatment with lower efficacy may elongate the duration of the viremic profile even if the treatment initiation is very early [15,16,17]. Given these results, the present model focuses on the relations of the drug potency, treatment initiation time and dose interval with the viraemia as crucial players, and performs a thorough scan of all related parameters to elucidate a reasonable treatment regimen.

Various models have described remdesivir’s pharmacokinetics (PK), models ranging from one-compartment models to complex physiologically-based pharmacokinetic (*PBPK*) models [6,7,18]. Studies have also developed combined pharmacokinetic–pharmacodynamic (*PK-PD*) models for COVID-19. Goyal et al. used a two-compartment PK model for remdesivir at different potency and timing of treatment to predict how other parameters affect disease progression and treatment efficacy [19,20]. The authors observed that initiating antiviral treatment after symptom onset required antiviral concentrations that reduced viral production rate by more than 90% (>90% drug efficacy) to achieve a two log reduction in plasma viral load. If administration started at the time of infection (before the onset of symptoms), 60% drug efficacy achieved a similar reduction of viral load. They have also run theoretical kinetics of remdesivir drug resistance for various treatment regimens.

For the pharmacokinetics of remdesivir, we modified a PBPK model created by Gallo [18], a hybrid, full-PBPK model for remdesivir with 15 tissue compartments. Their PBPK model is very detailed and recovers remdesivir’s dynamics in several tissues and plasma. Our focus is on the concentration of the active metabolite of remdesivir in lung epithelial cells; therefore, we opted to simplify Gallo’s model (see Section 2.1). As remdesivir is given intravenously (IV) and has a long half-life ($t_{1/2}$ = 30.2 h) [7], we study dosing intervals longer than one day. Treatments using more extended periods may be helpful for patients that require remdesivir administration but are not in a condition severe enough that requires hospitalization. This approach could help alleviate hospital overcrowding and could improve treatment adherence. We also aim to characterize the interplay of drug potency and schedule on infection dynamics and treatment outcomes.

The above models (coupled population, PK and PD models) can be used to study effects on infection dynamics arising from changing drug potency, half-life, and dosing schedule. Population models assume well-mixed conditions, meaning that the model exposes the entire cell population to the same amount of infectious virus at any instance. A cellular agent-based model (*ABM*) can complement such models by adding multicellular-scale resolution [21]. ABMs are an effective simulation technique to model a population of agents. In ABMs, the agents are capable of independent decision making according to assigned attributes and conditions [22,23]. Cellular ABMs can introduce tissue heterogeneity to models by their very nature, as cells are individually modeled and can differ from one another, space itself is a model component [24,25,26,27,28]. A recent report on comparative biology immune ABM (*CBIABM*) has presented a model of mechanism-based differences in bat and human immune systems and discusses the consequences of these differences on disease manifestation [29]. In [25], population models of infection calibrated to experimental data were used to generate an equivalent spatially heterogeneous *ABM* of infection. The authors found that viral infectivity estimates using the ABM differed from the estimates from the population model by as much as 95% [25]. These differences in viral infectivity, or some other characteristic of the infection dynamics, could mean that a population model and an ABM calibrated to the same experimental data can significantly differ in their estimates for effective drug doses and schedules.

Furthermore, infection in a tissue starts from some discrete points of infection and spreads from them [30,31]. Therefore, spatially heterogeneous distribution of target cell states is expected, with further disease progression near the initial infection location (necrotic sites), to regions farther from initial infection sites, where the infection has not begun. Cytokines concentrations will also be heterogeneous. Since infection can spread within a tissue even if a few cells release virus, this spatial relationship between uninfected and virus releasing cells may determine how effective an antiviral needs to be to contain the viral spread. Heterogeneity in cells reactions and drug delivery and its possible effects on disease and treatment are topics of active study for COVID-19 [32], other diseases, and substance toxicity [33,34,35,36].

In the present ABM, we leverage our already established model of epithelial lung tissue infected by SARS-CoV-2 [24] implemented in CompuCell3D [37]. Our simulated environment models epithelial lung tissue infected by SARS-CoV-2, including cell surface-receptor (*ACE2*) affinity, intra-cellular viral replication, infectious-diffusive virus release, immune response, cytokine signaling by the epithelial and immune cells. We expand on those capabilities by incorporating a pharmacokinetic (PK) model of remdesivir and its dosing regimens, as well as a model for remdesivir’s mode of action (*MOA*). We explore the effect of varying the time of treatment initiation (from the number of hours after the infection of ten epithelial cells), the potency of remdesivir’s active metabolite (by varying *IC*_50_), and the interval between doses.

As we are simulating a spatially resolved model, we can test the effects of cell-to-cell variability. The amount of drug reaching each cell in the target tissue varies. This variation can result from: (1) different availability and different distance from capillaries (microdosimetry); (2) uptake rate differences (density and dynamics of cell-surface proteins); (3) conversion rate from prodrug to active metabolite based on intra-cellular enzyme concentrations; (4) effect of cell ageing on metabolic rates; (5) cell-cycle phases. To model each of these separately, one needs a detailed model of cellular metabolism, life cycle and capillary structure. Therefore, in the simulations, we expose each cell to a homogeneous concentration of the antiviral drug, and combine the different possible sources of intra-cellular metabolic heterogeneity into an effective change of the uptake and elimination rates; see Section 2.2 and Section 2.5.

Cells with internal concentrations of remdesivir-active metabolite below concentrations that control the viral replication are significant contributors to viral synthesis and release, and determine the consequent spread of infection. Their spatial distribution in the tissue is key, as those will be the regions of significant infection activity. The duration over which the concentration of the active metabolite is below the effective concentration also matters. Our previous work [24] demonstrated that if cells unblock RNA synthesis, even for a short time, the amount of functional RNA produced will be small, and one can expect reasonable inhibition of viral release.

We believe our methods can be of great use in early drug and treatment development when characterization of the drug’s PK and PD are not well established. We change remdesivir’s potency and half-life in our model to investigate how those changes affect the disease progression and treatment effectiveness (see Section 3.3.2 and Section 3.3.3). Our heterogeneous drug metabolism model predicts that higher doses (by ≈50% or more) are necessary to achieve the same level of treatment success compared to our homogeneous metabolism model results (see Section 3.3.2 and Section 3.3.4). Although the cellular-level heterogeneity has not been measured experimentally, our results suggest that treatment outcomes depend on the intensity of heterogeneity (see Section 3.3.6). We hypothesize that the least sensitive cells to the antiviral drive the infection forwards (super-spreader cells).

This work addresses the following questions: How significant are the effects of remdesivir’s dosing interval on treatment outcomes? What is the impact of heterogeneous cellular drug uptake and elimination on viral load (heterogeneous cellular drug metabolism)?

## 2. Materials and Methods

We sought to compare the spread of infection as represented by the total number of cells infected versus time and the viral load versus time under different remdesivir treatment schedules. Achieving this goal requires several model components: (1) viral entry into the target epithelial cells, (2) viral replication within epithelial cells, (3) release of virus and spread of infection within a tissue, (4) immune response and its effect on viral spread within the tissue, (5) the kinetics of the concentration of drug active metabolite as a function of time after dosing and (6) effect of the active metabolite on viral replication within individual infected epithelial cells.

Our computational model includes a multicellular spatial model of SARS-CoV-2 infection of a lung tissue patch composed of epithelial cells, an effective immune response module, and a minimal PK model of available active metabolite of remdesivir in individual epithelial cells. The PK model estimates changes in concentrations of remdesivir triphosphate (GS-443902) in lung epithelial cells after intravenous (IV) infusion of remdesivir in humans. We base our PK model on the Gallo model [18]; Gallo’s model estimates GS-443902 kinetics in peripheral blood mononuclear cells (PBMC) following IV infusion of remdesivir. We assume that the kinetics of exposure, uptake, and metabolism of the two cell types (PBMC and lung epithelial cells) are similar, although the absolute tissue metabolite concentrations might differ. We describe each sub-model and their integration below.

### 2.1. Remdesivir Physiologically Based Pharmacokinetic Model

**Figure 1:**
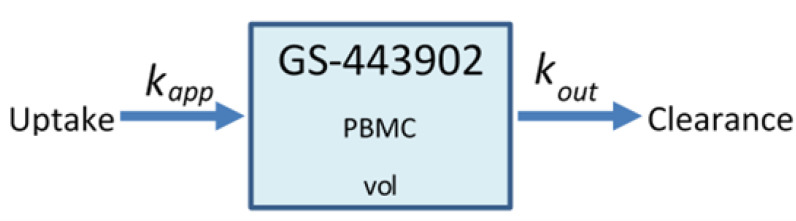
Schematic diagram of the minimalized PBPK model of remdesivir. PBMCs are a surrogate for lung alveolar epithelial cells for GS-443902.

**Figure A1:**
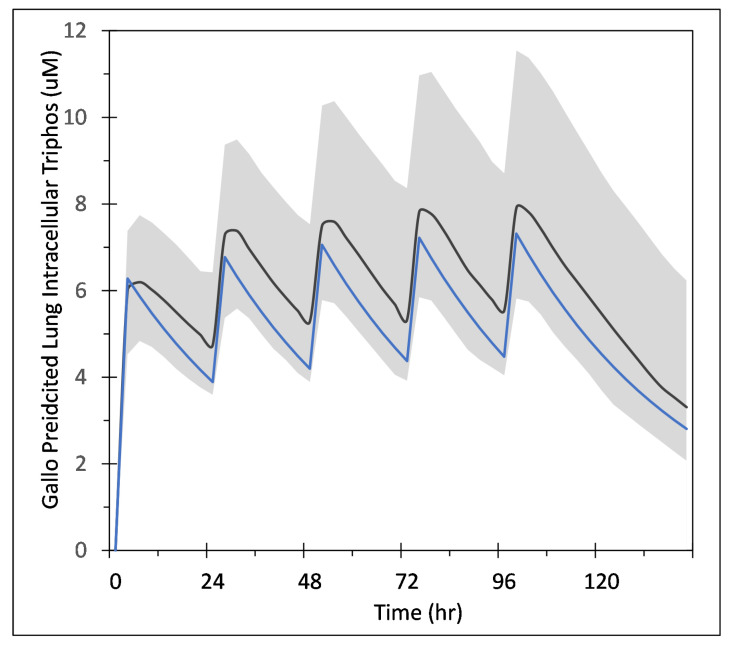
Comparison of our simplified model to Gallo’s population simulation plot of predicted intracellular lung GS-443902 concentration. Gallo’s mean response is shown by the black line and the 95% interval is shaded. This data is from 5000 simulations in which the two key parameters in the Gallo model were sampled from distributions with 20% CV. See Gallo and their supplement Figure S5 for more information. Blue line is our simplified model’s output. The Gallo data used in this plot was digitized from the publication using https://automeris.io/WebPlotDigitizer/ (accessed on 3 November 2021).

Gallo [18] previously published a detailed PBPK model of remdesivir. As we focus on the concentration of the active metabolite of remdesivir in lung epithelial cells, many details of the Gallo model are of limited relevance to our work. Accordingly, we build a parsimonious model to replicate the ADME and intra-cellular availability of remdesivir’s active metabolite based on the time course following IV infusion of remdesivir. We use COPASI [38] version 4.30 (Build 240) to replicate Gallo’s model and build our simplified model. The original PBPK model included 6 differential equations and 12 parameters. Our model assumes that remdesivir is metabolized into its active component (GS-443902) in the target tissue at the infusion rate, as its metabolism into the active metabolite is very rapid in the target tissue [10], then GS-443902 is eliminated by a first-order process. Figure 1 shows the simplified model structure. The single remaining equation for the PK model representing GS-443902 as a function of the infusion rate of remdesivir is:

(1a) $$\frac{d\left(C_{GS}\right)}{dt}=k_{app}D_{rmd}-k_{out}C_{GS}\;\;,$$

(1b) $$k_{app}=\frac{k_{in}}{vol\;\;\;\tau_{I}}\;\;,$$

with $k_{in}$ denoting the uptake rate of GS-443902, $C_{GS}$ the concentration of GS-443902, $D_{rmd}$ the dose of remdesivir, vol the effective compartment volume (in Liters), $\tau_{I}$ the time required to infuse remdesivir (1 h), and $k_{out}$ the excretion rate for the active metabolite. We use $k_{in}$ as the infusion on/off switch. The values for the parameters are in Table 1. As part of this study, we investigate the effects of schedule on the simulated treatment, e.g., remdesivir is given every 48 h instead of every 24. To keep the total amount of remdesivir administered constant, we change the infusion amount $D_{rmd}$ with the schedule change, e.g., the 48 h schedule uses a dose of remdesivir that is double that of the 24 h schedule, $D_{rmd}$(48 h) = $2\times D_{rmd}$(24 h). As shown in Appendix A Figure A1, this simple model reproduces the $C_{GS}$ time course within the uncertainty in the Gallo model, including underlying PBMC data as well as the European Union’s compassionate use data [39].

### 2.2. Sego et al.’s Agent-Based Model

To model the spread of viral infection, cytokine response, and immune cell activity in a tissue, we use the established hybrid multiscale agent-based model of SARS-CoV-2 infection in a small lung tissue patch from Sego et al. [24]. In this model, the infection progresses as follows. The initially infected cell(s) goes through the viral life cycle. The infected cell(s) could die during the eclipse phase, but if it survives, after the eclipse phase, it releases virus into the extracellular space and continues to release virus until it dies. The virus diffuses and decays extracellularly according to Fick’s law, (for the full diffusion equations, please see Equations (20)–(23) in [24]), and reaches neighboring cells, which in turn have a probability of internalizing virus from the extracellular space and becoming infected (eclipse phase). At early times, the only virus available to infect additional cells is that which is released by the initially infected cell(s). Early in the infection, this mechanism of cyclic infection can lead to quasi-synchronous bursts of cells becoming infected. The description of Sego et al.’s model is extensive [24]. We summarize here the parts relevant to our work; for a full description, please see [24]. We note that with the default parameters used by Sego et al. [24] all the simulated epithelial cells are eventually infected and die. However, depending on the parameters used, they also saw containment of the infection by the immune system [24].

In more detail, Sego et al.’s [24] agent-based model is a 2.5-dimensional Cellular Potts model (*CPM*), and is implemented in CompuCell3D [37]. We use CompuCell3D version 4.2.3 to generate the results in this paper. The 2.5 dimensions of the model consist of a 2D epithelial sheet with 900 non-motile epithelial cells and a 2D interstitial plane where immune cells are represented, move, and interact with the environment. Diffusion-decay of cytokines and extracellular virus occurs in this interstitial plane. Together, the planes represent a patch of (epithelial) lung tissue of size 0.9 mm × 0.9 mm, represented by 90 × 90 × 2 pixels. We keep several simplifications made in the original study, e.g., the model does not include tissue recovery or creation and release of antibodies [24]. Sego et al. started the simulation with one infected cell in the patch (out of 900 cells), and they ended their simulations at day 14. We opt to simulate for 28 days even though the biological realism declines (e.g., lack of tissue recovery or antibody response). Each of our ABM simulations over 28 days took 40 to 100 min of computation time on Indiana University’s Carbonate super-computer. The parameter values used by Sego et al. are tabulated in our Appendix B Table A2, Table A3 and Table A4, and in the original paper [24].

The model by Sego et al. [24] includes only a single immune cell type that aggregates behaviors exhibited by different immune cells, mainly NK cells and CD8+ T cells. The immune cells chemotax up the local cytokine gradient, kill infected cells on contact, and release a cytotoxic agent depending on the severity of the infection. After exposure to local cytokine, immune cells start to release cytokine, taking part in the signaling. As the modeled immune cells present behaviors of NK- and T cells, the adaptive immune system is partially represented. In Sego’s model, only one cytokine is used to represent all the cytokines involved in immune signaling. The cytokine level and the number of immune cells in the infection zone define the system’s overall pro- or anti-inflammatory state of the system. If the system is in the pro-inflammatory state, there is a stochastic chance of seeding new immune cells in the domain. If the immune system model is in the anti-inflammatory state, the simulation may remove immune cells through a stochastic process.

In their paper, Sego et al. [24] characterized possible end states for the simulated tissue domain. By varying the infectivity of the virus and the strength of the immune response, they can simulate situations where the infection sweeps the tissue, cases where disease containment is achieved, and situations in which the infection is eliminated but reoccurs (recurring infection of SARS-CoV-2 has been observed in vitro [40]). They also characterized a particular case of note, “*failure to infect*,” a situation where the infection does not spread significantly beyond the initially infected cells. This situation happens because of the stochasticity of the simulation, e.g.,  if an immune cell is seeded near the only infected cell and kills it, or if the infected cell dies before releasing infectious virus to the environment.

As we are simulating an intervention that aims to contain the spread of infection, differentiating spontaneous results of “*failure to infect*” and effective treatment would be difficult and cumbersome. We, therefore, opted to perform more simulation replicas with the default parameters of Sego et al.’s [24] model but varying the initial number of infected cells. We ran Sego et al.’s [24] model with a single infected cell at the start out of 900 epithelial cells (same as in the original), with two infected cells out of 900, five out of 900, and ten out of 900. For each of those initial conditions, we ran 400 simulation replicas.

As mentioned, in the original model [24], the single initially infected cell (out of 900 epithelial cells) is placed in the center of the simulated domain; we randomize the position(s) of our initially infected cell(s). We can quantify the “*failure to infect*” rate and choose a starting condition that fits the goals of this paper. Results of this investigation are in Section 3.2 . We find that starting with five initially infected cells eliminates spontaneous failures to infect without resulting in a fast sweep of the tissue by the infection (mean time to full tissue infection stays close to 15 days).

### 2.3. Viral Life Cycle Model

The viral life cycle model begins with virus that is diffusing in the extracellular matrix entering into the cells [24]. For each cell (σ), the uptake of discrete viral particles is modeled as a stochastic process that depends on (1) the amount of virus on the cell surface ($c_{vir}\left(\sigma \right)$), (2) the cell’s volume (ν(σ)), (3) the number of unbound ACE2 receptors the cell has (SR(σ)), (4) the cell’s initial number of unbound receptors ($R_{o}$), (5) the binding affinity ($k_{on}$) of the virus with the available unbound cell receptors, (6) the virus–receptor dissociation affinity ($k_{off}$), and (7) the time for a single uptake event to occur ($\alpha_{upt}$). During a short time interval $\Delta t<<\alpha_{upt}$, the probability of viral uptake, the amount of virus internalized (if uptake occurs), and the change of surface receptors (if uptake occurs) from [24] are, respectively,

(2a) $$Pr\left(Uptake\left(\sigma \right)>0\right)=\frac{\Delta t}{\alpha_{upt}}\frac{{\left(c_{vir}\right)}^{k_{upt}}\left(\sigma \right)}{{\left(c_{vir}\right)}^{k_{upt}}\left(\sigma \right)+{\left(V_{upt}\right)}^{k_{upt}}}\;\;,$$

(2b) $$V_{upt}=\frac{R_{o}}{2k_{on}\nu \left(\sigma \right)SR\left(\sigma \right)}\;\;,$$

(3) $$Uptake\left(\sigma \right)=\frac{1}{\Delta t}Pr\left(Uptake\left(\sigma \right)>0\right)c_{vir}\left(\sigma \right)\;\;,$$

(4) $$\frac{dSR(\sigma )}{dt}=-Uptake\left(\sigma \right)\;\;.$$

Here, $V_{upt}$ is the Michaelis constant of the viral concentration for which the probability of uptake is half maximum and $k_{upt}$ is the Hill coefficient. After cellular uptake, the amount of virus that enters the cell is removed from the viral field over the cell domain.

A tri-phasic ordinary differential equation (*ODE*) system governs intra-cellular viral reproduction in each cell (σ) [24]. The first one is the eclipse phase (no viral release). After this phase, cells start to release virus at an increasing (but saturating) rate until the cell dies. The viral replication ODE consists of four processes and variables representing different parts of the viral replication process: unpacking virions in the cell (Equation (5)), genome replication (Equation (6)), protein synthesis (Equation (7)), repackaging of new virions (Equation (8)). Those equations, from [24], are:

(5) $$\frac{dU}{dt}\left(\sigma \right)=Uptake\left(\sigma \right)-r_{u}U\left(\sigma \right)\;\;,$$

(6) $$\frac{dR}{dt}\left(\sigma \right)=r_{u}U\left(\sigma \right)+r_{max}R\left(\sigma \right)\frac{r_{half}}{R\left(\sigma \right)+r_{half}}-r_{t}R\left(\sigma \right)\;\;,$$

(7) $$\frac{dP}{dt}\left(\sigma \right)=r_{t}R\left(\sigma \right)-r_{p}P\left(\sigma \right)\;\;,$$

(8) $$\frac{dA}{dt}\left(\sigma \right)=r_{p}P\left(\sigma \right)-Release\left(\sigma \right)\;\;,$$

(9) $$Release\left(\sigma \right)=r_{s}A\left(\sigma \right)\;\;,$$

with *Uptake* the result from Equation (3), $r_{u}$ rate of unpacking the internalized virus, $r_{max}$ the maximum viral replication rate, $r_{t}$ the rate of translation of viral genome into RNA templates for protein synthesis, $r_{p}$ protein packing rate, $r_{s}$ assembled virus release rate. The values for the parameters in Equations (2)–(9) are in Sego’s Table 1 [24] and in our Appendix B. In this study, we extend the viral life cycle model to include the effects of the antiviral modeled in Section 2.1.

### 2.4. Remdesivir Mode of Action (MOA) Model

Remdesivir works as a nucleotide analog by binding to the elongating RNA being synthesized by RdRP [9]. To model this, we extend Sego’s [24] genome replication Equation (6) by modifying $r_{max}$ as a function of the cell (σ) intra-cellular GS-443902 concentration ($C_{GS}\left(\sigma \right)$). Thus, we modify Equation (6) to

(10) $$\frac{dR}{dt}\left(\sigma \right)=r_{u}U\left(\sigma \right)+r_{max}^{\prime }\left(\sigma \right)R\left(\sigma \right)\frac{r_{half}}{R\left(\sigma \right)+r_{half}}-r_{t}R\left(\sigma \right)\;\;.$$

We also add the following equation for $r_{max}^{\prime }$,

(11) $$r_{max}^{\prime }\left(\sigma \right)=r_{max}\;\;\frac{IC_{50}^{2}}{IC_{50}^{2}+C_{GS}^{2}\left(\sigma \right)}=r_{max}\;\;\frac{1}{1+{\left(\frac{C_{GS}\left(\sigma \right)}{IC_{50}}\right)}^{2}}\;\;,$$

where $IC_{50}$ refers to GS-443902’s intra-cellular $IC_{50}$, the value for $IC_{50}$ is in Table 2.

The equation for $r_{max}^{\prime }$ is a inhibitory Hill function. In order to determine the Hill coefficient, we fit a Hill equation to data of SARS-CoV-2 inhibition by remdesivir from Choy et al. [41] and Pizzorno et al. [42]. We found the value of the Hill coefficient in the range from 1 to 4 and chose 2 as the value to be used in the current study. As the intra-cellular $IC_{50}$ for GS-443902 is unknown, especially in vivo [43,44], we define a base $IC_{50}$ of 7.897 μmol/L using our pharmacokinetics model (see Section 2.1 and Section 3.1, and Table 1). Our investigation explores different drug potencies or doses by varying this base $IC_{50}$ by using multipliers in the range [0.01,10] (Table 2).

### 2.5. Heterogeneous Cellular Metabolism of Remdesivir Modeling

The simple remdesivir PK model is present in our multicellular simulations as a component of lung epithelial cells. Each epithelial cell has a synchronized but independent copy of the model, and each cell occupies a different region of space. This method allows us to investigate the effects of heterogeneous metabolism of remdesivir by the epithelial cells. The present model avoids any complex tissue specifications by simulating a tiny patch of lung epithelial tissue. The model focuses how the intercellular heterogeneity of drug metabolism is concerned with the infection outcome. Certainly, there are many other sources of heterogeneity, which we do not consider here to minimize the cross-correlation of the stochasticity from multiple sources of heterogeneity, e.g., distance from blood capillaries, tissue topology, and cell age.

We initialize each cell with drug metabolic parameters ($k_{in}$ and $k_{out}$) at the beginning of the simulation. At the same time, to simulate metabolic heterogeneity, we modulate the metabolic parameters with random numbers selected from a Gaussian distribution. For a cell ($\sigma_{A}$), we draw a random number $\theta (\sigma_{A})$ for each of those metabolic parameters, and we then change each metabolic parameter by multiplying it by $1+\theta (\sigma_{A})$, i.e.,

(12) $$\theta_{in}\left(\sigma_{A}\right)=N\left(\mu =0,\xi =0.25\right)\;\;,$$

(13) $$k_{in}^{\prime }\left(\sigma_{A}\right)=k_{in}\;\;\left(1+\theta_{in}\left(\sigma_{A}\right)\right)\;\;,$$

(14) $$\theta_{out}\left(\sigma_{A}\right)=N\left(\mu =0,\xi =0.25\right)\;\;,$$

(15) $$k_{out}^{\prime }\left(\sigma_{A}\right)=k_{out}\;\;\left(1+\theta_{out}\left(\sigma_{A}\right)\right)\;\;.$$

θ is selected from the normal distribution N with mean μ (set to 0) and standard deviation ξ (set to 0.25). If |θ|>1, we draw a new number from N until |θ|≤1. To keep the total metabolic rate over the simulated tissue patch constant, we select another cell ($\sigma_{B}$) to have its metabolic rates modified by $1-\theta (\sigma_{A})$ (the same θ as for cell $\sigma_{A}$),

(16) $$k_{in}^{\prime }\left(\sigma_{B}\right)=k_{in}\;\;\left(1-\theta_{in}\left(\sigma_{A}\right)\right)\;\;,$$

(17) $$k_{out}^{\prime }\left(\sigma_{B}\right)=k_{out}\;\;\left(1-\theta_{out}\left(\sigma_{A}\right)\right)\;\;.$$

**Figure 2:**
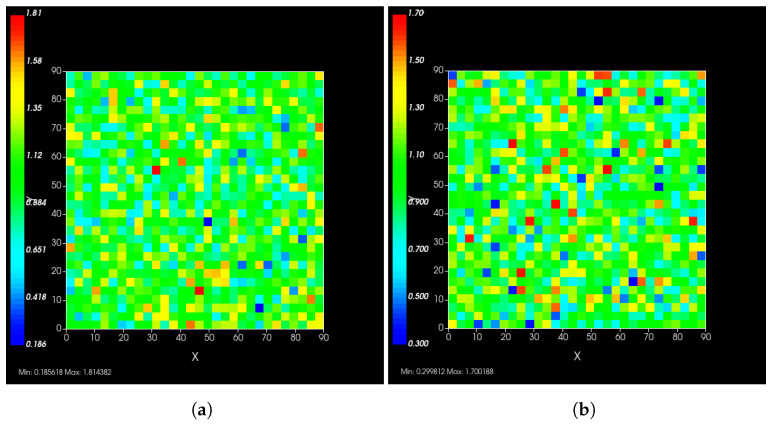
Simulated epithelial cell layers colored by the change in $k_{in}$ (**a**), $k_{out}$ (**b**). The values displayed are relative to the base $k_{in}$ and $k_{out}$, a cell colored blue in (**b**) has, e.g., $k_{out}^{\prime }\left(\sigma \right)=0.3\times k_{out}$.

A spatial distribution of the modified rate parameters is shown in Figure 2.

Typical PBPK models do not consider the effects of heterogeneous responses by the cells in the tissue, as each compartment is considered well mixed. In reality, a cell with rapid clearance of the drug may deplete the intra-cellular antiviral and restart viral production, release and infect neighboring cells. The difference between our model and traditional PBPK models is precisely that our model addresses this spatial heterogeneity issue.

### 2.6. Simulating Antiviral Treatment Regimens and Treatment Classification Metrics

We simulate a 28 day daily dose regimen for remdesivir, studying the infection dynamics over that time. We initiate remdesivir intervention at different time points with a loading dose followed by a maintenance dose according to our PK model. The dose is rescaled based on the time in-between doses, i.e., if the daily dose is 100 mg, the dose every two days is 200 mg.

We start the simulation with five infected cells, and when another five cells are infected, we consider the infection is onset. We initiate treatment 0, 0.5, 1, or 3 days after infection onset. The initial level of infection of a tissue patch can be compared with the infection of a cultured cell population. For in vivo infection experiments with woodchuck hepatitis virus (*WHV*), Lew et al. [45] found different infectivity and pathogenicity outcomes with different inocula of similar titer concentrations. Beginning with ≈0.5% (5 out of 900) of tissue cells infected is higher than what would be expected. However, we can imagine our infected tissue patch is surrounded by uninfected tissue that we do not simulate. For each parameter combination, we run eight simulation replicas. We classify simulation results using the median behavior of critical simulation metrics, e.g., the uninfected population. Thus, we can not only differentiate effective from ineffective treatments but also create more specific classifications:

*Rapid clearance* (effective treatment) results from more potent and frequent treatments. Elimination of extracellular virus is achieved in less than 14 days, and there was no subsequent release of the newly generated virus (green plots in Section 3.3.2’s figures, and Appendix F).

*Slow clearance* (effective treatment): simulations where extracellular virus slowly decreases (over more than 14 days). In some cases, new extracellular virus was produced when the antiviral concentration was low, generating an oscillation of the extracellular virus concentration around the decreasing trend. In some slow-clearance simulations, the extracellular virus cleared completely, then infection restarted once the antiviral levels dropped sufficiently (blue plots in Section 3.3.2’s figures and Appendix F).

*Partial containment* (partially effective treatment): simulations with a mid-low potency result in a stable level of extracellular virus. Because the treatment is fairly effective, it keeps the viral load very low and the rate of infection of new cells is also very low. Therefore, many target cells remain uninfected throughout the duration of the simulation. The low viral load leads to a low continuum rate of infection of new target cells roughly balancing the death of virus producing cells and the clearance of extracellular virus. In this scenario, the low-dose treatments delay the infection of all target cells and, thus, delay the onset of target cell-limited viral clearance. However, the treatment by itself is insufficient to eliminate the virus completely during the period simulated. Clearance of this low number of infected cells would likely be achieved by the adaptive immune response, which we do not model. Note that if we ran the simulation for long enough, we would achieve either target cell-limited clearance or elimination of the virus by the drug. We do not imply that drug treatment makes the infection worse. The rough equilibrium of infection of new cells and viral clearance implies that these simulations are on the transition line separating effective from ineffective treatment. For a longer time between doses, we observe extracellular virus levels oscillating around a steady value (black plots in Section 3.3.2’s figures and Appendix F).

*Widespread infection* (ineffective treatment): simulations resulting from sufficiently low potency or large periods in-between doses have the extracellular amount of virus increase during the treatment simulation. Treatment in these cases does not impede infection of all cells and subsequent death of the simulated epithelial tissue occurs. Some simulations have viable cells at the end of simulation with the amount of virus still increasing. Presumably, if run for a longer time, these simulations would also result in complete infection and death of the tissue. This category includes simulations with an observed decrease in extracellular virus towards the end of the simulation; this occurs after the death of all epithelial cells as virus production ceases (target cell-limited clearance) (red plots in Section 3.3.2’s figures and Appendix F).

To classify the simulation results among these classes, we first check the median over simulation replicas of the uninfected cell population at the end of the simulation. If they number less than ten or less than half the median uninfected population at the start of treatment, we classify the treatment as ineffective with widespread infection. We then look at the median viral load; if it goes below a threshold and does not rise above it, we classify the results as “effective treatment” with rapid clearance (if cleared in less than 14 days) or slow clearance (otherwise). If the median viral load rises back to levels above the threshold, we look at the median viral load peaks trend. If the peaks are trending to higher levels, we classify the simulation results as ineffective treatment with widespread infection. If the trend is near zero (stable), we classify the results as partial containment. If we observe decreasing trends, we classify the results as slow clearance. Greater detail regarding the definition of these metrics are in Appendix C.

## 3. Results

### 3.1. Remdesivir PK Model

We first calculate the intra-cellular metabolite concentration over time as a function of dose and dosing schedule. The critical information is the time to accumulate to $IC_{50}$ and how long the concentrations stay above and below values that effectively shut down viral replication.

The intra-cellular $IC_{50}$ of GS-443902 is unknown [43,44]. To define an $IC_{50}$ for GS-443902 in our simulations and to characterize the GS-443902 concentration time course and accumulation in our PK simulation, we first simulated our PK model to 14 days. We then analyzed the peaks and troughs of the concentration and set the peak-troughs midpoint to be our model’s $IC_{50}$ (7.897 μmol/L, Table 2).

**Figure 3:**
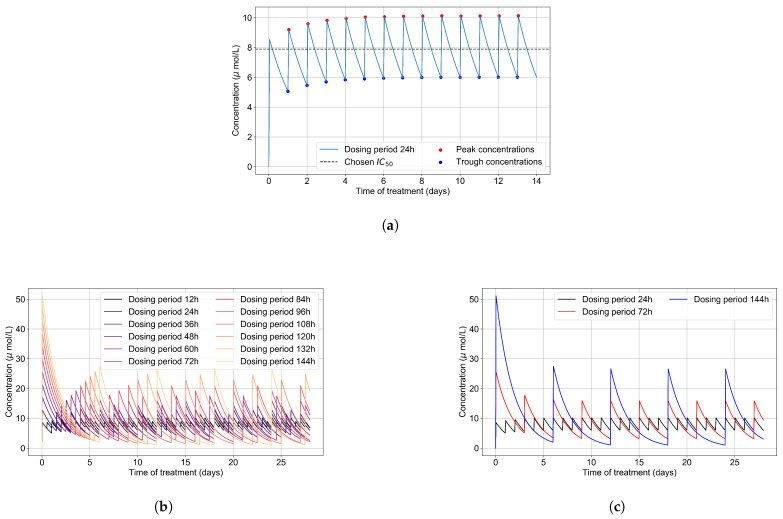
(**a**) Concentration of GS-443902 (remdesivir’s active metabolite) for a 14 day treatment with a 200 mg loading dose and 100 mg subsequent daily doses (IV infusion) is obtained by solving Equations (1a) and (1b). Concentration peaks (red) and troughs (blue) are pointed out, and their mid-point (dashed line) is our base $IC_{50}$. (**b**) Concentrations of the active metabolite, GS-443902, in PK simulations for the different dosing regimens of remdesivir, with the doses rescaled to keep the total average amount of remdesivir given over 24 h constant. (**c**) Some selected PK profiles from (**b**).

We then assessed the systems-level effects of different potencies of remdesivir. Clinically, drugs are usually given to achieve a plasma concentration of the drug of approximately 5–10-fold the $IC_{50}$ [46]. For the potency investigation, we multiply the base $IC_{50}$ by a set of multipliers (in the range of 0.01 to 10, Table 2). We explore dosing regimes in which remdesivir effectively shuts off viral production in all cells; other situations where it leaves a significant number of cells releasing virus at all times; and intermediate situations. Part of our investigation is about the effect of the dosing schedule. We have included the PK concentration profiles to 28 days for all schedules investigated in Figure 3b.

### 3.2. Variability of Outcomes in Sego’s Model

To understand the effect of treatment, first, we quantify the natural variability of infection progression outcomes in the original model (without any treatment simulation). This variability depends on model parameters, which we do not change from Sego et al. [24] and on the initial number of infected cells.

In Sego et al.’s model [24], as discussed previously in Section 2.2, simulations using the base parameters result in infection sweeping in the the tissue. However, some simulations using the default parameters show “*failure to infect*”, in which the initially-infected cells die before releasing enough virus to infect a substantial number of the remaining cells. This may happen because the initially infected cells are killed by immune system cells, or by viral stress, soon after the simulation starts, while it is in the eclipse phase. The simulation, therefore, ends with almost all cells uninfected. Sego et al. [24] also demonstrated that their model is capable of immune containment of the infection depending on the simulation parameters.

These “*failure to infect*” results are problematic when we start our treatment investigation. We cannot distinguish for any individual simulation replica in which the infection failed to spread, whether it failed because of the treatment or whether it would have failed to spread regardless of treatment. Distinguishing these cases from effective treatment would require a vast number of replica simulations. We significantly reduce the number of replicas required per parameter set by selecting initial conditions that guarantee widespread infection in the absence of treatment (i.e., eliminating “failures to infect”).

**Figure 4:**
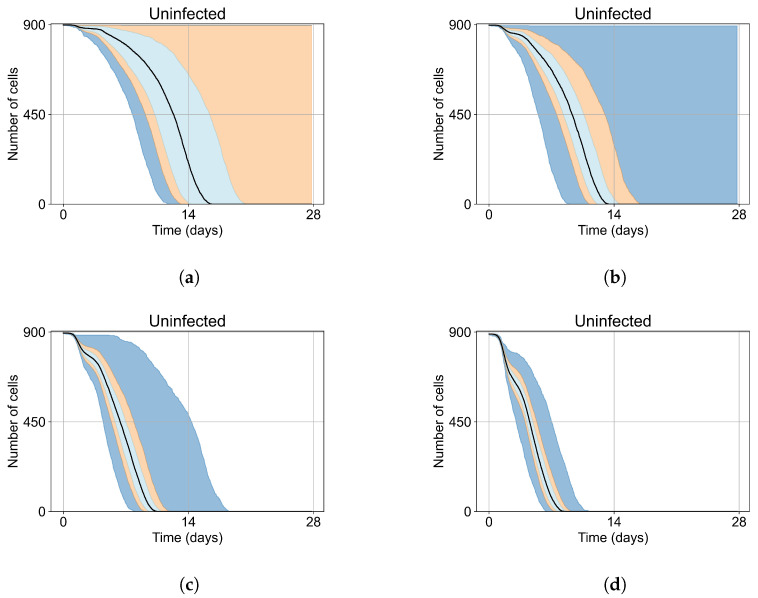
Uninfected cell populations for 400 replicas of Sego et al.’s model [24] performed by us are shown using Sego’s default parameters [24]. In all the cases, the medians of simulation replicas are in black lines, the 0th to 100th quantiles are shaded as dark blue, 10th to 90th shaded in orange, and the 25th to 75th as light blue. (**a**) Simulations start with 1 initially infected cell and 63 simulations result in “*failure to infect*” (15.75% of replicas), the 90th quantile includes the upper bound of the number of cells. (**b**) Simulations start with 2 initially infected cells where 5 simulations result in “*failure to infect*” (1.25%), and the 100th quantile includes the upper bound of the number of cells. (**c**) Simulations start with 5 initially infected cells. (**d**) Simulations start with 10 initially infected cells.

As mentioned, we run Sego et al.’s model [24] with a single infected cell at the start out of 900 epithelial cells, with two out of 900, five out of 900, and ten out of 900. To quantify the prevalence of “failures to infect,” we run 400 simulation replicates for each of those initial conditions. With a single initially infected cell, 15.75% of the replicas resulted in failures to infect; with two initially infected cells, there was a 1.25% rate of “*failure to infect*”; and with five and ten initially infected cells, there were no failures to infect. With a single initially infected cell, the mean time for widespread infection of the simulated patch was 16.04 days (excluding failures to infect), with two initially infected cells, the mean was 27.4 days (excluding failures to infect), with five 18.6 days, and with ten the mean time to full infection was 11.4 days (see Figure 4).

As the infection starts synchronized, different generations of infected cells were observed at early simulation times (Figure 4). This is observed as a sharp drop in uninfected cells followed by a less severe infection phase followed by another drop due to the first generations of infected cells releasing virus and dying while the next generation of infected cells is still in the eclipse phase or has not been infected yet. As the simulation progresses, the infection loses its synchronization due to the stochastic effects.

**Figure 5:**
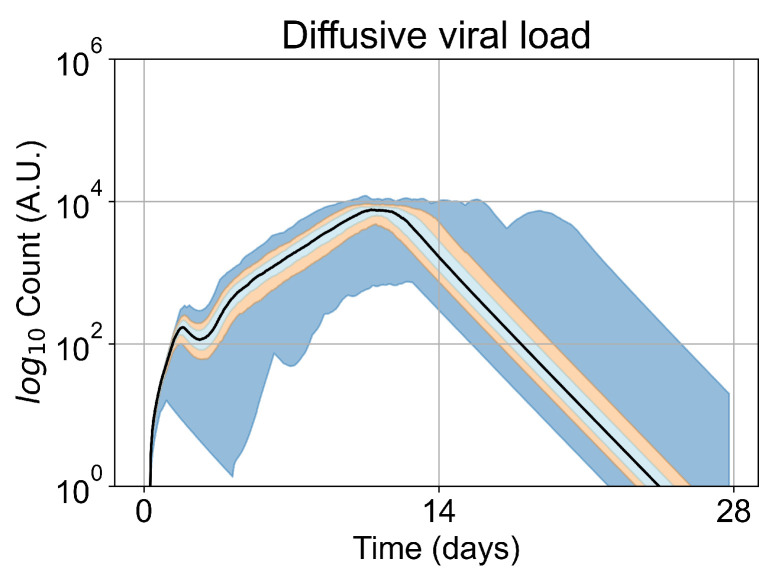
Extracellular viral load curve for untreated simulations with five initially infected cells in the tissue patch.

We opt to carry out the remainder of our investigations with five initially infected cells. Initiating simulations with five infected cells removes the failures to infect results (making treatment comparisons easier) while keeping the time to full infection similar to the original. In Figure 5, we show the viral load for simulations with five initially infected cells (i.e., the initial condition for our investigation) without any treatment. The viral load peaks at approximately day 10, which is within confidence intervals of measured viral load curves of within-host experimental data. For example, our viral replication model matches data of severe cases of COVID-19 in humans from Yanqun Wang, Lu Zhang, et al. [47]. In [47], patients with severe COVID-19 had a viral load peak 10–15 days after symptom onset. Those patients also had detectable virus after 20–25 days of symptom onset. In truth, viral load data for SARS-CoV-2 is still messy, especially with the several variants (of concern or not) in circulation (any of which could have different viral kinetics), as seen in Wölfel, Roman, et al. [48]. In any case, in this work we use SARS-CoV-2 and remdesivir as framing devices, not as the end goal of our simulations. The viral loads, viral production AUC and infected populations in untreated simulations with other initial conditions are in Appendix E.

### 3.3. Predictive Treatment Outcomes

We investigate the effects of frequency, potency, active metabolite half-life, treatment start time, and tissue heterogeneity on antiviral treatment outcomes for a viral infection on lung epithelial tissue. We model SARS-CoV-2 and remdesivir specifically, and our methods are generalizable to other viruses and antivirals. We first investigate the case of homogeneous tissue, beginning with a coarse variation of the $IC_{50}$ multiplier and dose-interval, (in Section 3.3.1). We then perform a finer-parameter investigation of those parameters to define the precise boundary of effective and ineffective treatment (see Section 3.3.2). We then investigate the effects of having a shorter half-life for the active component (see Section 3.3.3). Finally, we investigate the heterogeneous metabolism of GS-443902 by epithelial cells (see Section 3.3.4).

#### 3.3.1. Coarse-Parameter Variation

**Figure 6:**
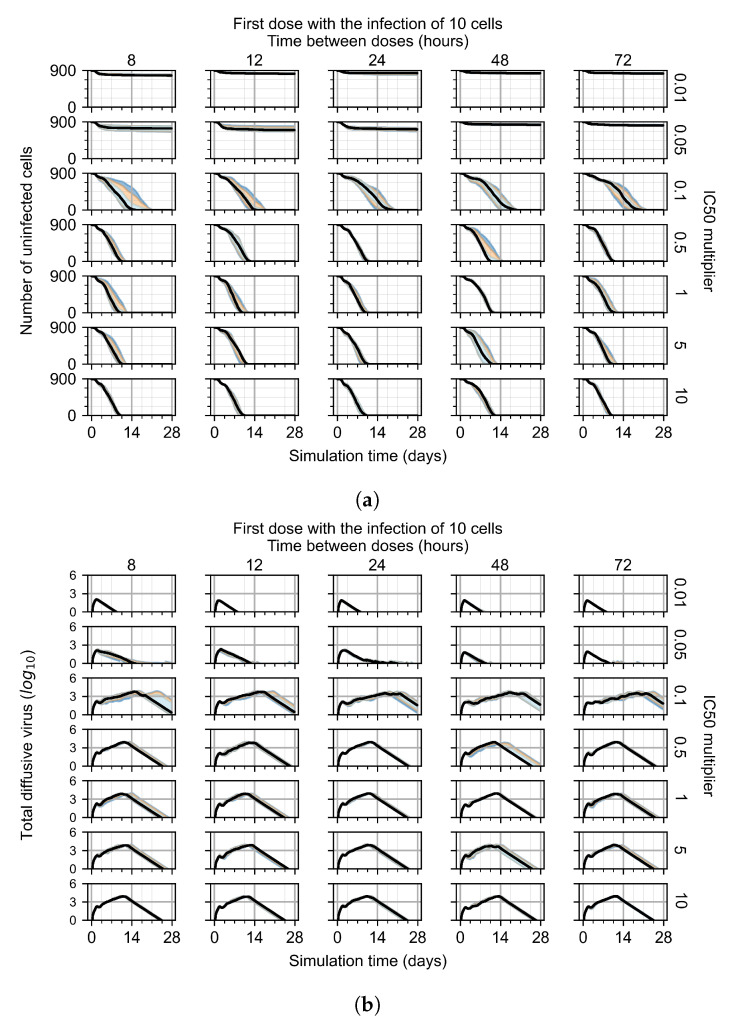
Coarse-parameter investigation (10 replicas of the treatment simulation). Treatment starts with 10 infected cells. For all subfigures, the median measurement of simulation replicas is the black line, the 0th to 100th quantiles are shaded as dark blue, 10th to 90th shaded in orange, and 25th to 75th as light blue. Treatments with an $IC_{50}$ multiplier <0.055 contain the infection, while treatments with $IC_{50}$ multiplier ≥0.05 do not. The top two rows show a reduction in viral load due to treatment, while in the lower two rows the decrease is due to all cells being dead. (**a**) Uninfected cell population. (**b**) Extracellular diffusive virus; *y*-axis in log scale, and exponent values as tick marks.

For the coarse-parameter investigation, we choose 0.01, 0.05, 0.1, 0.5, 1, 5, and 10 as the $IC_{50}$ multipliers and 8, 12, 24, 48, and 72 h as the dosing periods (Figure 6). At this point of the investigation, we do not explore the effect of delaying treatment initiation, all treatments start with the infection of 10 cells, and the cellular metabolic rates are homogeneous.

The most informative metric to distinguish effective from ineffective treatments is the amount of extracellular diffusive virus. This metric allows us to define subcategories of effective/ineffective treatment. Not surprisingly, we observe that larger $IC_{50}$ multipliers (lower potencies) and larger periods between doses result in higher levels of extracellular virus.

In this coarse investigation, we see that the simulated system transits from effective to ineffective treatment when the $IC_{50}$ multiplier changes from 0.05 to 0.1 (Figure 6). However, we do not see dosing interval effects here.

#### 3.3.2. Fine Parameter Variation

**Figure 7:**
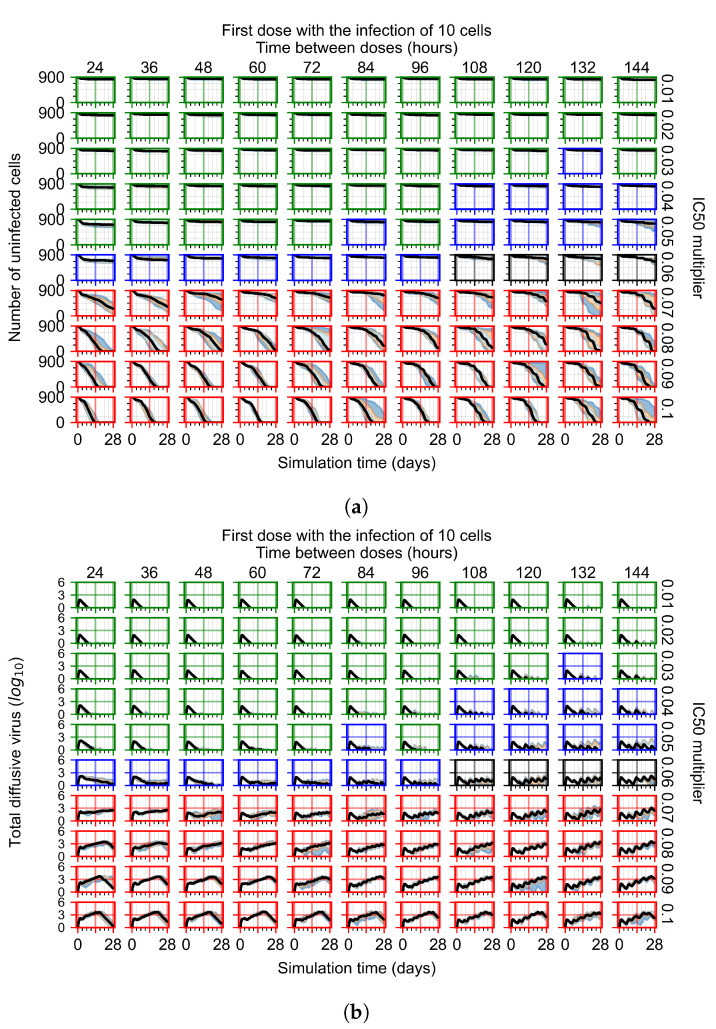
Treatment starts with the infection of 10 epithelial cells. For all subfigures, the median measurement of simulation replicas is the black line, the 0th to 100th quantiles are shaded as dark blue, 10th to 90th shaded in orange, and 25th to 75th as light blue. Rapid clearance plot axis in green, slow clearance plot axis in blue, partial containment in black, and widespread infection in red. (**a**) Uninfected cell population. (**b**) Extracellular diffusive virus; *y* axis in log scale, and exponent values as tick marks.

**Figure 8:**
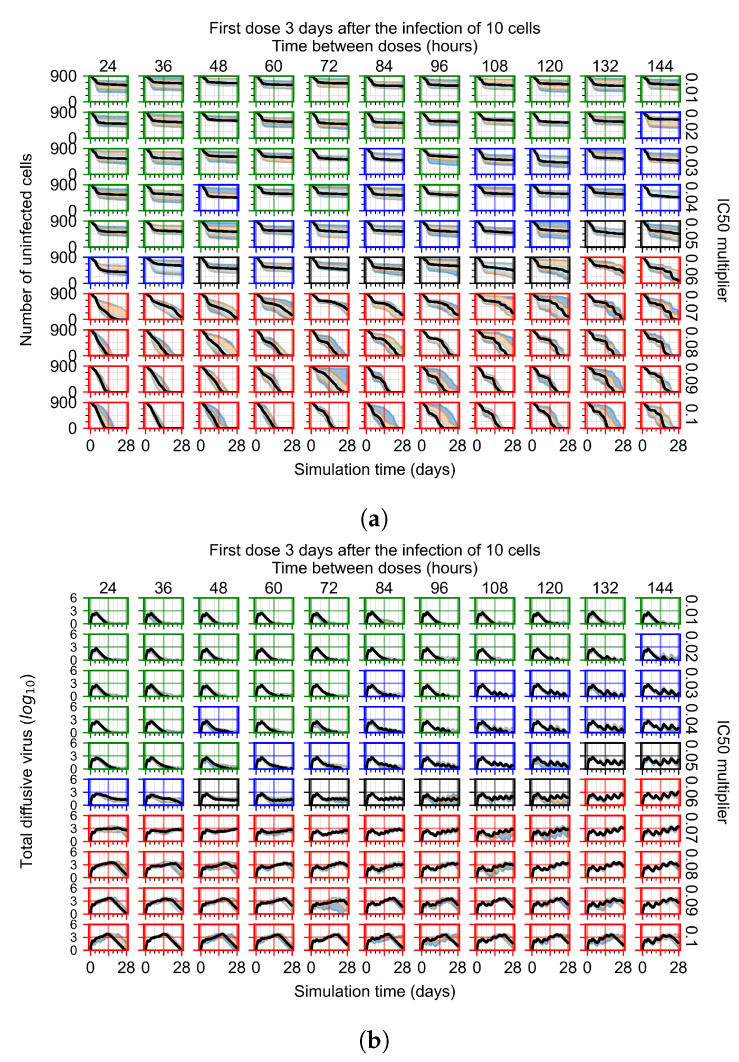
Treatment starts three days post the infection of 10 epithelial cells. For all subfigures, the median measurement of simulation replicas is the black line, the 0th to 100th quantiles are shaded as dark blue, 10th to 90th shaded in orange, and 25th to 75th as light blue. Rapid clearance plot axis in green, slow clearance plot axis in blue, partial containment in black, and widespread infection in red. (**a**) Uninfected cell population. (**b**) Extracellular diffusive virus; *y*-axis in log scale, and exponent values as tick marks.

After exploring the effects of coarse model behavior, we focus on the transition region between containment and no containment. $IC_{50}$ multipliers of 0.01, 0.02, 0.03, 0.04, 0.05, 0.06, 0.07, 0.08, 0.09, and 0.1 and dosing periods of 1, 1.5, 2, 2.5, 3, 3.5, 4, 4.5, 5, 5.5, and 6 days are explored (Figure 7 and Figure 8).

The results presented here are the measurements of uninfected population and diffusing virus for simulations where treatment starts with the infection of ten epithelial cells (Figure 7) and simulations where we delay treatment initiation by three days (Figure 8). For figures of the other metrics (number of dead cells, cytokine levels, etc.) and simulations where treatment starts 12 h and one day after the infection of ten epithelial cells; see Appendix F.1. We investigate the effects of diminishing GS-443902’s half-life by 50% and by 75% in Section 3.3.3 and Appendix F.2 and Appendix F.3. And the effects of heterogeneous metabolism of GS-443902 in Section 3.3.4 and Appendix F.4.

In Figure 7, there is no delay to treatment start. The system transits from effective treatment to ineffective treatment upon changing the $IC_{50}$ multiplier from 0.05 to 0.07. We also see the effects of drug dosing frequency; for the $IC_{50}$ multiplier of 0.05, the treatment outcome shifts from fast clearance to slow clearance when we change the scheduling period from 72 h to 108 h. For the $IC_{50}$ multiplier of 0.06, we see the transition from slow clearance to partial containment when we change the period from 96 h to 108 h.

In Figure 8, we delay treatment by three days. We can see that the system traverses from effective treatment to ineffective treatment upon changing the $IC_{50}$ multiplier from 0.04 to a multiplier of 0.06. Here, the effects of drug dosing frequency are more pronounced, with several transitions happening for a single $IC_{50}$ multiplier. For instance, with an $IC_{50}$ multiplier of 0.05, the simulated treatment moves from fast clearance to slow clearance to partial containment as time in-between doses becomes progressively longer.

In the cases of infrequent dosing or mid–high-potency treatments, we observe a situation in which extracellular virus concentrations fall below the “cleared” threshold and then reappear. The viral resurgence happens if infected cells remain and the level of remdesivir between doses falls to a point where viral replication can restart after the clearance of the extracellular virus. Even with reappearance, however, levels of the extracellular virus do not increase beyond the first peak in concentration.

We observe an effect that seems counter-intuitive for simulations with longer inter-dose periods and intermediate potencies. There is a more significant decrease in extracellular virus for the first few days of treatment, indicating a (temporarily) more effective treatment than for the same potency with more frequent dosing. This occurs because the duration of viral replication is constrained by the duration that the antiviral concentration is above the effective concentration that inhibits viral replication. This time period is longer for larger doses. For longer inter-dose intervals, the amount per dose is larger (for an inter-dose interval of *n* days, the dose $D^{\prime }$ is *n* times the amount of the standard dose (*D*), i.e., $D^{\prime }=n\times D$). Thus, the duration of inhibition of viral replication after a dose is longer for longer inter-dose intervals. The fact that the loading dose, $D_{L}$, is twice the regular dose, $D_{L}=2\times D^{\prime }$, makes this effect even stronger for the initial inter-dose period. In particular, it is possible that for a longer dose interval, the drug concentration never decreases below the effective concentration between the first and second doses, but decreases below the effective concentration during the intervals between all subsequent doses.

**Figure 9:**
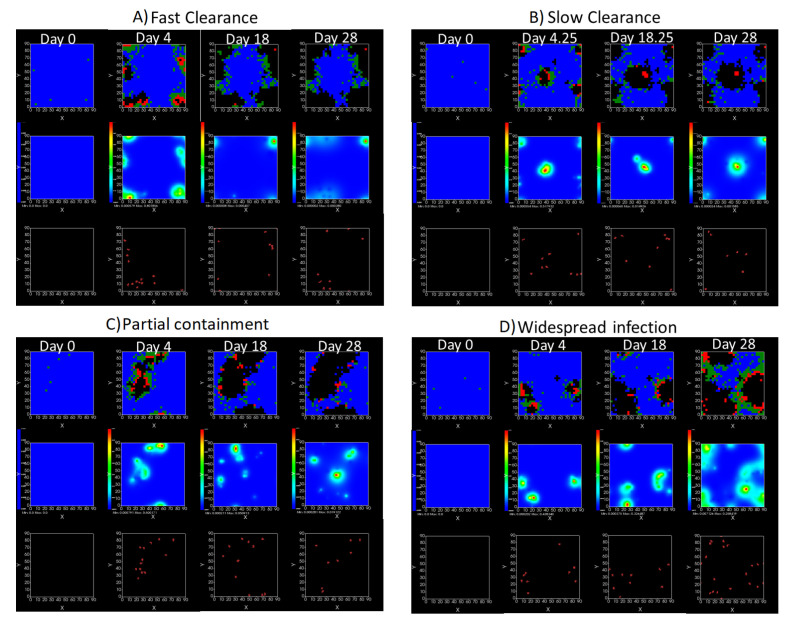
Replica snapshots of the tissue patch are shown for different treatment classifications. In all the cases, the top row is the epithelial layer (blue uninfected cells, green infected cells in eclipse phase, red infected cells releasing virus, black dead cells), the middle row is the extracellular virus concentrations (high concentration in red, low concentration in blue), and the third row is the immune cell layer (immune cells in red, extracellular environment in black). (**A**) Fast clearance (36 h dosing period, 0.01 $IC_{50}$ multiplier), (**B**) slow clearance (84 h dosing period, 0.05 $IC_{50}$ multiplier), (**C**) partial containment (84 h dosing period, 0.06 $IC_{50}$ multiplier), and (**D**) widespread infection (108 h dosing period, 0.07 $IC_{50}$ multiplier). In all the cases, snapshots are shown at the start of the simulation (day 0), at the start of treatment (3 days post infection of 10 cells), after 14 days of treatment, and at the end of the simulation (Day 28).

We show spatial configuration snapshots from replica simulations of the virtual tissue patches for the four classes in Figure 9.

#### 3.3.3. Faster Clearing Drug Necessitates More Potent Antiviral in Order to Contain the Infection

**Figure 10:**
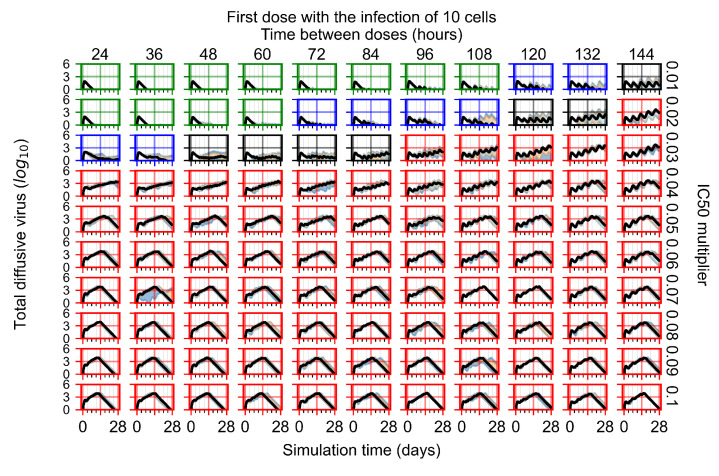
Extracellular diffusive virus populations for eight replicas of the treatment simulation. Treatment starts with the infection of 10 cells, the half-life of GS-443902 was halved (to 15.2 h). For all subfigures, the median measurement of simulation replicas is the black line, the 0th to 100th quantiles are shaded as dark blue, 10th to 90th shaded in orange, and 25th to 75th as light blue. Rapid clearance plot axis in green, slow clearance plot axis in blue, partial containment in black, and widespread infection in red.

We evaluated the effects of increased drug clearance on tissue outcomes. Specifically, we reduced the half-life of GS-443902 by 50% (from 30.4 h to 15.2 h) (see Figure 10 and Appendix F.2), or by 75% (to 7.6 h) (see Appendix F.3). With the faster clearing drug, we observe a general effect of shifting the region of effective treatment to greater potencies (smaller $IC_{50}$ multiplier); however, there is no similar shift towards more frequent doses schedules.

With the half-life of GS-443902 reduced to 15.2 h and no delay to treatment initiation, we classify many more treatments as ineffective than treatments using the regular half-life and delaying treatment by three days.

Initiating treatment later, at one or three days post-infection of ten epithelial cells, pushed outcomes to widespread infection (see Appendix F.2). However, as most treatments are already ineffective with the faster clearing antiviral, few options were pushed to ineffectiveness.

#### 3.3.4. Heterogeneous Cellular Metabolism of Remdesivir Results

Now we vary the metabolic rates of the antiviral in each epithelial cell individually, as detailed in the Methods Section 2.5. We see that heterogeneous drug metabolism and clearance result in an overall worse treatment outcome. We believe this occurs due to infection being driven forward by the cells that generate the most extracellular virus; we test this hypothesis in Section 3.3.5. If there is a region of cells with good drug metabolism (the antiviral is effective) but one among them is insensitive, the infection will progress.

**Figure 11:**
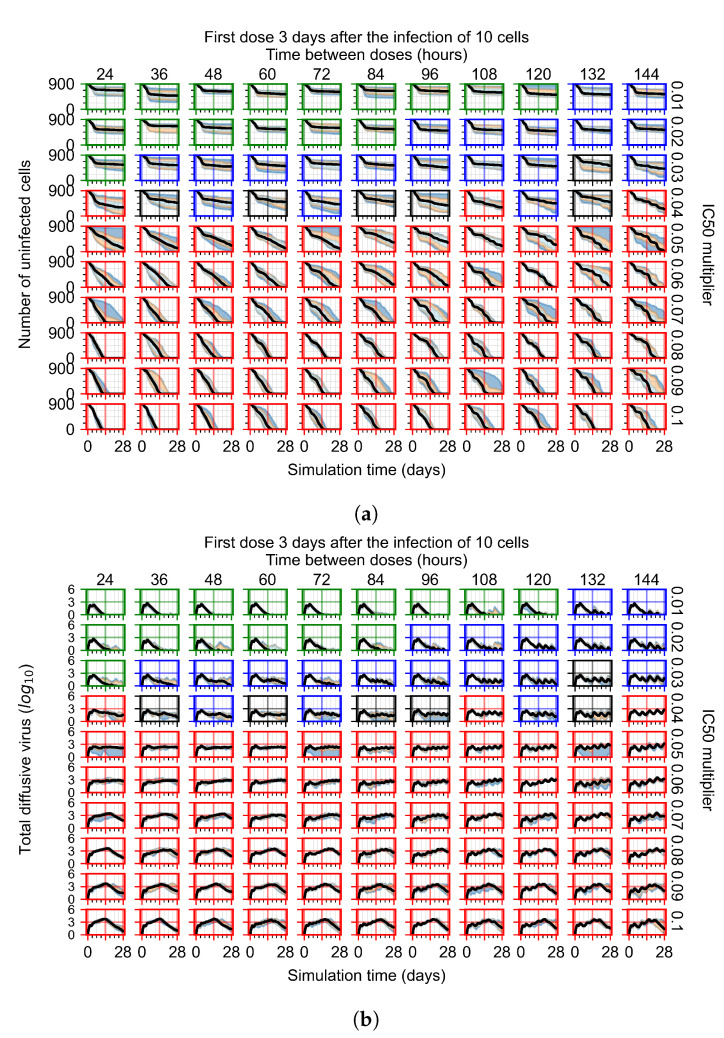
Extracellular diffusive virus populations for eight replicas of the treatment simulation. Epithelial cells’ metabolism and clearance varies from cell to cell. For all subfigures, the median measurement of simulation replicas is the black line, the 0th to 100th quantiles are shaded as dark blue, 10th to 90th shaded in orange, and 25th to 75th as light blue. Rapid clearance plot axis in green, slow clearance plot axis in blue, partial containment in black, and widespread infection in red. (**a**) Treatment starts with the infection of 10 cells. (**b**) Treatment starts three days post the infection of 10 cells.

For the heterogeneous cellular metabolism simulations, treatment shifts from effective to ineffective when we change the $IC_{50}$ multiplier from 0.03 to 0.05 (Figure 11) instead of 0.04 to 0.06 for the homogeneous case (Figure 8), both when there is no delay to treatment initiation and with delay. However, when there is no delay, the 0.05 $IC_{50}$ multiplier results in mostly partial containment, and the 0.03 $IC_{50}$ multiplier guarantees clearance. When we delay treatment by three days, 0.05 $IC_{50}$ multiplier results in mostly widespread infections, and the 0.03 $IC_{50}$ multiplier is close to the slow clearance–partial containment transition. Even the best-case scenario, starting treatment with the infection of ten epithelial cells with heterogeneous metabolism, yields worse outcomes than the worst-case scenario, starting treatment three days after the infection of 10 epithelial cells, for the homogeneous case.

For the heterogeneous cell response simulations, changing treatment initiation time has a more pronounced effect than the homogeneous case, see Figure 11 and Appendix F.4.3. For instance, when we delay treatment by 1 or 3 days (Figure 11b and Appendix F.4.3), most treatments for an $IC_{50}$ multiplier of 0.3 become almost ineffective (slow clearance) from most being classified as fast clearance when there is no delay (Figure 11a). For an $IC_{50}$ multiplier of 0.05, we classify all except one treatment option as partial containment with no delay (Figure 11a). In contrast, we classify most infection dynamics as widespread infection with a delay of 1 or 3 days (Appendix F.4.3, Figure 11b).

#### 3.3.5. Factors Responsible for Negative Treatment Outcomes in the Heterogeneous Metabolism Model

As cellular heterogeneous metabolic response worsens treatment outcomes, we perform simulations tracking viral production (viral AUC) by individual cells. We hypothesized that the infection is driven forwards by the cells that are least sensitive to the antiviral. We selected four parameter combinations used in Figure 11b (i.e., simulations with treatment delayed by three days), one from each classification, to perform simulations investigating the viral production by individual cells. The parameter sets chosen are:

- Rapid clearance: 24 h dose interval, 0.01 $IC_{50}$ multiplier;
- Slow clearance: 120 h dose interval, 0.03 $IC_{50}$ multiplier;
- Partial containment: 96 h dose interval, 0.06 $IC_{50}$ multiplier;
- Widespread infection: 24 h dose interval, 0.1 $IC_{50}$ multiplier.

**Figure 12:**
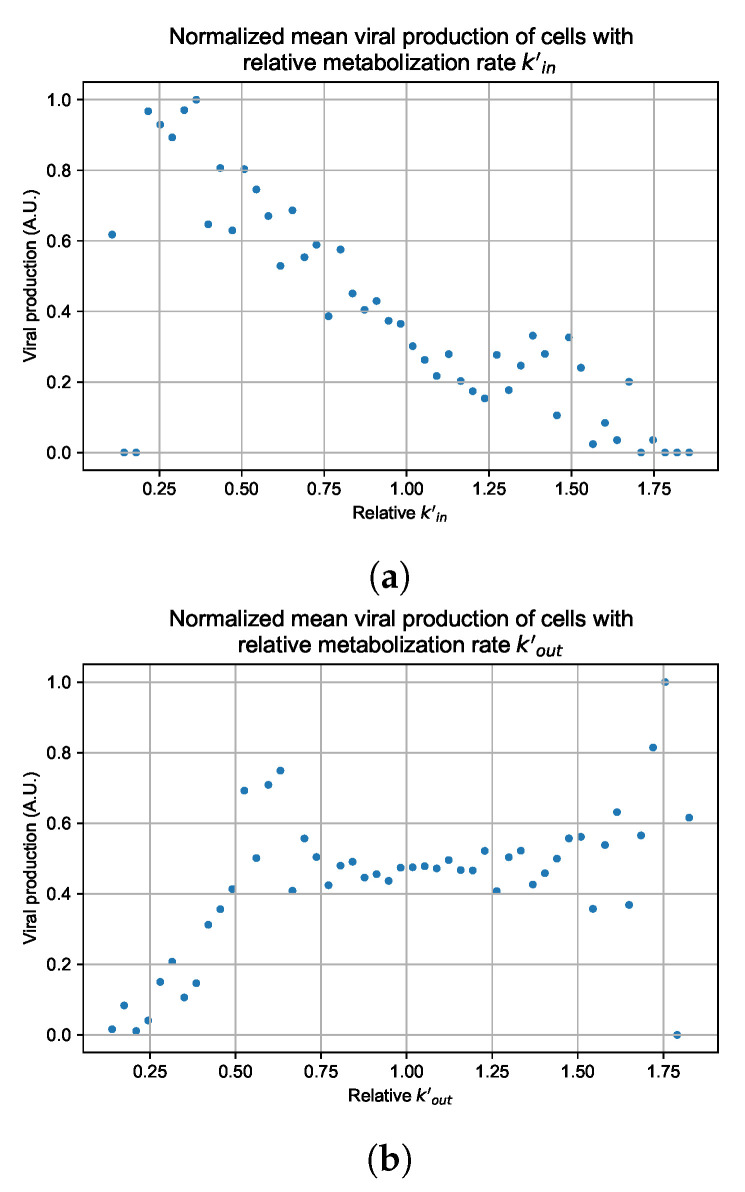
Mean viral production of cells versus their relative metabolic rates normalized by the maximum mean production with partial containment parameters. (**a**) Results for simulations varying only $k_{in}$, uses the partial containment parameter set. (**b**) Results for simulations varying only $k_{out}$, uses the widespread infection parameter set.

In these simulations, we vary either $k_{in}$ (Figure 12a) or $k_{out}$ (Figure 12b). We measure the per-cell viral production and see the production–metabolic rate relationship. If our hypothesis is correct, we would expect with $k_{in}$ increase, the average intra-cellular concentration of antiviral increases subsequently, and the viral production would decrease. For $k_{out}$, we would expect the opposite correlation. With increasing $k_{out}$, the intra-cellular viral concentration decreases and viral production will increase.

As before, we run four simulation replicas for each parameter set. We then combine the replicas’ per cell viral production data, separate the cells into 50 bins of the metabolic rate range (either $k_{in}$ or $k_{out}$), and calculate the mean production in each bin.

Figure 12 shows the results for the parameter sets that had the most evident correlation. Namely the partial containment set for $k_{in}$ and widespread infection set for $k_{out}$. We see that the hypothesis of super spreader cells is reasonable, as the correlation of metabolic rates and viral production agrees as we would hypothesized. As $k_{in}$ increases, viral production decreases and as $k_{out}$ increases, viral production increases. Outliers are escaping the trend at the limits of the metabolic rates. Several factors explain these outliers, such as the time a cell releases virus before dying. On top of that, the distribution of rates is not uniform but a normal distribution (see Section 2.5). As the number of cells with either high or low metabolic rates will be low, the *mean production* of cells with high or low metabolic rates is more likely to have fluctuations.

The least sensitive cells to the antiviral drive forward the infection and complement a recent result from Reinharz et al. [49]. They have shown that cellular heterogeneity in interferon signaling response has the effect of improving tissue outcomes. Antiviral resistance due to interferon depends on the cells that produce the most interferon (effectively warning other cells of the infection).

For the other parameter sets, see Appendix G. As there are several sources of heterogeneity in the model, the antiviral-metabolic rate vs. viral production correlation can be less apparent, which is the case for the results in Appendix G. For instance, the correlation is weak if the infection spreads fast (killing virus producing cells early) or is contained quickly (making the number of infected cells low). Weak correlation can also arise from the infected cells dying before or shortly after releasing the virus to the environment.

#### 3.3.6. Effects of Variability in Cellular Drug Metabolism on Treatment Outcomes

**Figure 13:**
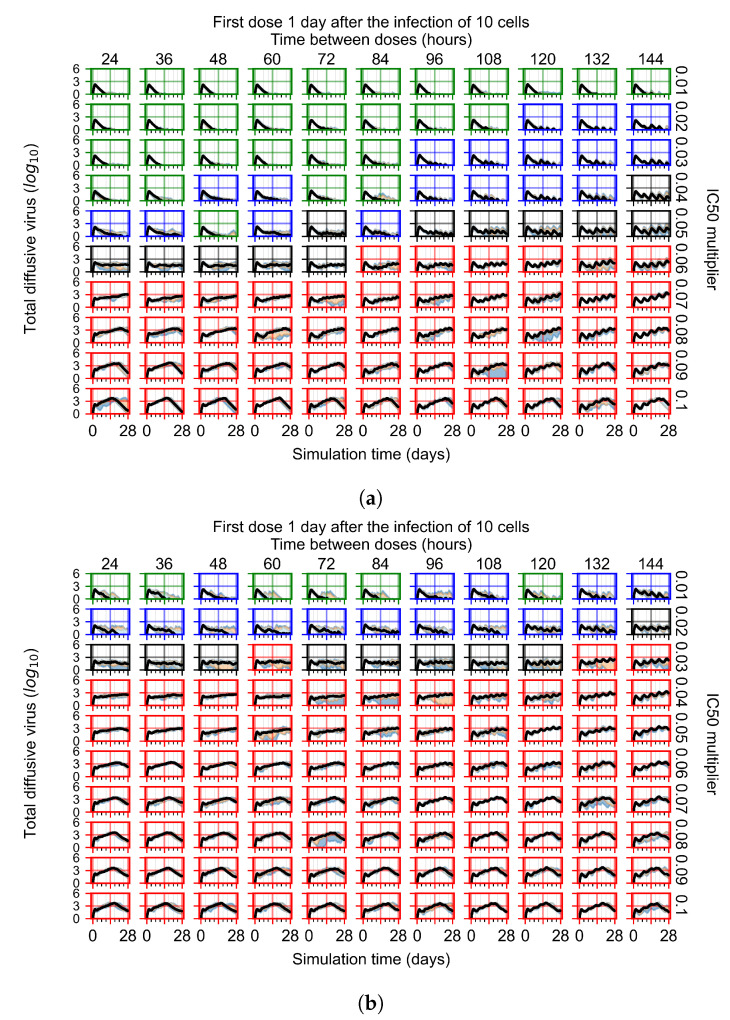
Extracellular diffusive virus populations for eight replicas of the treatment simulation. Epithelial cells’ metabolism and clearance varies from cell to cell. For all subfigures, the median measurement of simulation replicas is the black line, the 0th to 100th quantiles are shaded as dark blue, 10th to 90th shaded in orange, and 25th to 75th as light blue. Rapid clearance plot axis in green, slow clearance plot axis in blue, partial containment in black, and widespread infection in red. (**a**) Cells’ metabolism standard deviation set to 0.1. (**b**) Cells’ metabolism standard deviation set to 0.5.

**Figure 14:**
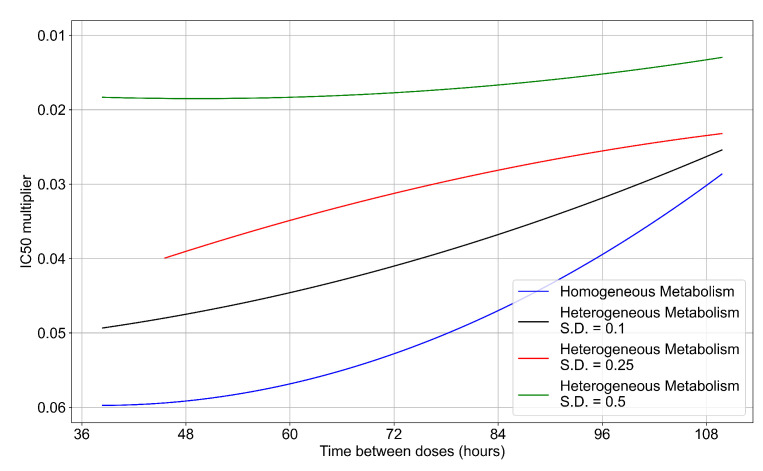
Ineffective–effective treatment transition border for different levels of metabolic variability. In blue is the homogeneous metabolism case, in black the heterogeneous metabolism case applying a modulation by a Gaussian random number with standard deviation (S.D.) set to 0.1 for $k_{in}$ and $k_{out}$, in red the heterogeneous case with the Gaussian random number S.D. set to 0.25, in green with the S.D. set to 0.5.

To quantify the effects of variability in cellular drug metabolism on treatment outcome, we simulated tissues with different levels of variability. This was performed by changing the modulation parameter of the metabolic rates, $k_{in}$ and $k_{out}$ in Equations (12) and (14). For the results so far, in Equations (12) and (14) we modulated the metabolic rates by drawing a random number from a Gaussian distribution with mean (μ) set to 0 and standard deviation (ξ) set to 0.25. Here, we perform simulations with ξ=0.1 (see Figure 13a) and ξ=0.5 (see Figure 13b). With a ξ=0.1 most treatment outcomes that are classified as rapid or slow clearances in the homogeneous case remain effective. In contrast, with a ξ=0.5, almost all treatments are ineffective. We map the boundary of effective-ineffective treatments (Figure 14) by plotting the centers of simulations classified as slow clearance. We observe that the boundary moves towards lower $IC_{50}$ multipliers (i.e., more potent drugs would be needed). We also saw that the increase in metabolic variability made the boundary less dependent on the dosing interval. Other metrics for these simulations can be found in Appendix F.5. In summary, with a lower variability of cell metabolism, treatment outcomes were closer to those of uniform cell metabolism; whereas, with a higher variability most treatments became ineffective.

**Figure 15:**
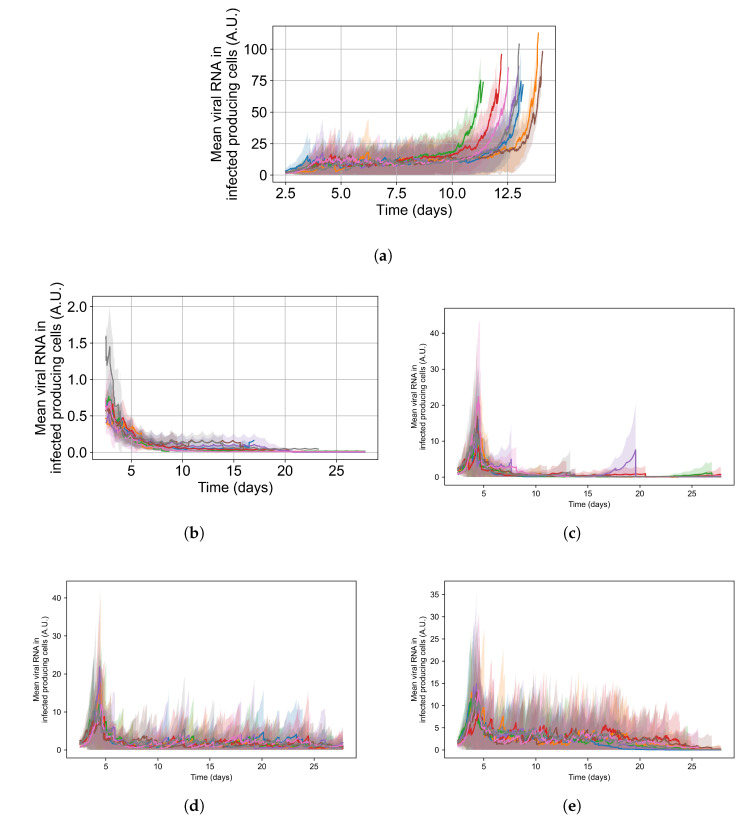
Mean RNA levels in virus-releasing infected cells (solid lines) with standard deviation (shaded regions) versus time for individual simulation replicates under different simulation options: (**a**) is untreated, (**b**) is treated with no metabolic heterogeneity, (**c**) is treated with a metabolism standard deviation of 0.1, (**d**) is treated with a metabolism standard deviation of 0.25, and (**e**) is treated with a metabolism standard deviation of 0.5. Please note that the y-range in (**a**,**b**) differs from the others.

To appreciate the effects of cellular metabolic heterogeneity on treatment outcome, we compare intra-cellular viral RNA levels in untreated cases to the same with treatment at different levels of heterogeneity (i.e. different ξ). Quantifying intra-cellular RNA is not straightforward, as the time of infection of different cells varies, and the total number of infected cells at a given time also varies. Therefore, we consider how to aggregate cells and measure intra-cellular RNA. We opt to measure the viral RNA levels only for infected cells that release virus to the extra cellular environment (i.e., that have exited the eclipse phase of infection), as they are further along in disease progression and will have the highest intra-cellular RNA levels. It is also important to note that growth of RNA level in cells under ineffective or no treatment is exponential; therefore, mean RNA levels can be dominated by a small number of cells until they perish due to viral production stress. We see such cases as spikes in RNA levels followed by a rapid decay in Figure 15.

For the intra-cellular RNA-level comparison, we have one set of simulations with no treatment at all; and for the treated simulations, we choose a set of treatment parameters that yielded results classified as rapid clearance when there is no metabolic heterogeneity, as slow clearance with the metabolic heterogeneity ξ set to 0.1, as partial containment with ξ=0.25, and as widespread infection with ξ=0.5. Chosen treatment parameters are: delay treatment for one day, dose every 24 h, $IC_{50}$ multiplier of 0.05.

In Figure 15, we show the mean and standard deviation of RNA levels in virus-producing cells for 5 sets of simulations with the simulation replicas of each set plotted individually, both for the untreated case (see Figure 15a) and with treatment (see Figure 15b–e. We want to point out that the scales of Figure 15’s subfigures are not the same, so that the change in RNA level can be seen for all cases.

Without any treatment, mean RNA levels in virus-producing cells stay stable at a level of 12 arbitrary units until the end of the simulation, when RNA levels in the few surviving epithelial cells shoot up. Before that time, there are cells with high levels of internal viral RNA but the mean level is brought down by cells that are earlier in the infection stage. With treatment and no inter-cellular metabolic heterogeneity the intra-cellular RNA levels in virus-producing cells stay low, below 2 A.U., for the entire treatment duration and RNA levels among cells are similar (low standard deviation). We also see that as treatment progresses, the mean RNA levels steadily decrease, and we see a periodic effect from the dosing interval. This is to be expected when treatment is effective and all cells respond to treatment identically (see Figure 15b). As we increase metabolic heterogeneity (ξ), the mean intra-cellular RNA levels also increase as does the variation of intra-cellular RNA levels among cells. We also start to see spikes in the mean intra-cellular RNA levels related to cells that do not control viral replication internally (one spike for ξ=0.1, several for ξ>0.1), where in those cells the RNA growth is exponential. As soon as those cells die, the mean intra-cellular RNA drops back down to a steady level. When we use ξ=0.5 in Equations (12) and (14), the RNA levels oscillate in a similar range to that of the untreated case. In Appendix F.5.3, we show how changing the metabolic heterogeneity affects the average and distribution of intra-cellular drug levels in these simulations. In short, when ξ=0.1, intra-cellular drug levels stay close to the homogeneous metabolism case. With ξ=0.25, intra-cellular drug concentrations are more spread out, and with some cells at levels that are double or half the mean. With ξ=0.5, there are cells with 0 intra-cellular drug concentration and cells with intra-cellular concentrations as much as 3-fold greater than the mean.

## 4. Discussion

Our model of antiviral treatment of COVID-19 integrates a spatio-temporal model of an infected lung tissue patch with remdesivir PK and PD models. This approach allows us to probe the distribution, dynamics, and effects of remdesivir within a hypothetical pathological tissue structure.

This work is part of a collaborative effort. The modeling framework is extendable and modular. Models of other viruses and therapies can replace our viral model and PK-PD models.

Computational methods are a necessary complement to experimental efforts moving forward in the fight against COVID-19, future pandemics and current diseases. The combined complexities of the pathogen, disease pathology, immune response (both innate and adaptive), antiviral dynamics, and host variation, including variation in the basics units of the body, make it virtually impossible to disentangle the numerous driving forces behind infection outcomes using only animal and human data. Furthermore, experimental data are often sparse, and computational models can expand the explanatory power of limited experimental data. Our modular approach captures and integrates these dynamics to help translate biomedical mechanisms to clinical relevance.

We characterize the infection and treatment dynamics of an epithelial patch infected by SARS-CoV-2 and treated with remdesivir, at dose regimens encompassing those approved clinically. To create our model, we used reported human pharmacokinetics of remdesivir and its active metabolite and physiologically relevant within-host viral dynamics. We have considered the PK model profile of remdesivir to calculate the concentration of the drug in each cell, while assuming, at first, that the availability of drug to each cell is equal (well–mixed conditions). The lung tissue concentrations of all of the remdesivir metabolites are not evident in any reported biomedical study; therefore, we estimated them from their plasma concentrations using pharmacokinetic models [7,39,50].

We simulate multiple regimens for antiviral therapy on top of our extendable framework. We aimed to explore if the unconvincing results of antiviral trials and their clinical use could be explained by exploring the effects of changes in drug potency and schedule and some unknown possibilities. We found that the same dosing regimen and the same parameter set different replicas can have different outcomes. Furthermore, we found that if the cellular metabolism of the antiviral changes from cell to cell, simulation outcomes are more dispersed, and the effective antiviral dose is greater. Altogether, that means that two identical patients receiving the same identical treatment could have different outcomes. We believe this explains some of the ambiguity of the clinical trials.

The spatial model enables exploration of the effects of inter-cellular heterogeneous response to the antiviral, even with a lack of experimental data on how different cells of the same tissue may react and metabolize drug compounds differently. Therefore, we perform simulated experiments to investigate how that variation in metabolism may affect treatment outcomes. Our model predicts that cells that are less sensitive to the drug drive the infection, necessitating higher antiviral doses or the need for a more potent antiviral activity (by >50% depending on level of heterogeneity and treatment delay) (see Section 3.3.2, Section 3.3.4, and Section 3.3.6). Therefore, experimental studies that investigate what are the typical metabolic variations in a tissue would enable the development of models that fit even better with reality. A pure population model of infection would not predict that cellular heterogeneity increases the antiviral potency needed for effective control of the infection, as they cannot model inter-cellular heterogeneity in space.

As expected, the model predicted that remdesivir exposure, both to adequate doses of antiviral and in the amount of time with adequate levels, is a crucial determinant of treatment outcome, implying that increased dose amount would improve treatment outcome. We show that suboptimal exposure to the simulated antiviral inside simulated infected epithelial tissues leads to regrowth of viral load between doses and may contribute to persistent COVID-19. This result aligns with the reported experiments in non-human primate, rhesus macaques [15,16,17].

However, we do not model drug-induced toxicity, which is a concern at high doses and limits the clinically safe dose. We also predicted the continued significance of host mechanisms during treatment, such as metabolic clearance of the antiviral (see Section 3.3.5). Identification and ranking of these host mechanisms identifies potential targets for therapy development [51,52]. Potential strategies include treatment utilizing a neutralizing antibody cocktail or convalescent plasma to boost host immunity or fast viral clearance. Here, we want to comment that the present study is a mathematical and mechanistic exploration of possible scenarios. The present model simulates a tiny patch of epithelial tissue and cannot predict clinical outcomes at the whole-body level. The present model lacks a well-defined immune response that allows natural clearance of the virus. The antibody-mediated viral neutralization and antibody-dependent cellular cytotoxicity is not considered in the present model, hence the second, rapid clearance phase is not observed in the viraemic loads of our simulations. We also recognize that the present set of simulations aggregate rather than explore, the different sources of heterogeneity and variability. Future work should explore the sources of heterogeneity, incorporate the antibody therapies with an improved, well-defined immune response model, as well as of tissue recovery. In turn, improved immune response modeling will help us determine the cross-talk between antibody cocktails with the host immune response while reducing the viral burden. Because of these results, we believe that researchers must consider host mechanisms, viral load, and drug permeability as part of the design space combining immuno-modulation and antiviral treatment of COVID-19 and other viral diseases.

The main finding of this paper is the effects of heterogeneity in cellular metabolism (uptake and clearance) on viral replication. Even in the presence of spatial stochasticity, identical cellular behavior predicted better outcomes for the antiviral treatment. In contrast, including cellular heterogeneity worsened the treatment outcomes and produced results that are more similar to previous clinical trial outcomes than the homogeneous setup [53]. This discrepancy of model prediction and clinical trials may be a limitation of traditional pharmacometrics models that utilize the well-stirred assumption. As employed in this study, incorporating tissue heterogeneity may be important to improve the clinical trial simulation.

Clinical trials of antivirals for COVID-19 remain fraught with limitations, including the inability to test drugs singularly or in combinations, high cost, and the long duration of clinical trials. The most significant limitation of antiviral trials as treatment is that antivirals need to be given early in infection, which is a challenging issue for SARS-CoV-2, where there is an appreciable delay between infection and symptom onset. Animal models play an essential role in identifying new and effective regimens, but these studies are also time-consuming and costly, and they require models with human-like pathology. Here, we provide a complementary systems pharmacology tool for predicting the efficacy of new drugs and regimens, allowing a rapid assessment of drug efficacy at the site of viral infection while considering cellular heterogeneity.

## Figures and Tables

**Table 1:** List of parameters used for the minimal PBPK model of remdesivir.

Parameter	Value	Source
$k_{in}$ (unit-less)	0 or 1	Fit to [7]
GS-443902 observed Half-life, $t_{1/2}$ (h)	30.2	[7]
$k_{out}$, GS-443902’s decay rate (1/h)	$ln\left(2\right)/t_{1/2}$	
$D_{rmd}$ (mg/day)	100 (200 for loading dose)	Doses used in clinical situations
vol (L)	38.4	Fit to [7,39]
$\tau_{I}$ (h)	1	

**Table 2:** List of parameters for the ABM and PD models as well as parameters varied for the treatment effectiveness investigation.

Parameter	Values Used
Total epithelial population	900
Number of initially infected cells	5
Treatment initiation delay (day)	0, 1, 3
Time between antiviral doses	8, 12, 24, 36, 48, 60, 72, 84, 96, 108, 120, 132, 144
Remdesivir doses (rescaled to match the schedules) (mg)	25, 50, 100, 150, 200, 250, 300, 350, 400, 450, 500, 550, 600
Base $IC_{50}$ (μmol/L)	7.897
Viral replication rate reduction (Equation (11)) Hill coefficient	2
$IC_{50}$ multipliers	0.01, 0.02, 0.03, 0.04, 0.05, 0.06, 0.07, 0.08, 0.09, 0.1, 0.5, 1, 5, 10

**Figure A2:**
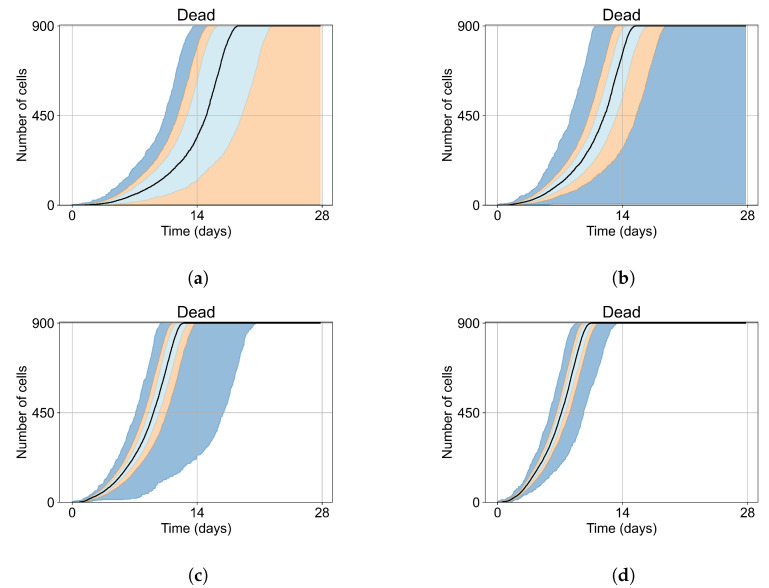
Dead cell populations for 400 replicas of Sego et al.’s model performed by us [24]. In all the cases, the medians of simulation replicas are in black lines, the 0th to 100th quantiles are shaded as dark blue, 10th to 90th shaded in orange, and the 25th to 75th as light blue. (**a**) Simulations start with 1 initially infected cell and 7 simulations result in failure to infect (1.75% of replicas), the 90th quantile includes the upper bound of the number of cells. (**b**) Simulations start with 2 initially infected cells where 5 simulations result in failure to infect (1.25%), the 100th quantile includes the upper bound of the number of cells. (**c**) Simulations start with 5 initially infected cells. (**d**) Simulations start with 10 initially infected cells.

**Figure A3:**
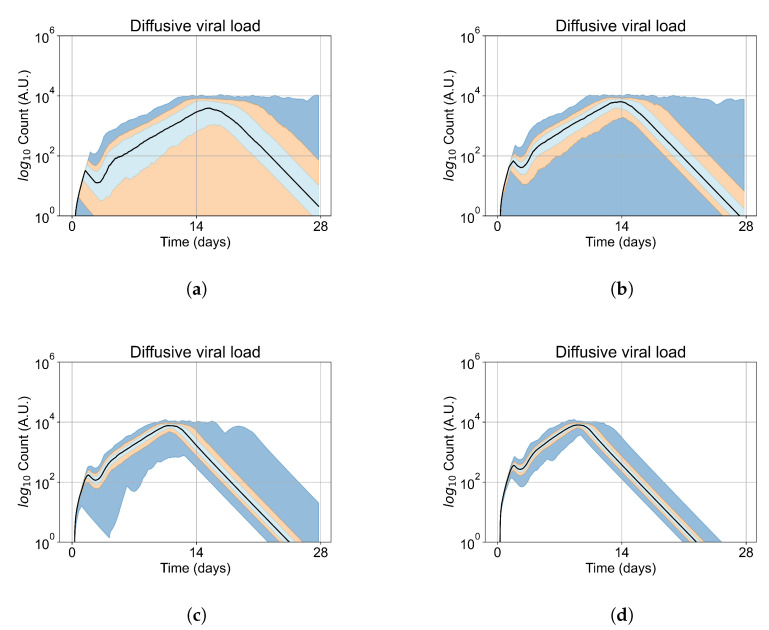
Extracellular viral load for 400 replicas of Sego et al.’s model [24], y-axis in log scale. In all the cases, the medians of simulation replicas are in black lines, the 0th to 100th quantiles are shaded as dark blue, 10th to 90th shaded in orange, and the 25th to 75th as light blue. (**a**) Simulations start with 1 initially infected cell and 7 simulations result in failure to infect (1.75% of replicas), the 90th quantile includes the upper bound of the number of cells. (**b**) Simulations start with 2 initially infected cells where 5 simulations result in failure to infect (1.25%), the 100th quantile includes the upper bound of the number of cells. (**c** Simulations start with 5 initially infected cells. (**d**) Simulations start with 10 initially infected cells.

**Figure A4:**
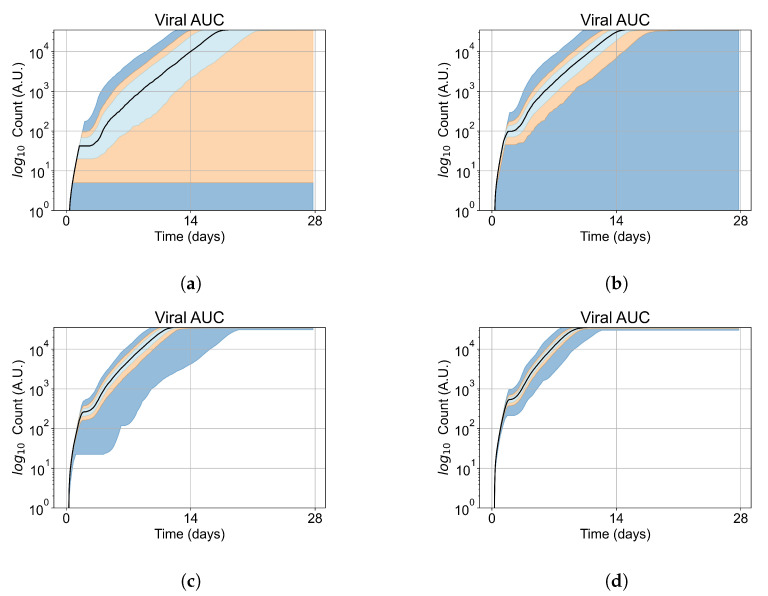
Extracellular viral AUC for 400 replicas of Sego et al.’s model performed by us [24], y-axis in log scale. In all the cases, the medians of simulation replicas are in black lines, the 0th to 100th quantiles are shaded as dark blue, 10th to 90th shaded in orange, and the 25th to 75th as light blue. (**a**) Simulations start with 1 initially infected cell and 7 simulations result in failure to infect (1.75% of replicas), the 90th quantile includes the upper bound of the number of cells. (**b**) Simulations start with 2 initially infected cells where 5 simulations result in failure to infect (1.25%), the 100th quantile includes the upper bound of the number of cells. (**c**) Simulations start with 5 initially infected cells. (**d**) Simulations start with 10 initially infected cells.

**Figure A5:**
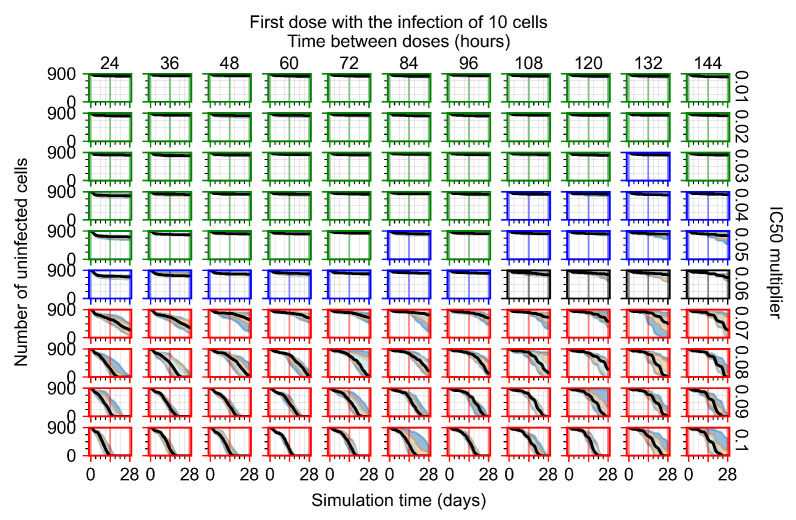
Uninfected population.

**Figure A6:**
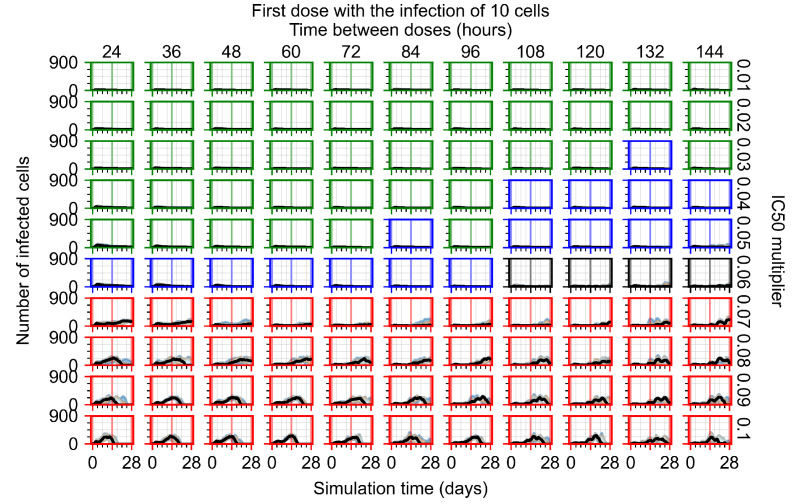
Infected (eclipse phase) population.

**Figure A7:**
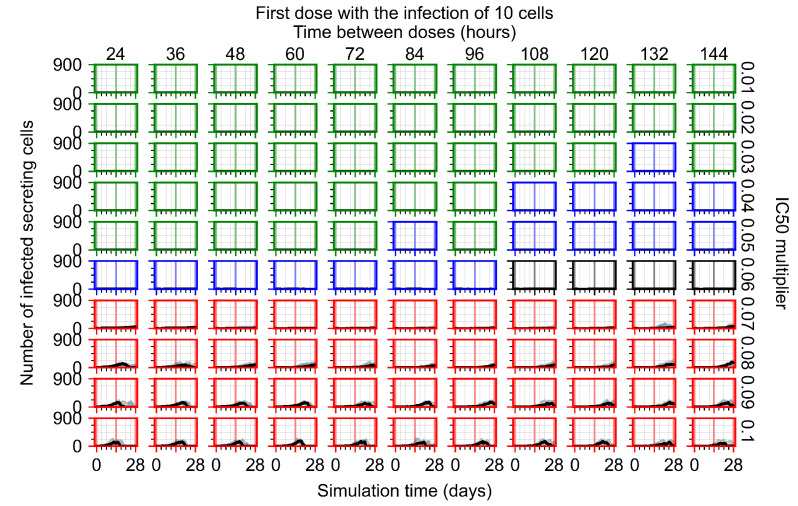
Infected (secreting extracellular virus) population.

**Figure A8:**
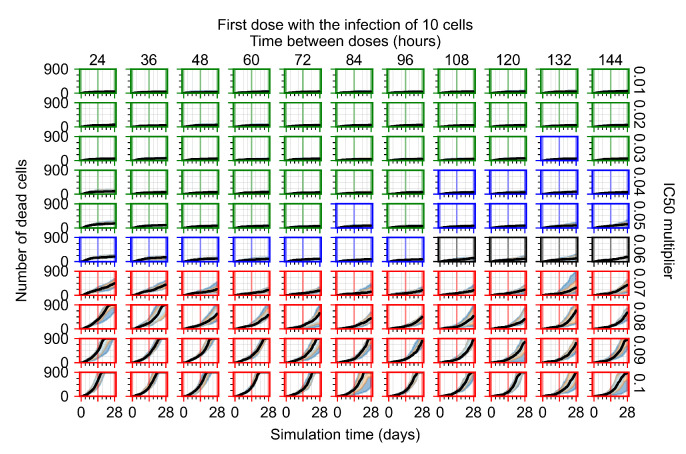
Dead population.

**Figure A9:**
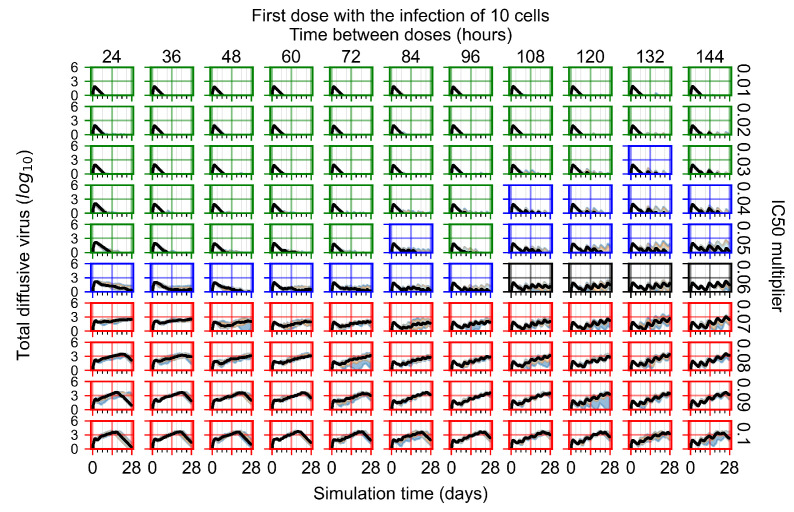
Extracellular diffusive virus quantities (arbitrary units), Y axis in log scale, and exponent values as tick marks.

**Figure A10:**
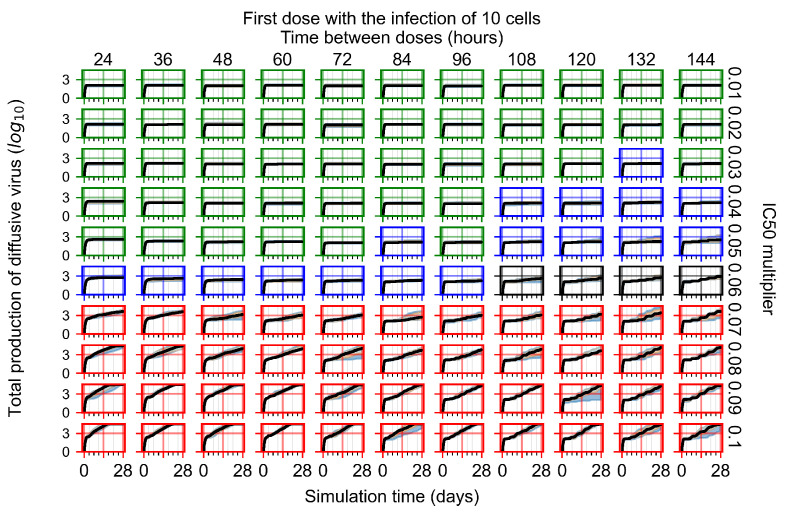
Total diffusive virus produced (AUC), Y axis in log scale, and exponent values as tick marks.

**Figure A11:**
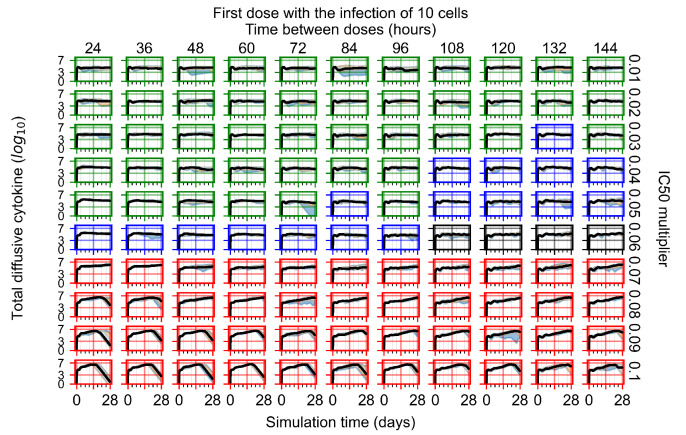
Diffusive cytokine amount, Y axis in log scale, and exponent values as tick marks.

**Figure A12:**
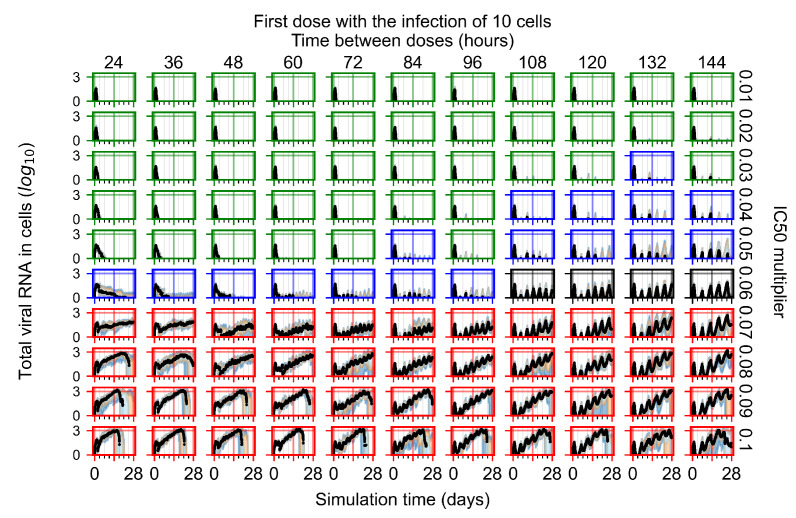
Amount of viral RNA in infected cells (arbitrary units), Y axis in log scale, and exponent values as tick marks.

**Figure A13:**
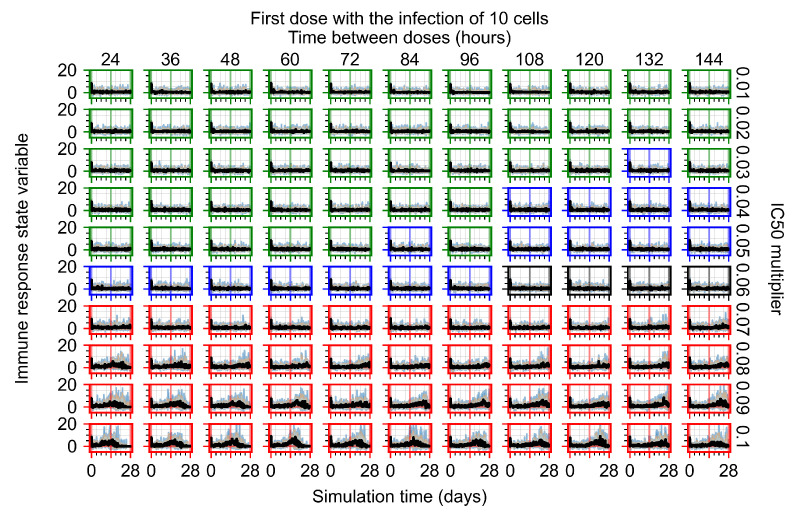
Immune response state variable number. Positive values correspond to an inflammatory state, and negative to an anti-inflammatory state.

**Figure A14:**
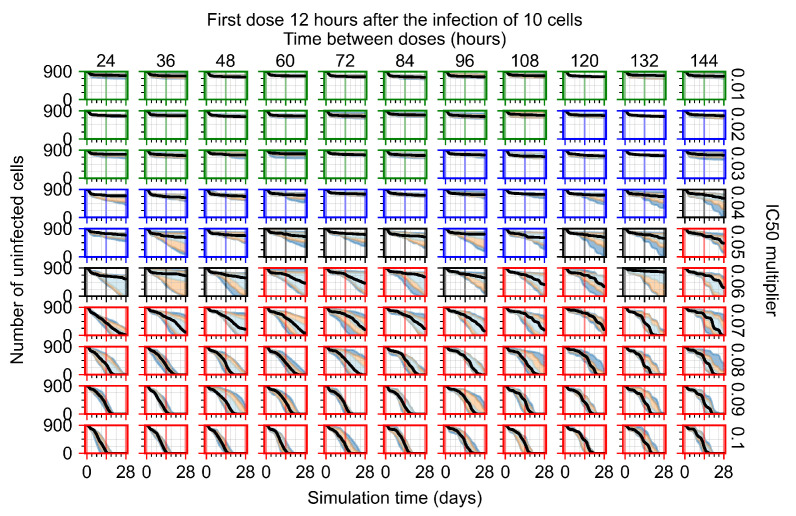
Uninfected population.

**Figure A15:**
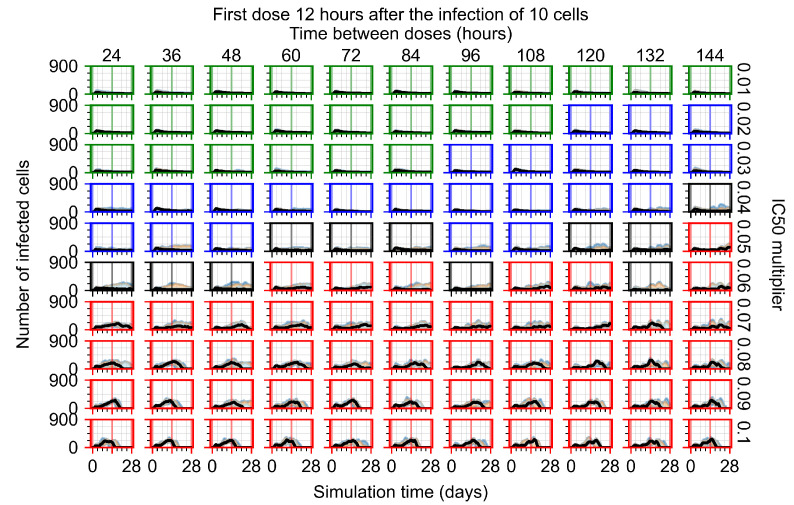
Infected (eclipse phase) population.

**Figure A16:**
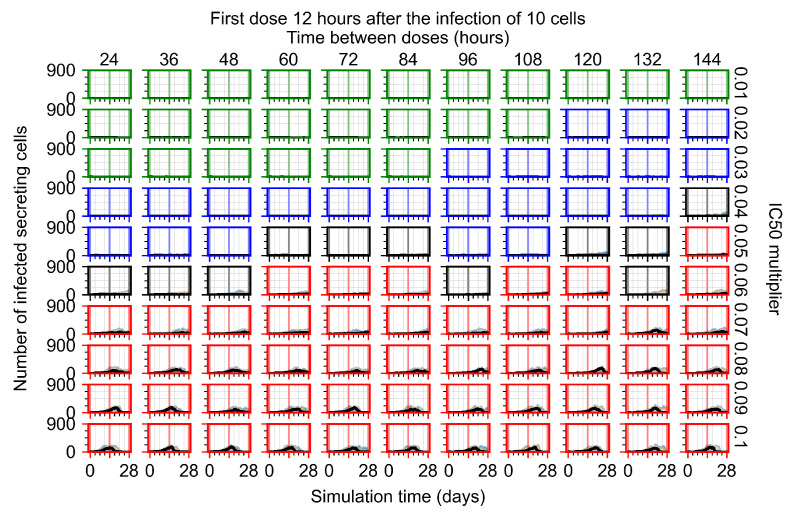
Infected (secreting extracellular virus) population.

**Figure A17:**
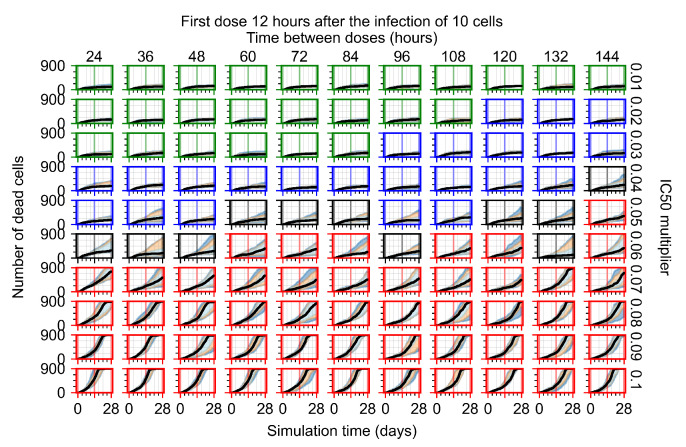
Dead population.

**Figure A18:**
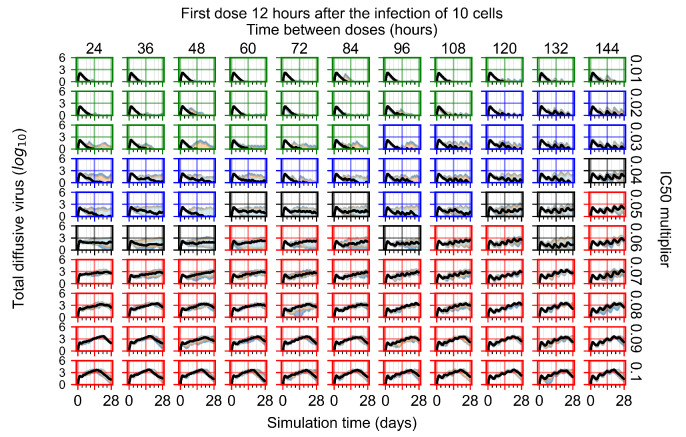
Extracellular diffusive virus quantities (arbitrary units), Y axis in log scale, and exponent values as tick marks.

**Figure A19:**
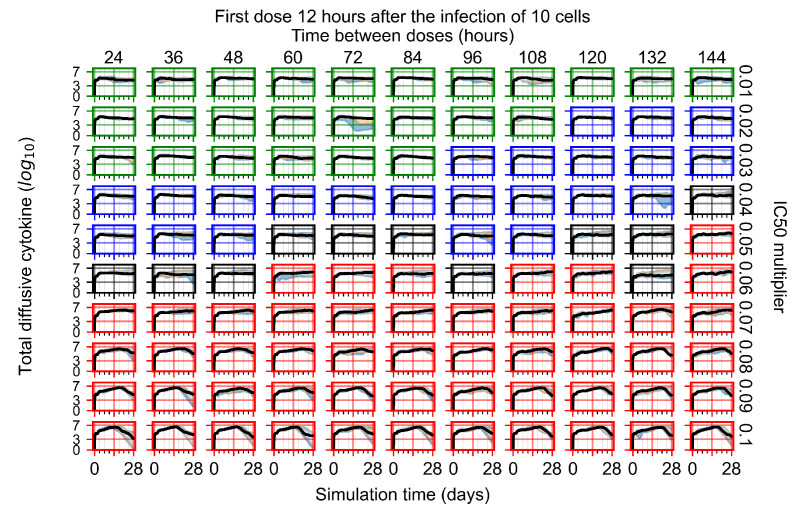
Diffusive cytokine amount, Y axis in log scale, and exponent values as tick marks.

**Figure A20:**
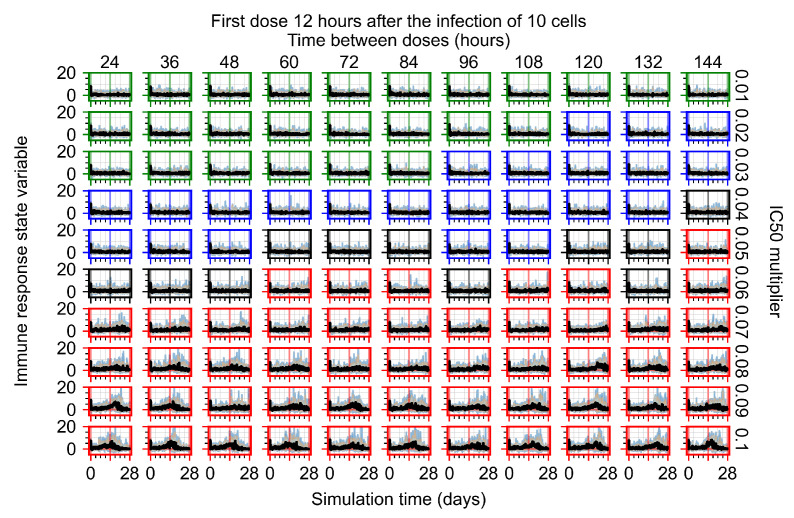
Immune response state variable number. Positive values correspond to an inflammatory state, and negative to an anti-inflammatory state.

**Figure A21:**
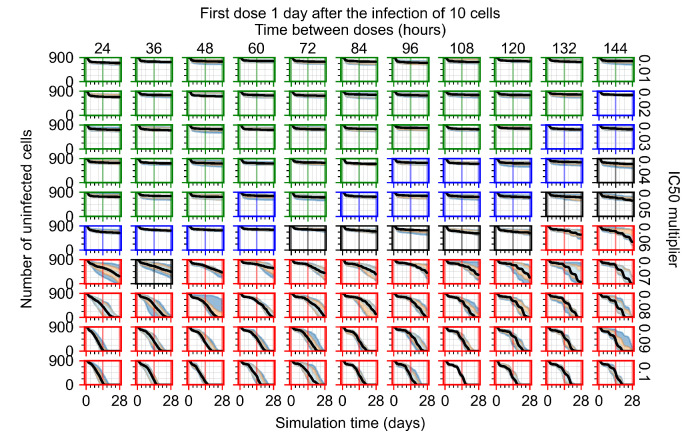
Uninfected population.

**Figure A22:**
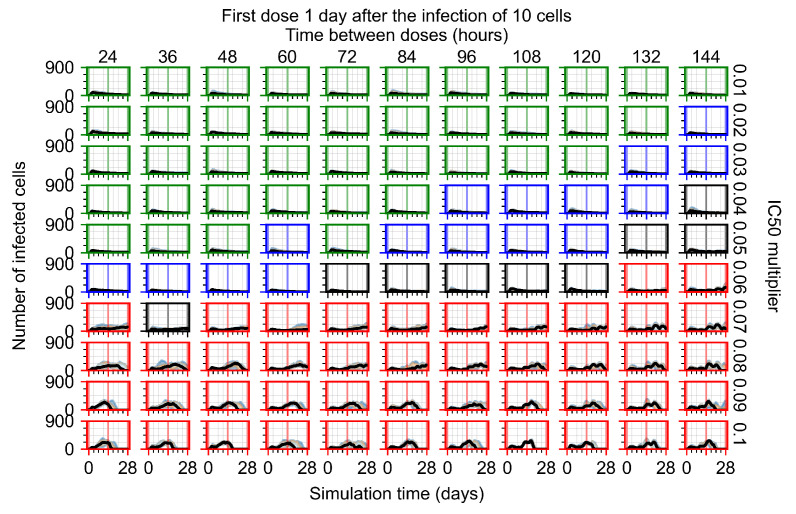
Infected (eclipse phase) population.

**Figure A23:**
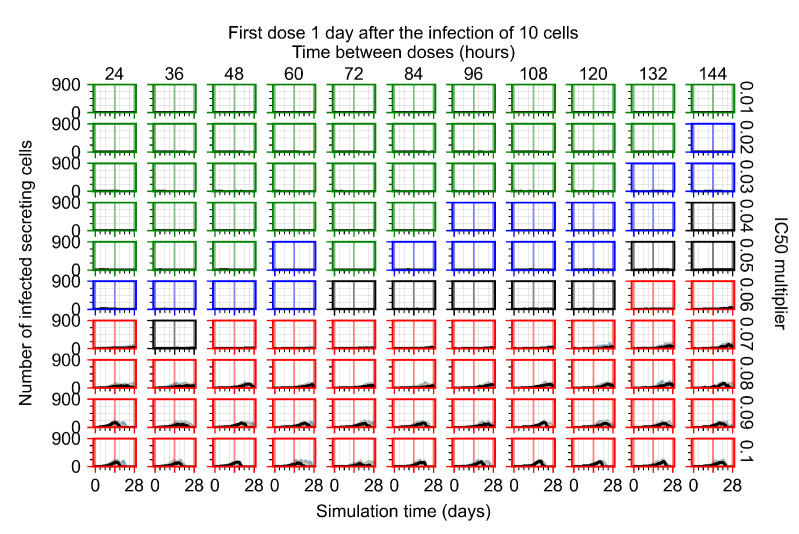
Infected (secreting extracellular virus) population.

**Figure A24:**
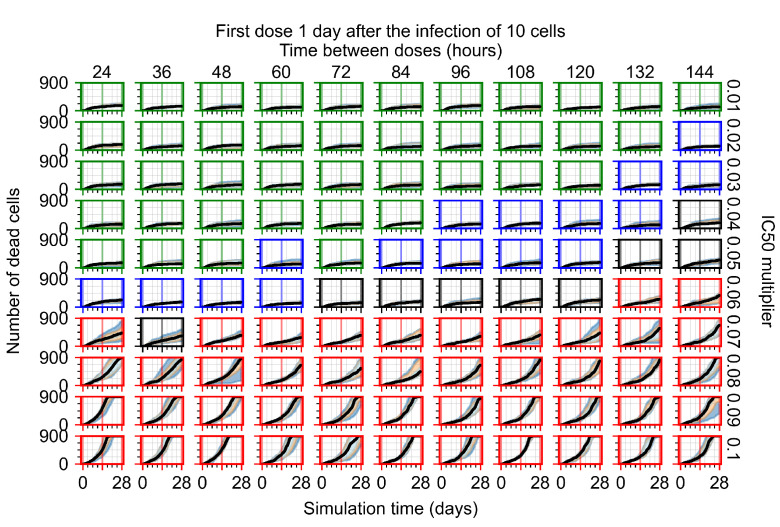
Dead population.

**Figure A25:**
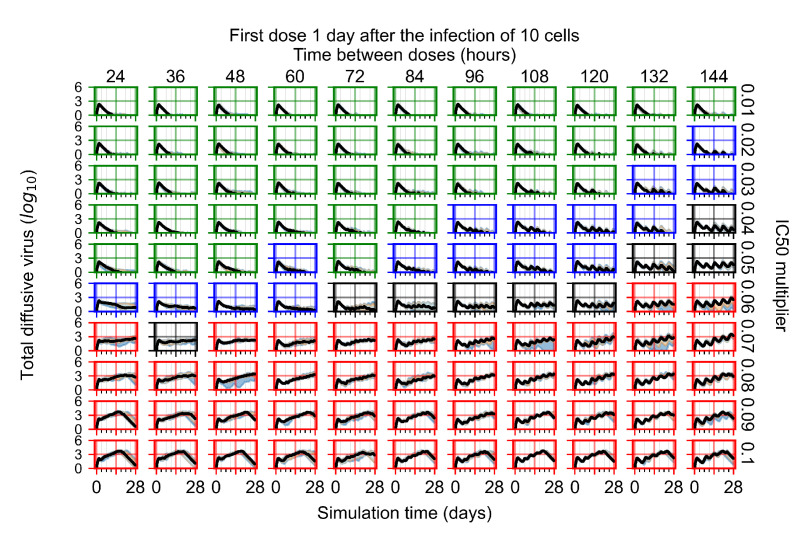
Extracellular diffusive virus quantities (arbitrary units), Y axis in log scale, and exponent values as tick marks.

**Figure A26:**
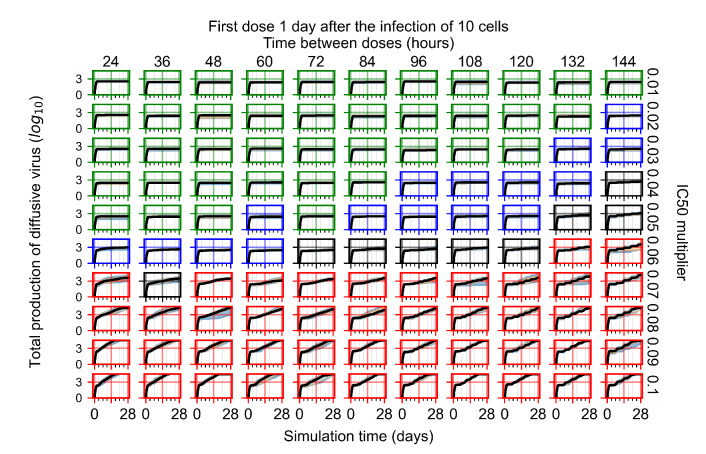
Total diffusive virus produced (AUC), Y axis in log scale, and exponent values as tick marks.

**Figure A27:**
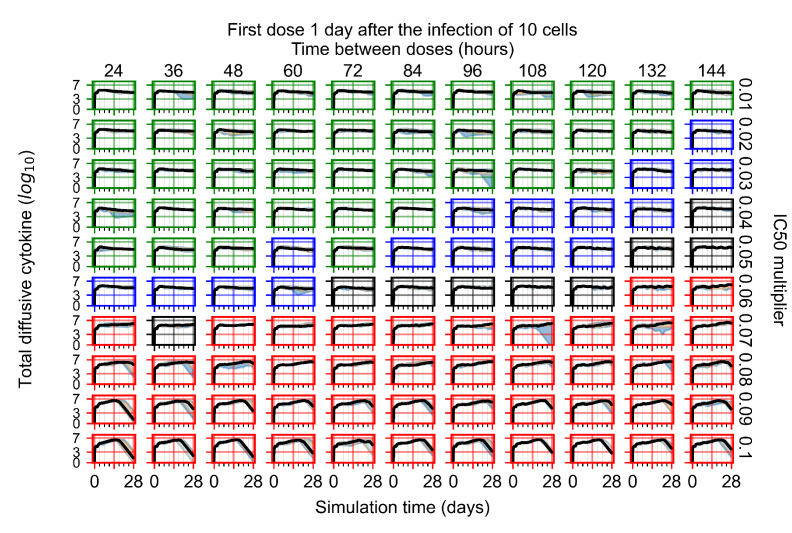
Diffusive cytokine amount, Y axis in log scale, and exponent values as tick marks.

**Figure A28:**
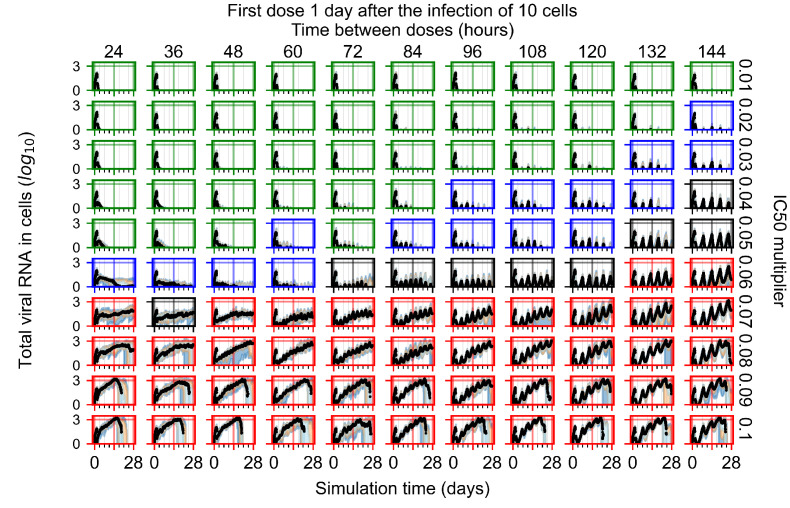
Amount of viral RNA in infected cells (arbitrary units), Y axis in log scale, and exponent values as tick marks.

**Figure A29:**
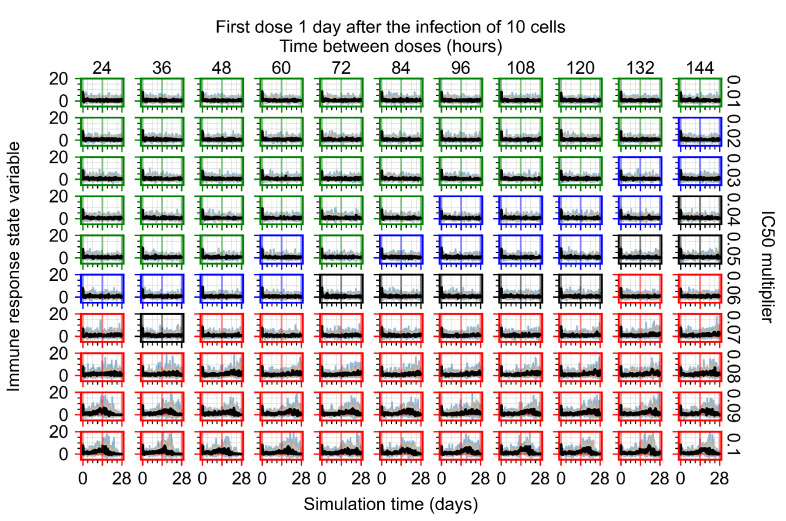
Immune response state variable number. Positive values correspond to an inflammatory state, and negative to an anti-inflammatory state.

**Figure A30:**
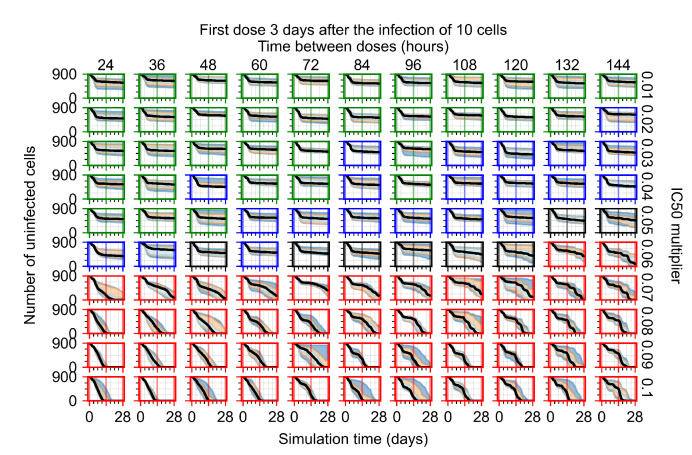
Uninfected population.

**Figure A31:**
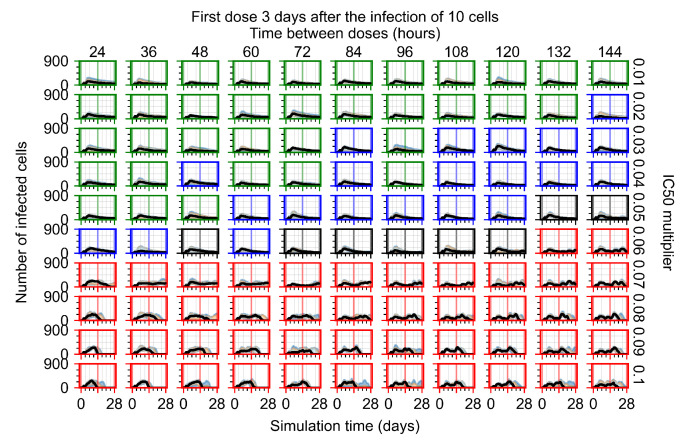
Infected (eclipse phase) population.

**Figure A32:**
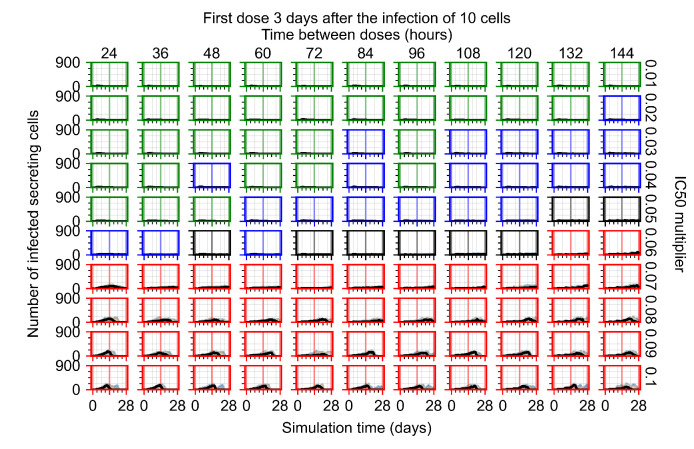
Infected (secreting extracellular virus) population.

**Figure A33:**
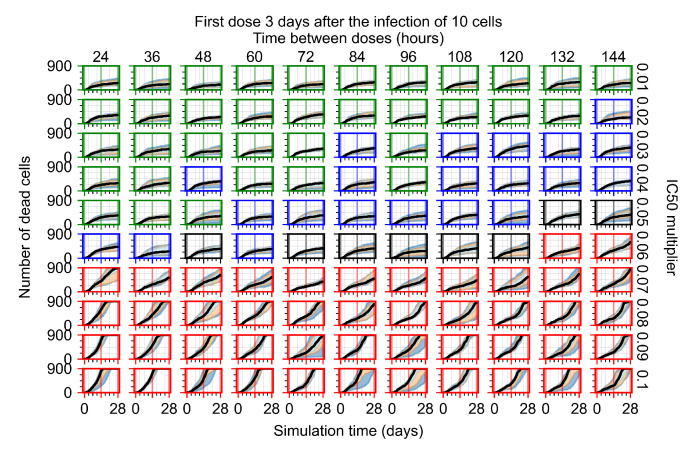
Dead population.

**Figure A34:**
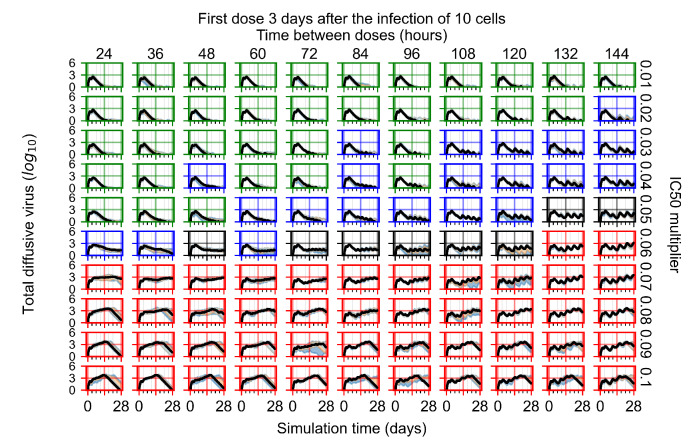
Extracellular diffusive virus quantities (arbitrary units), Y axis in log scale, and exponent values as tick marks.

**Figure A35:**
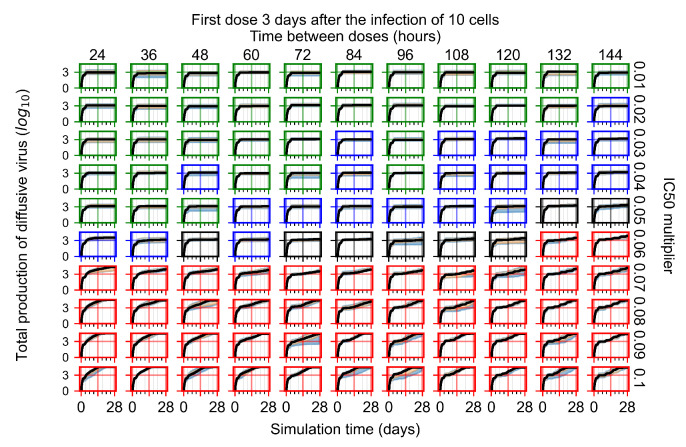
Total diffusive virus produced (AUC), Y axis in log scale, and exponent values as tick marks.

**Figure A36:**
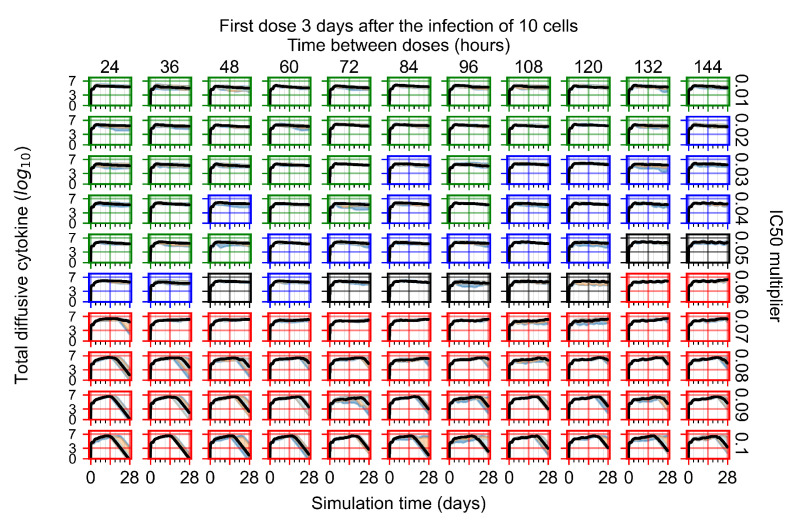
Diffusive cytokine amount, Y axis in log scale, and exponent values as tick marks.

**Figure A37:**
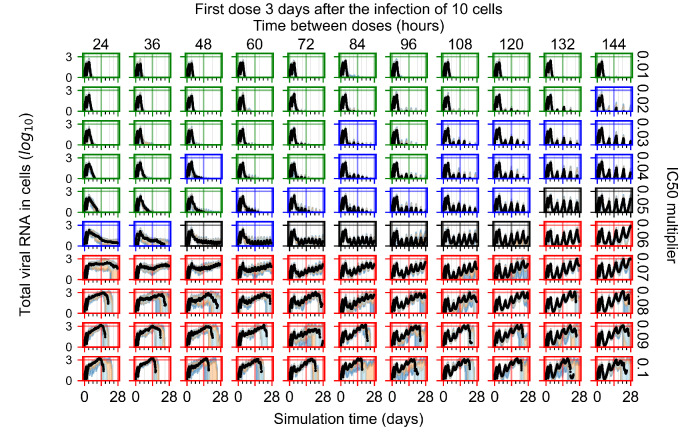
Amount of viral RNA in infected cells (arbitrary units), Y axis in log scale, and exponent values as tick marks.

**Figure A38:**
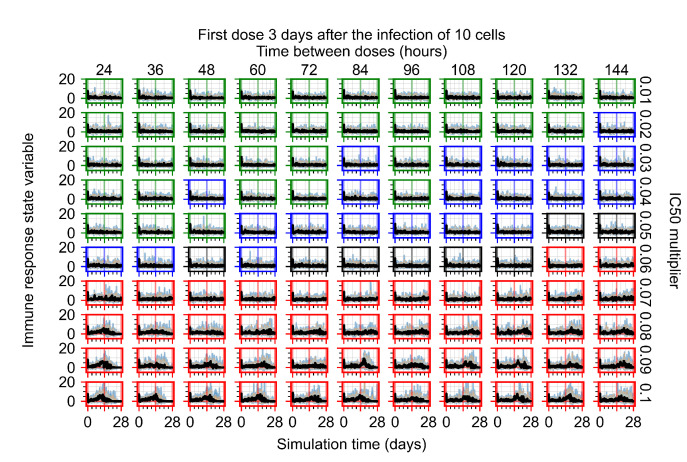
Immune response state variable number. Positive values correspond to an inflammatory state, and negative to an anti-inflammatory state.

**Figure A39:**
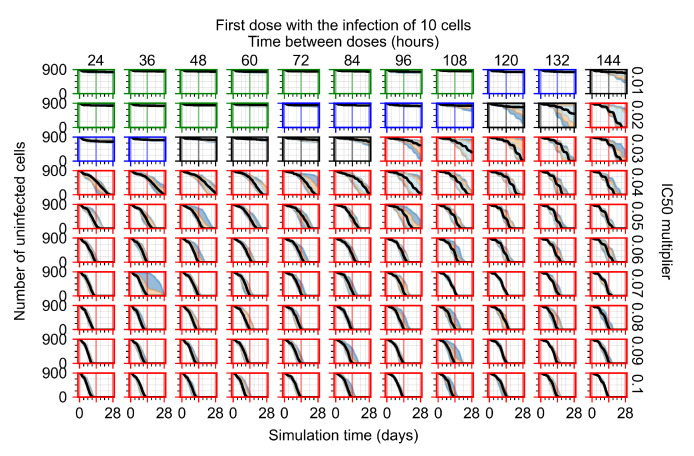
Uninfected population.

**Figure A40:**
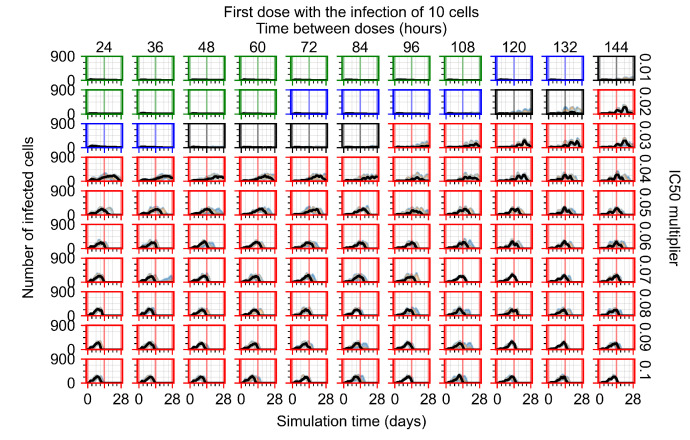
Infected (eclipse phase) population.

**Figure A41:**
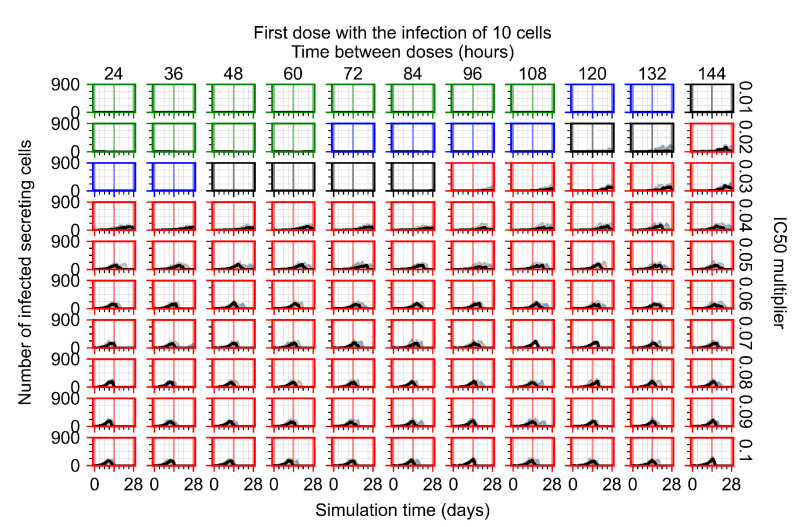
Infected (secreting extracellular virus) population.

**Figure A42:**
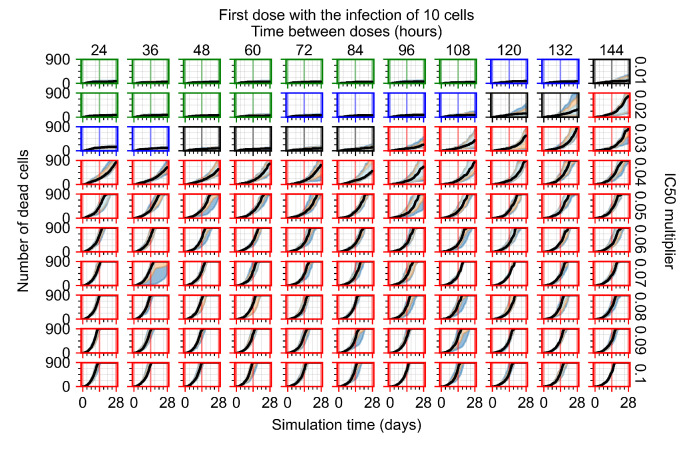
Dead population.

**Figure A43:**
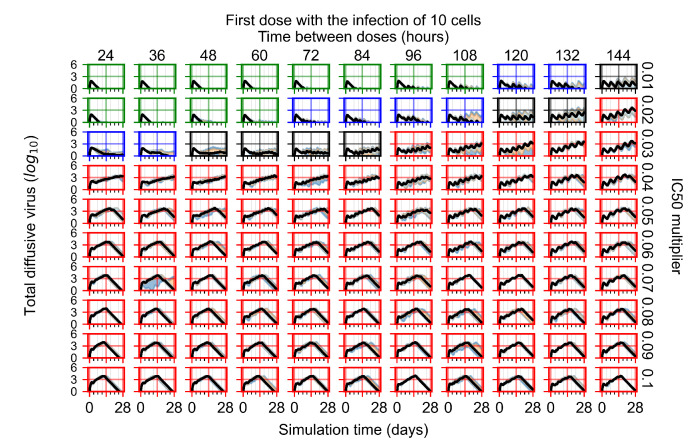
Extracellular diffusive virus quantities (arbitrary units), Y axis in log scale, and exponent values as tick marks.

**Figure A44:**
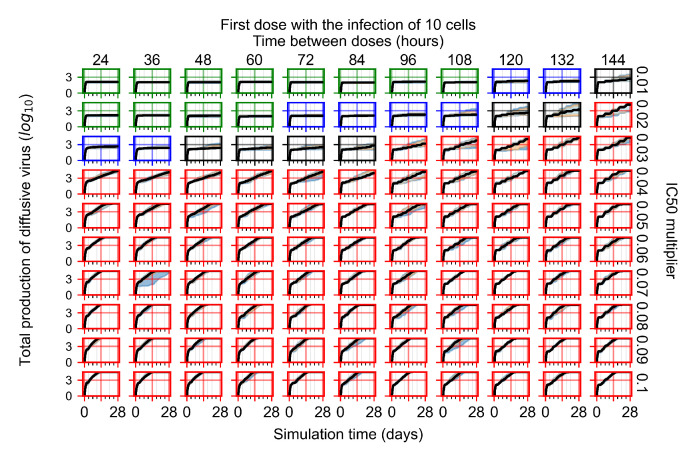
Total diffusive virus produced (AUC), Y axis in log scale, and exponent values as tick marks.

**Figure A45:**
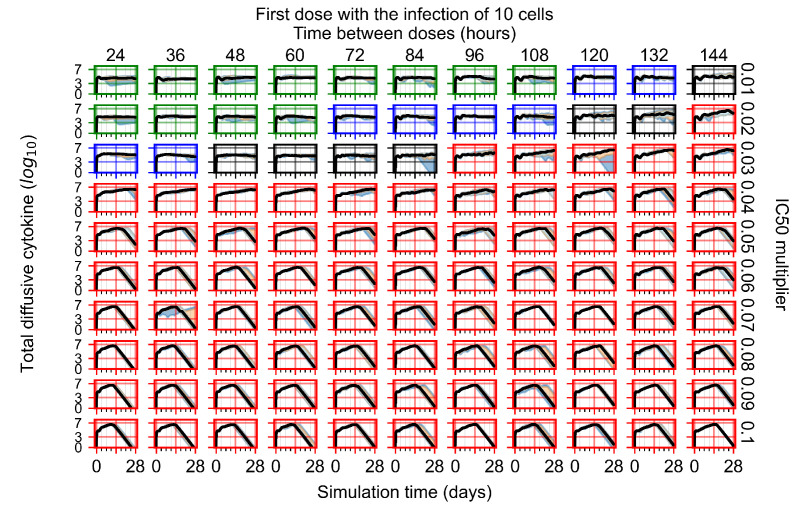
Diffusive cytokine amount, Y axis in log scale, and exponent values as tick marks.

**Figure A46:**
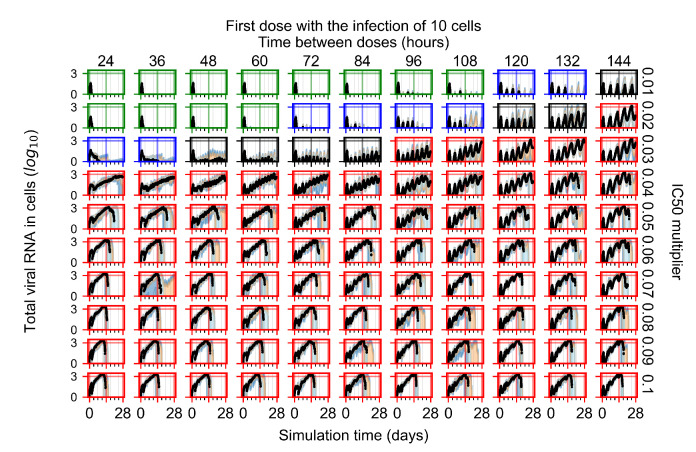
Amount of viral RNA in infected cells (arbitrary units), Y axis in log scale, and exponent values as tick marks.

**Figure A47:**
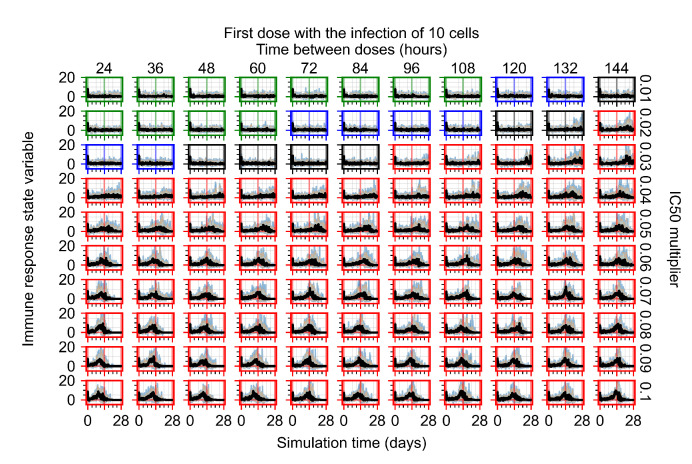
Immune response state variable number. Positive values correspond to an inflammatory state, and negative to an anti-inflammatory state.

**Figure A48:**
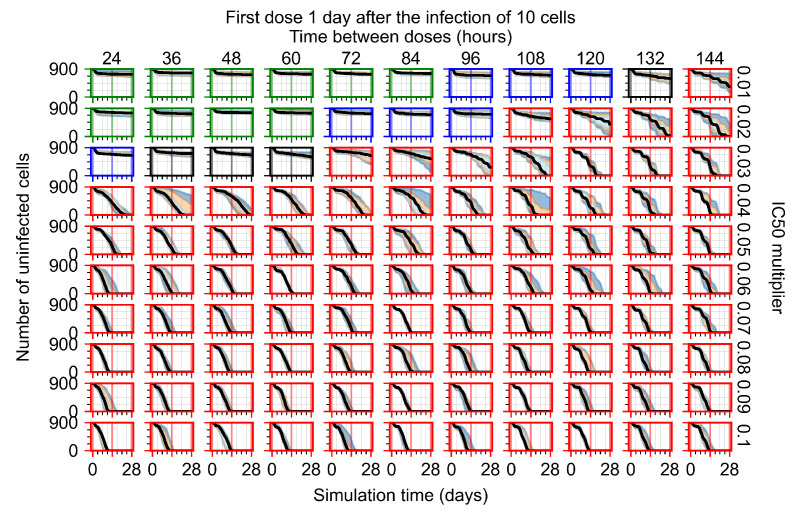
Uninfected population.

**Figure A49:**
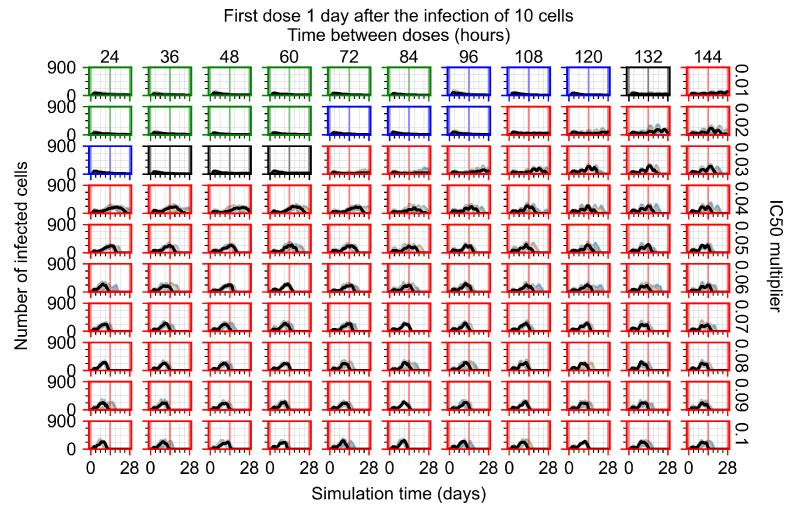
Infected (eclipse phase) population.

**Figure A50:**
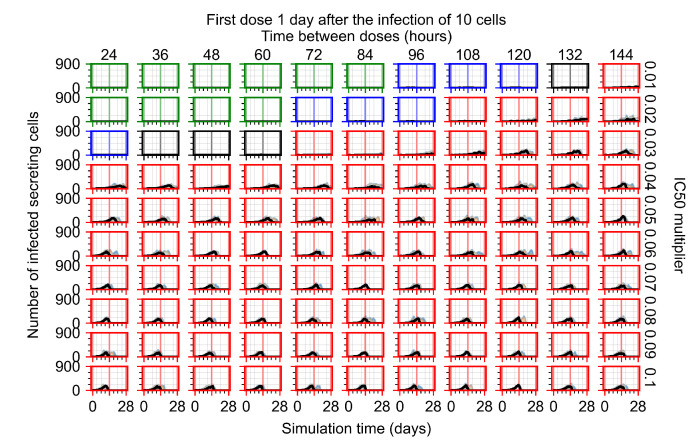
Infected (secreting extracellular virus).

**Figure A51:**
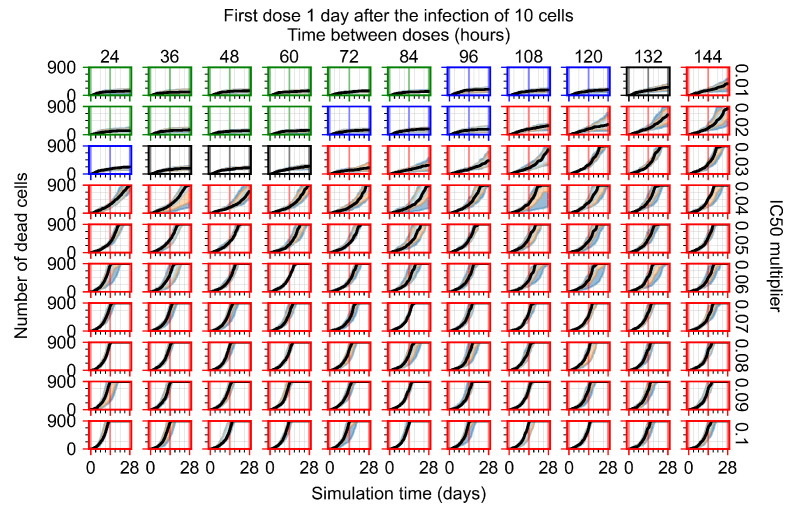
Dead population.

**Figure A52:**
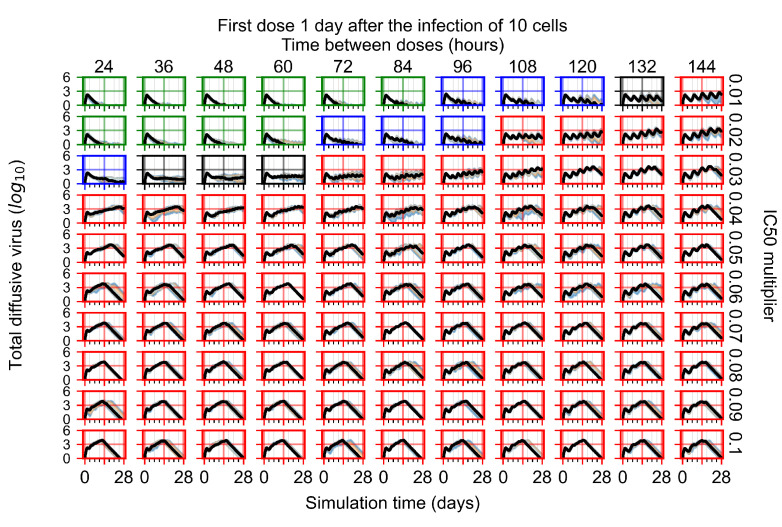
Extracellular diffusive virus quantities (arbitrary units), Y axis in log scale, and exponent values as tick marks.

**Figure A53:**
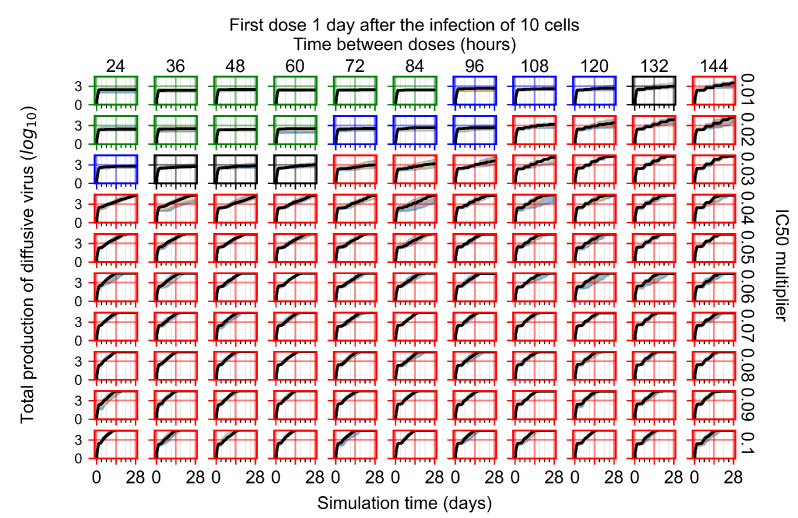
Total diffusive virus produced (AUC), Y axis in log scale, and exponent values as tick marks.

**Figure A54:**
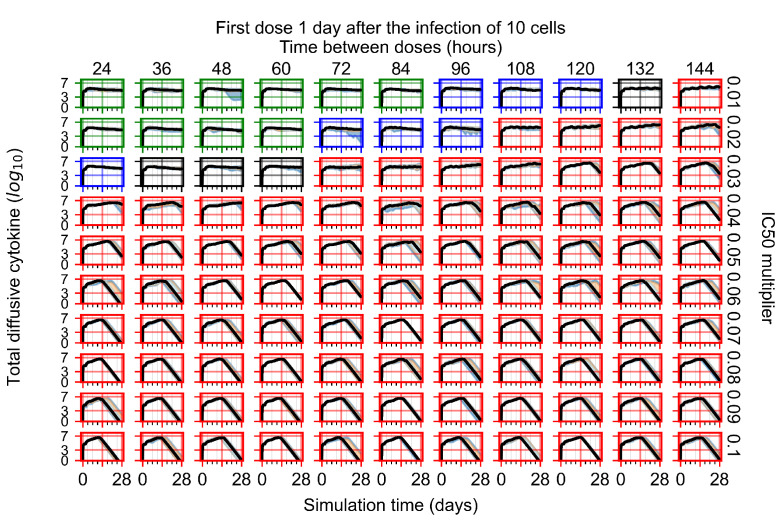
Diffusive cytokine amount, Y axis in log scale, and exponent values as tick marks.

**Figure A55:**
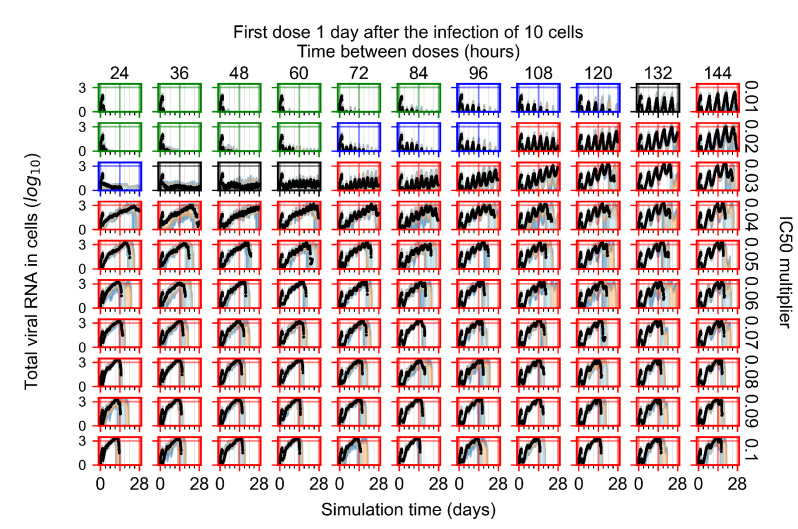
Amount of viral RNA in infected cells (arbitrary units), Y axis in log scale, and exponent values as tick marks.

**Figure A56:**
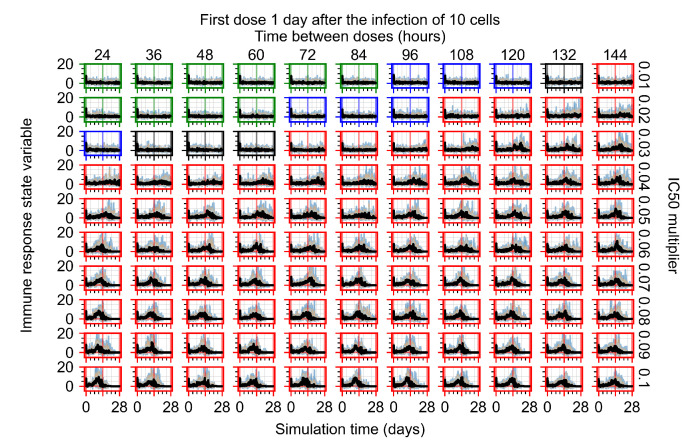
Immune response state variable number. Positive values correspond to an inflammatory state, and negative to an anti-inflammatory state.

**Figure A57:**
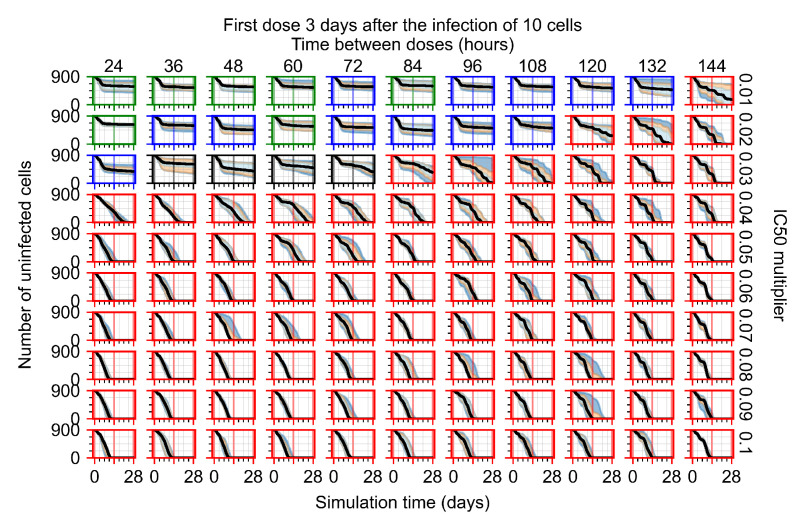
Uninfected population.

**Figure A58:**
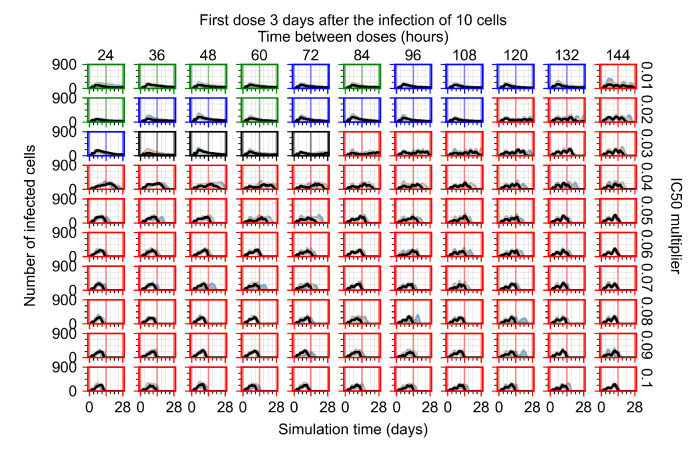
Infected (eclipse phase) population.

**Figure A59:**
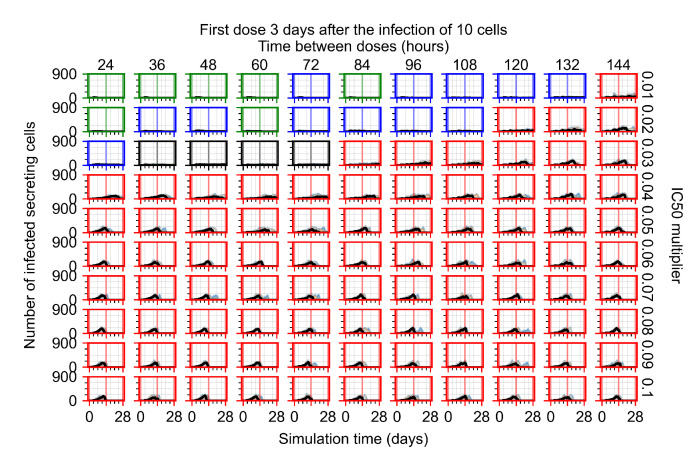
Infected (secreting extracellular virus) population.

**Figure A60:**
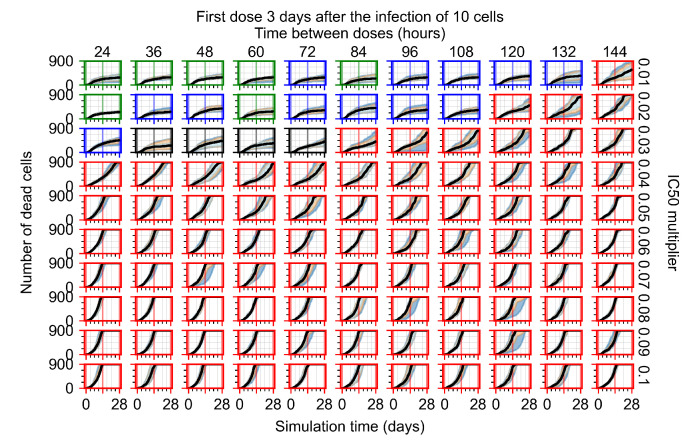
Dead population.

**Figure A61:**
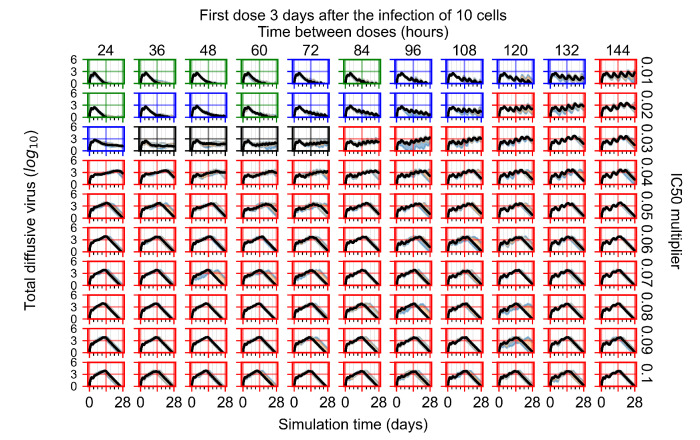
Extracellular diffusive virus quantities (arbitrary units), Y axis in log scale, and exponent values as tick marks.

**Figure A62:**
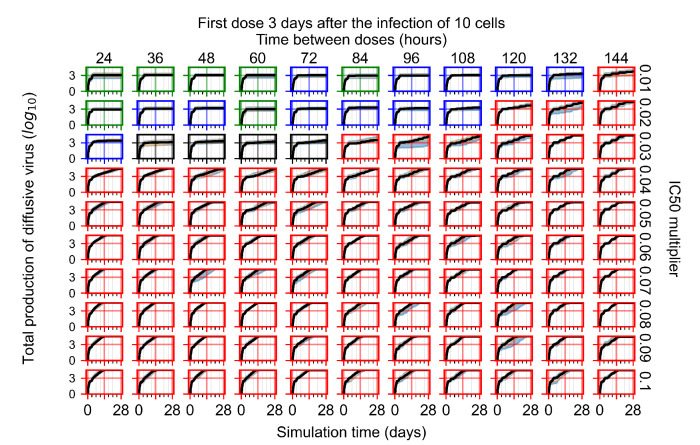
Total diffusive virus produced (AUC), Y axis in log scale, and exponent values as tick marks.

**Figure A63:**
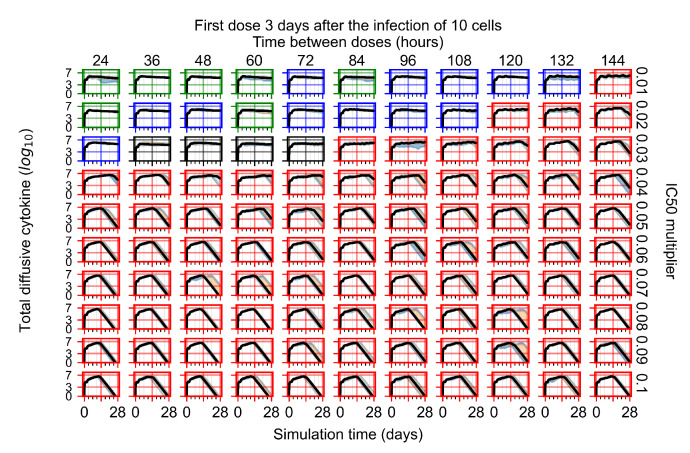
Diffusive cytokine amount, Y axis in log scale, and exponent values as tick marks.

**Figure A64:**
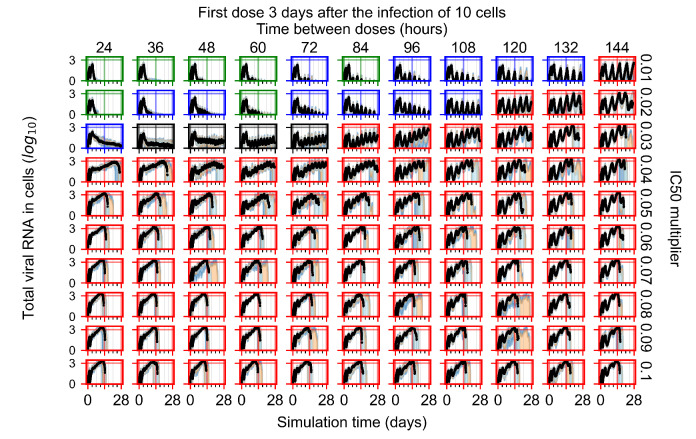
Amount of viral RNA in infected cells (arbitrary units), Y axis in log scale, and exponent values as tick marks.

**Figure A65:**
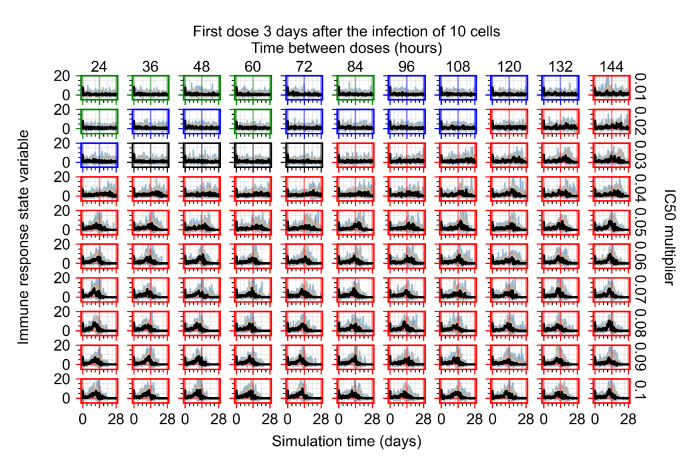
Immune response state variable number. Positive values correspond to an inflammatory state, and negative to an anti-inflammatory state.

**Figure A66:**
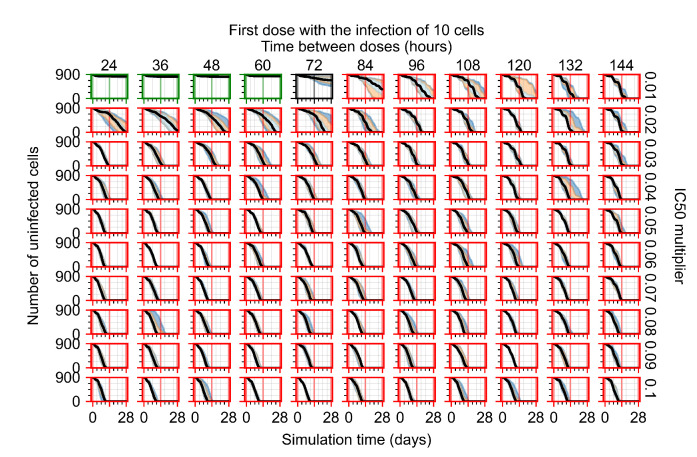
Uninfected population.

**Figure A67:**
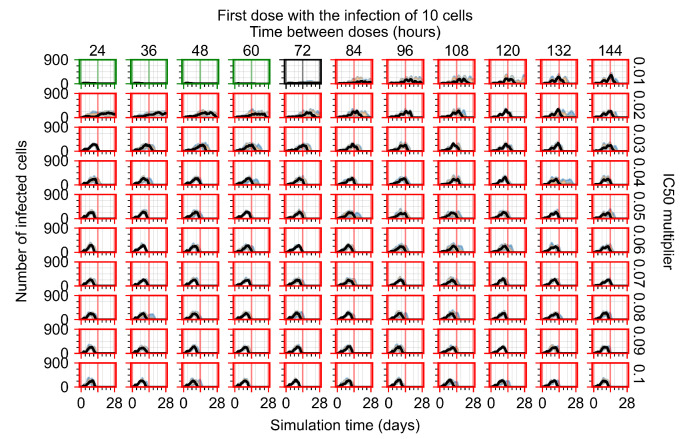
Infected (eclipse phase) population.

**Figure A68:**
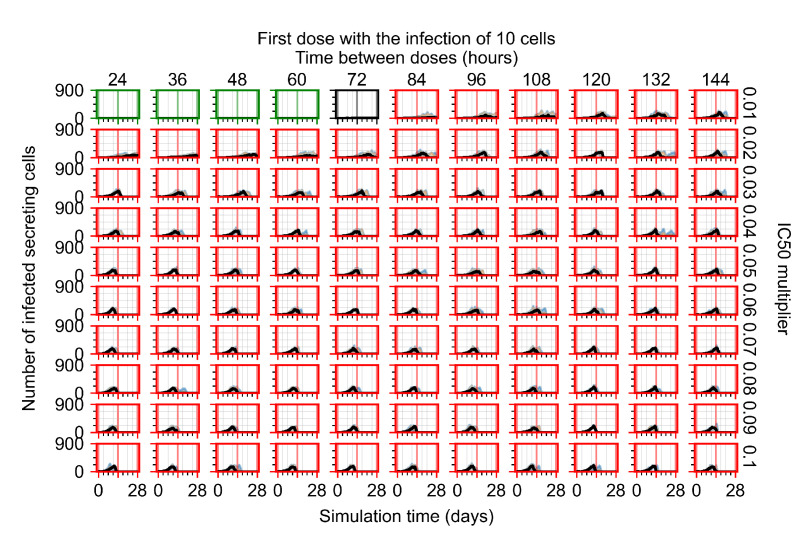
Infected (secreting extracellular virus) population.

**Figure A69:**
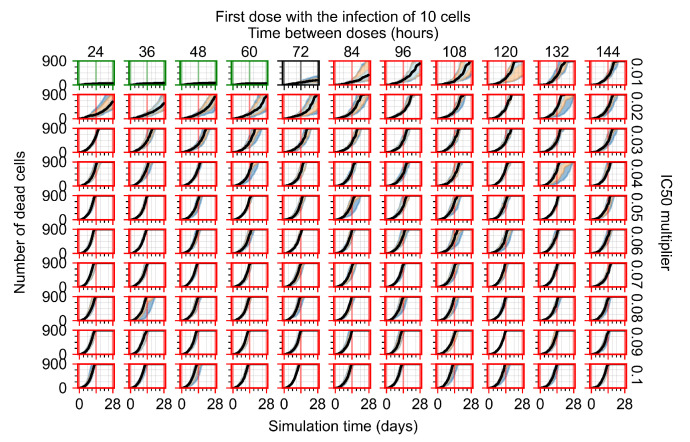
Dead population.

**Figure A70:**
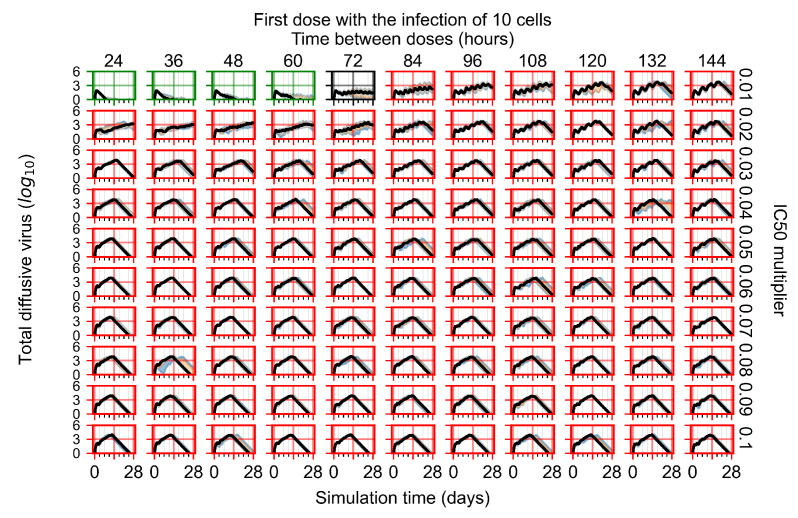
Extracellular diffusive virus quantities (arbitrary units), Y axis in log scale, and exponent values as tick marks.

**Figure A71:**
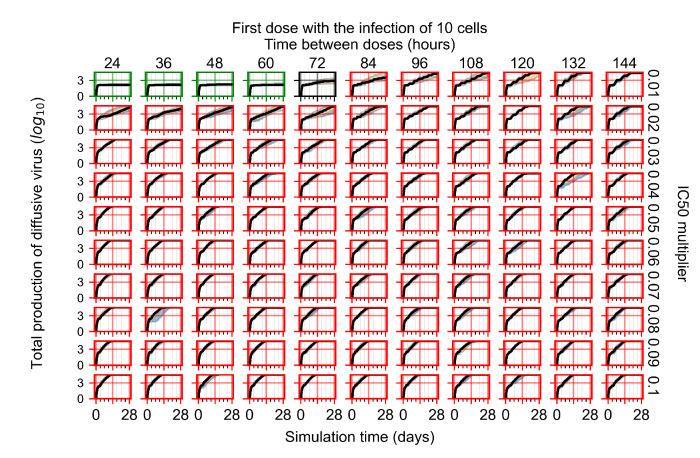
Total diffusive virus produced (AUC), Y axis in log scale, and exponent values as tick marks.

**Figure A72:**
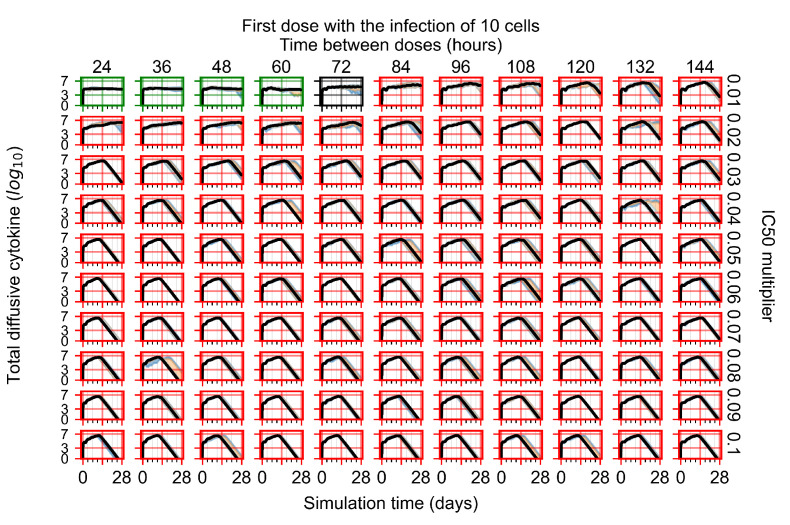
Diffusive cytokine amount, Y axis in log scale, and exponent values as tick marks.

**Figure A73:**
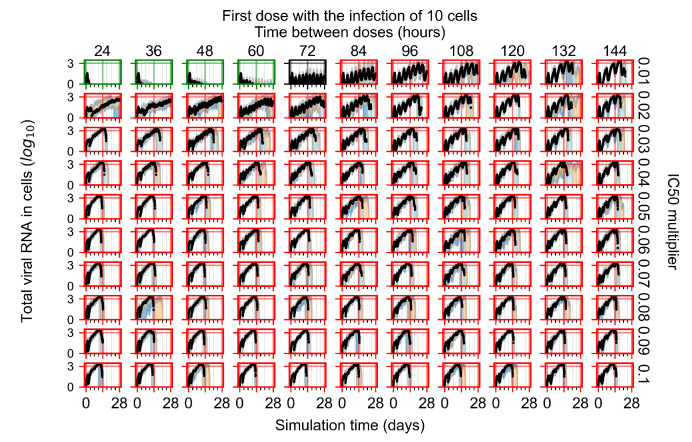
Amount of viral RNA in infected cells (arbitrary units), Y axis in log scale, and exponent values as tick marks.

**Figure A74:**
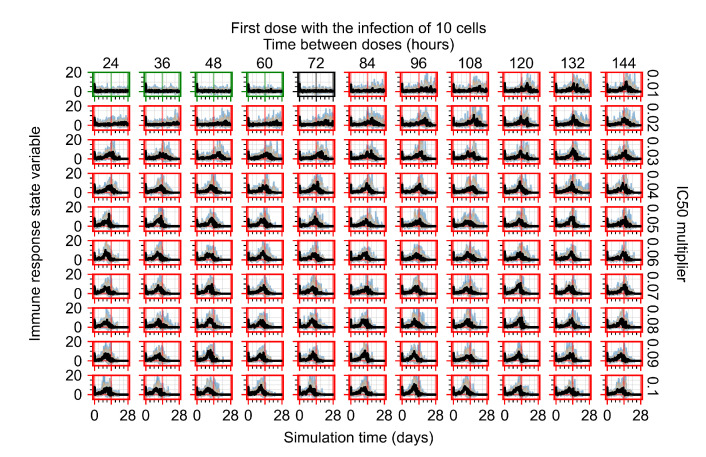
Immune response state variable number. Positive values correspond to an inflammatory state, and negative to an anti-inflammatory state.

**Figure A75:**
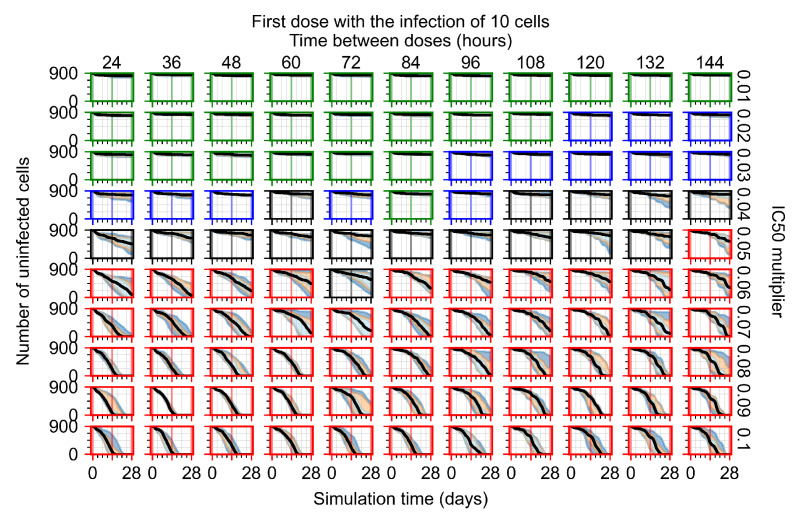
Uninfected population.

**Figure A76:**
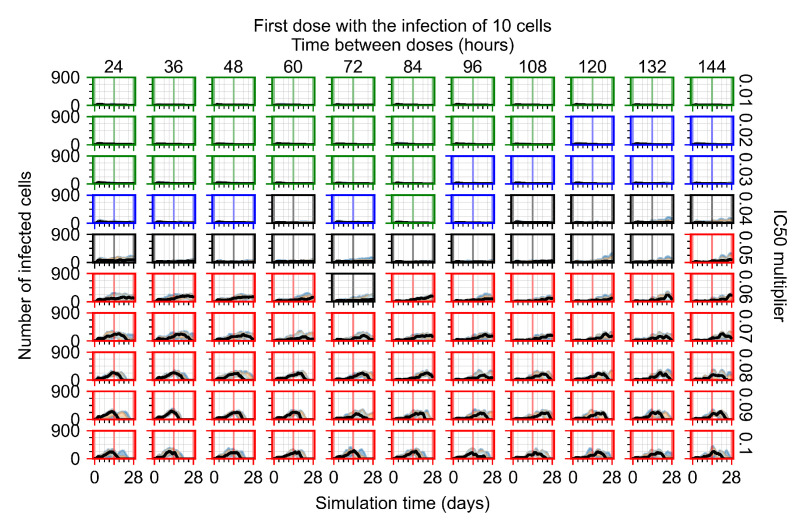
Infected (eclipse phase) population.

**Figure A77:**
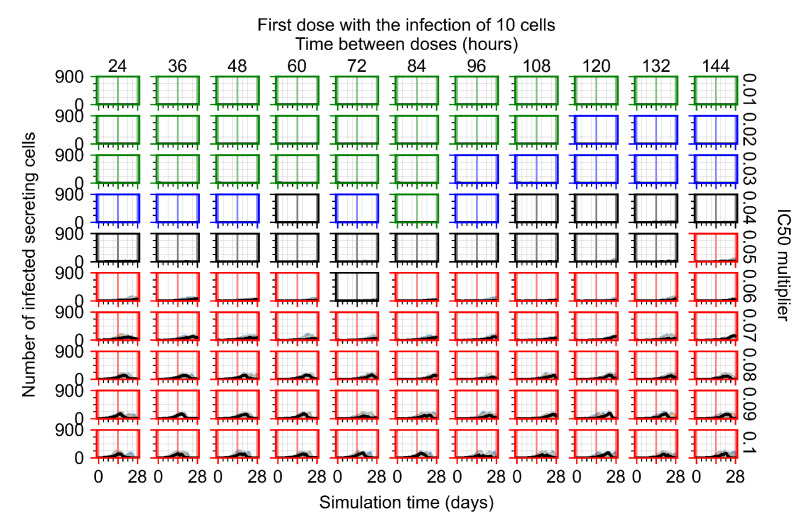
Infected (secreting extracellular virus) population.

**Figure A78:**
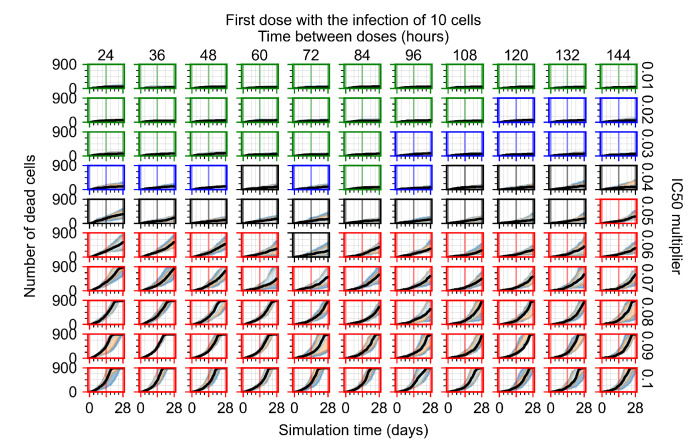
Dead population.

**Figure A79:**
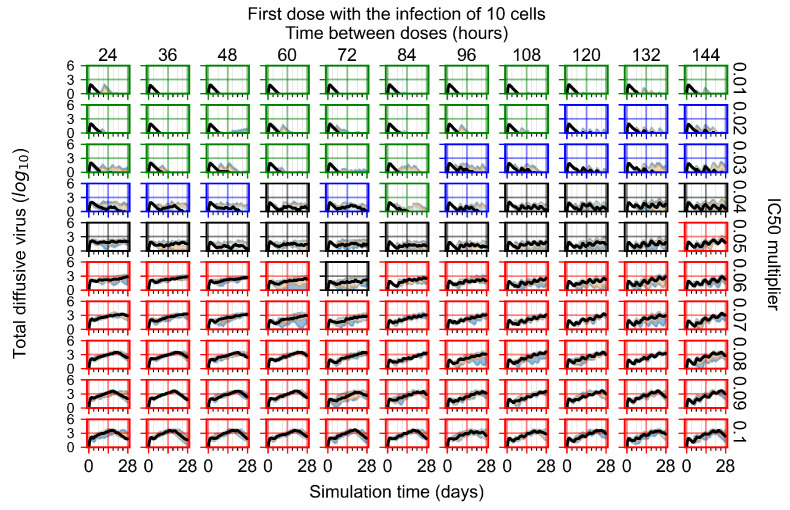
Extracellular diffusive virus quantities (arbitrary units), Y axis in log scale, and exponent values as tick marks.

**Figure A80:**
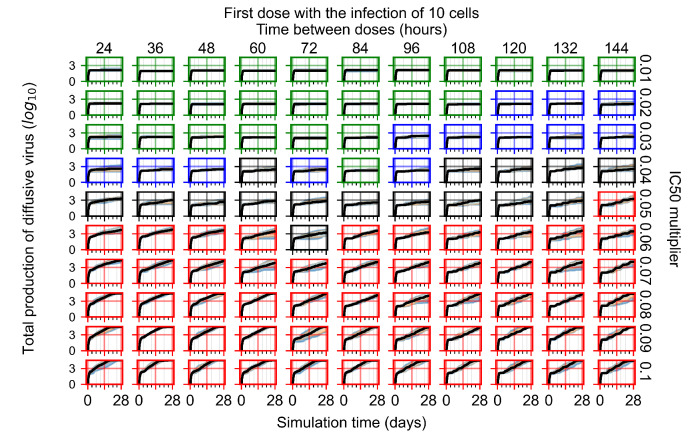
Total diffusive virus produced (AUC) for eight replicas of the treatment simulation, Y axis in log scale, and exponent values as tick marks.

**Figure A81:**
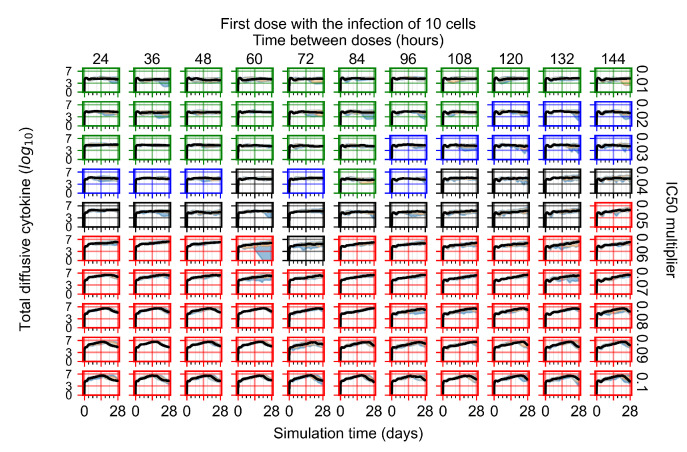
Diffusive cytokine amount for eight replicas of the treatment simulation, Y axis in log scale, and exponent values as tick marks.

**Figure A82:**
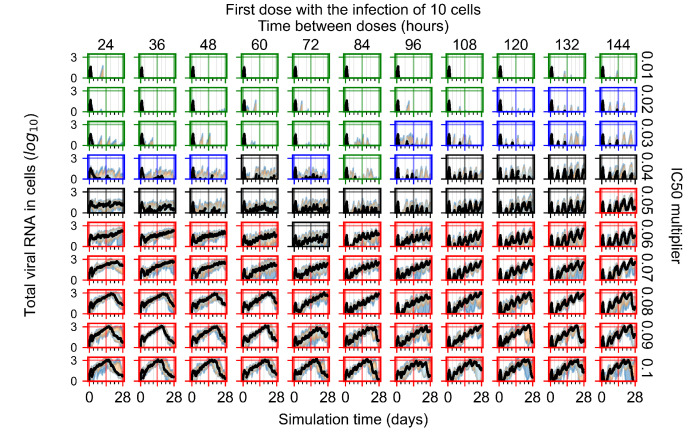
Amount of viral RNA in infected cells (arbitrary units), Y axis in log scale, and exponent values as tick marks.

**Figure A83:**
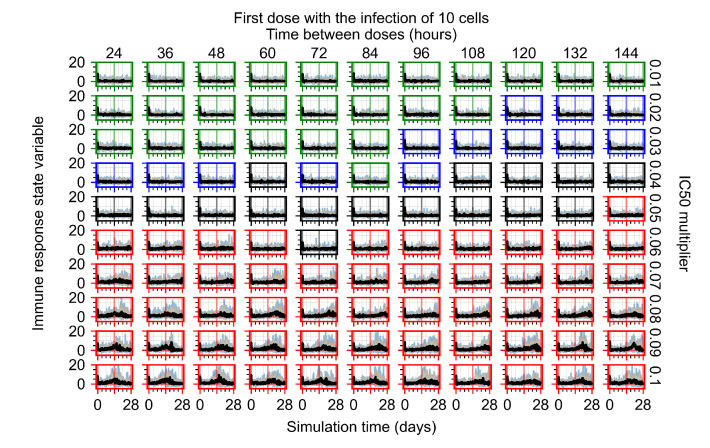
Immune response state variable number. Positive values correspond to an inflammatory state, and negative to an anti-inflammatory state.

**Figure A84:**
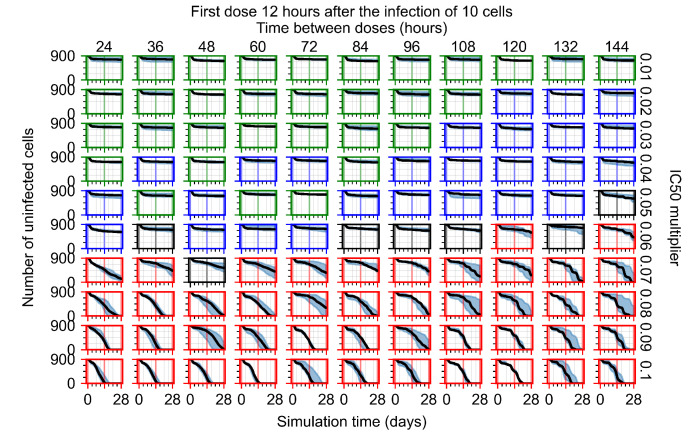
Uninfected population.

**Figure A85:**
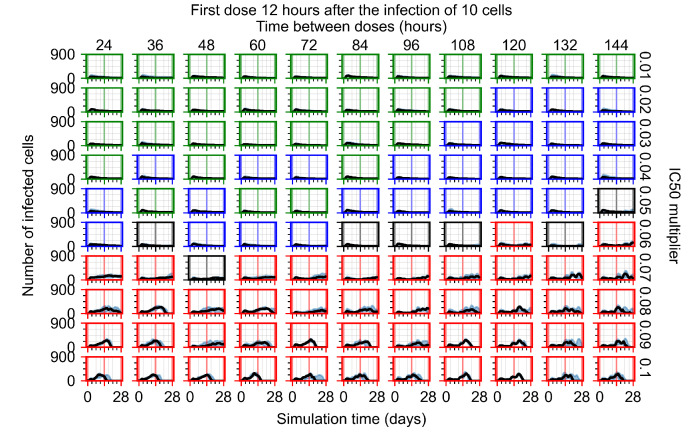
Infected (eclipse phase) population.

**Figure A86:**
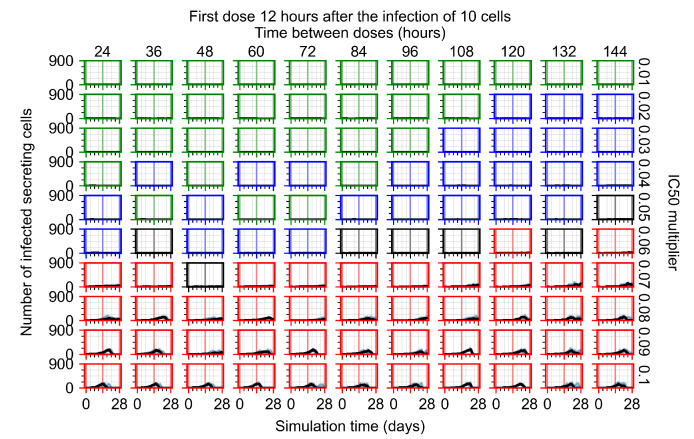
Infected (secreting extracellular virus) population.

**Figure A87:**
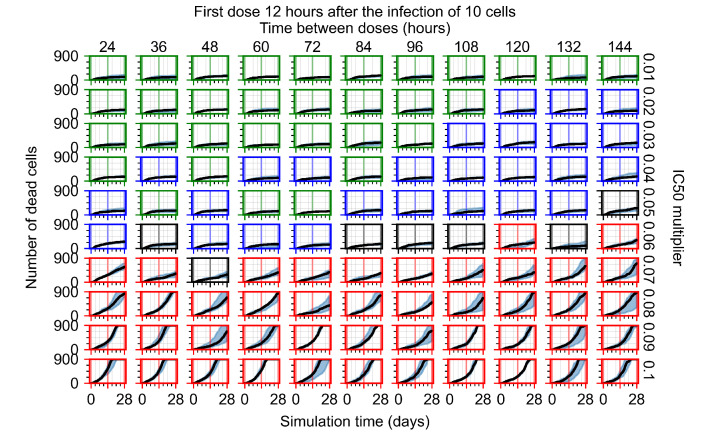
Dead population.

**Figure A88:**
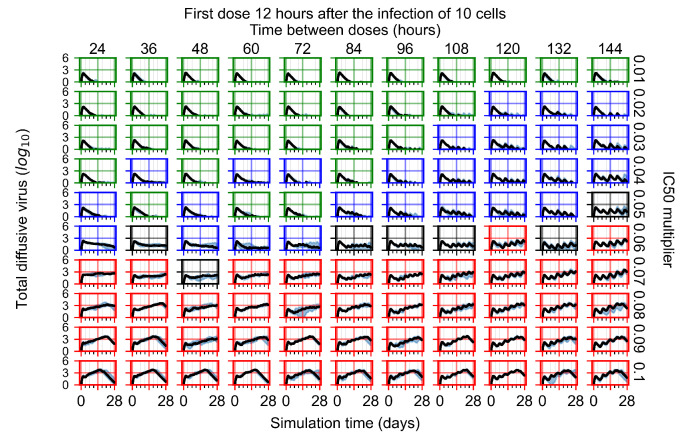
Extracellular diffusive virus quantities (arbitrary units), Y axis in log scale, and exponent values as tick marks.

**Figure A89:**
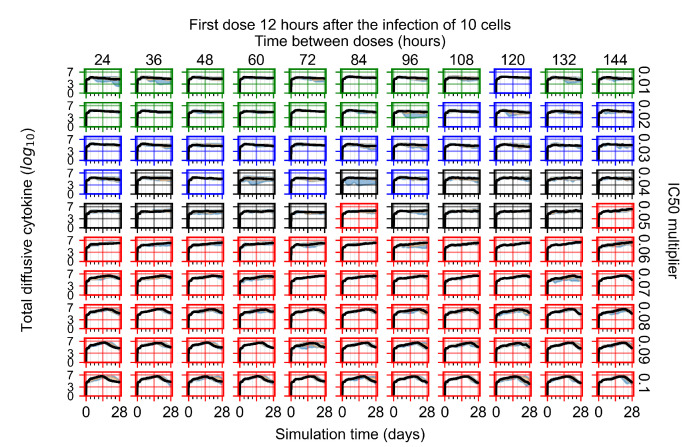
Diffusive cytokine amount for eight replicas of the treatment simulation, Y axis in log scale, and exponent values as tick marks.

**Figure A90:**
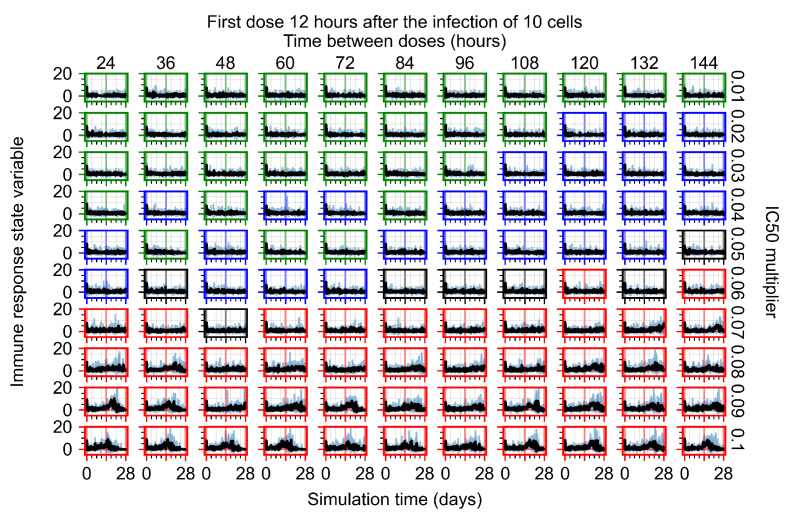
Immune response state variable number. Positive values correspond to an inflammatory state, and negative to an anti-inflammatory state.

**Figure A91:**
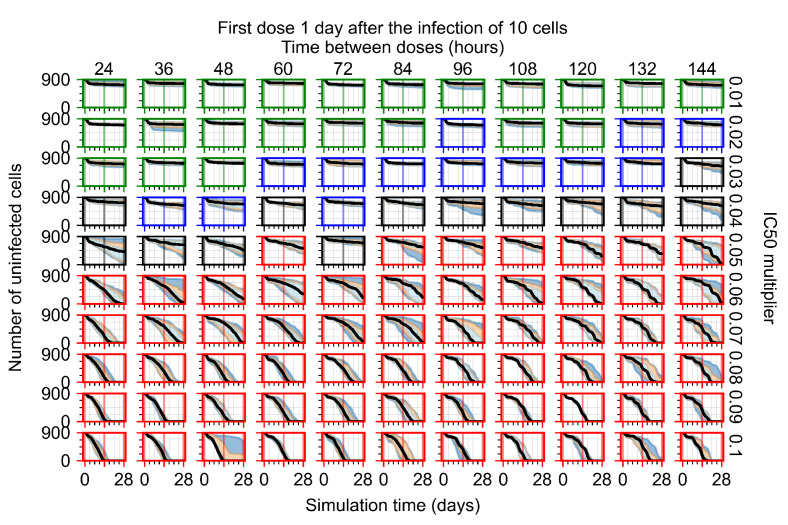
Uninfected population.

**Figure A92:**
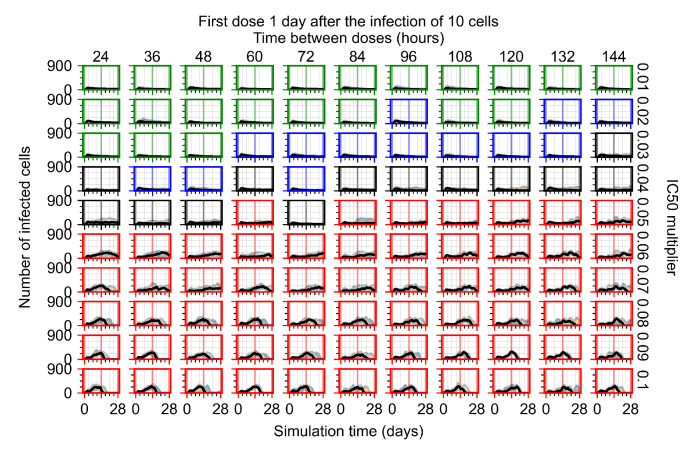
Infected (eclipse phase) population.

**Figure A93:**
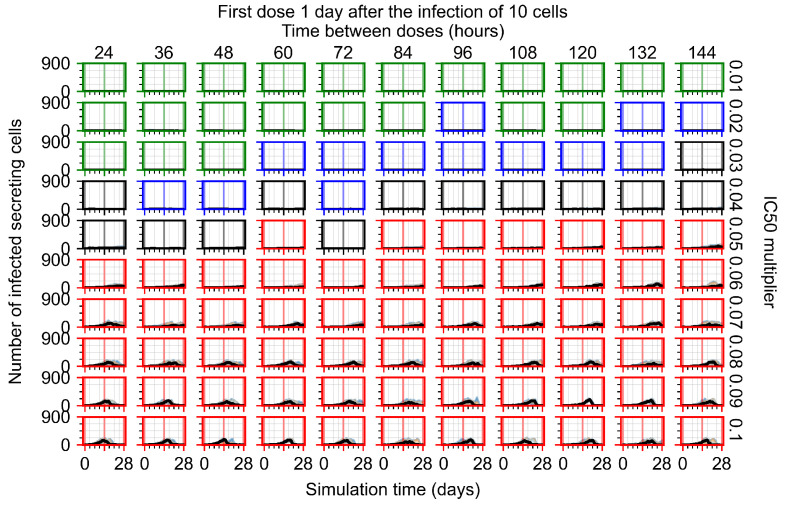
Infected (secreting extracellular virus) population.

**Figure A94:**
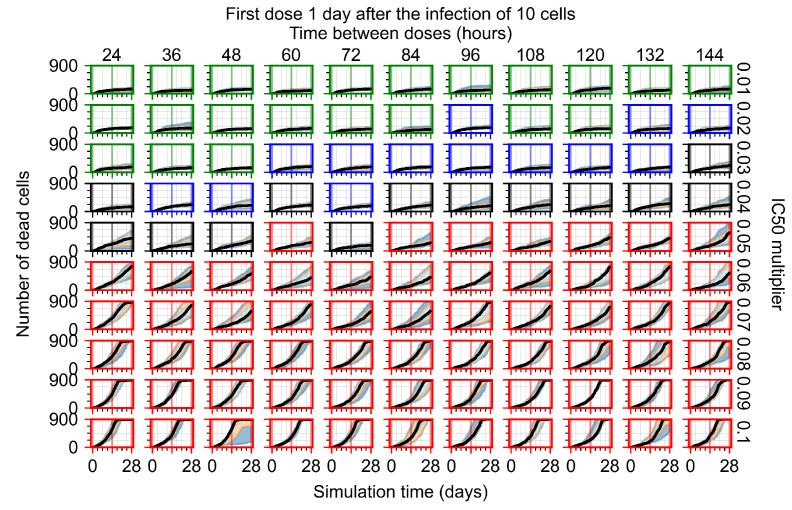
Dead population.

**Figure A95:**
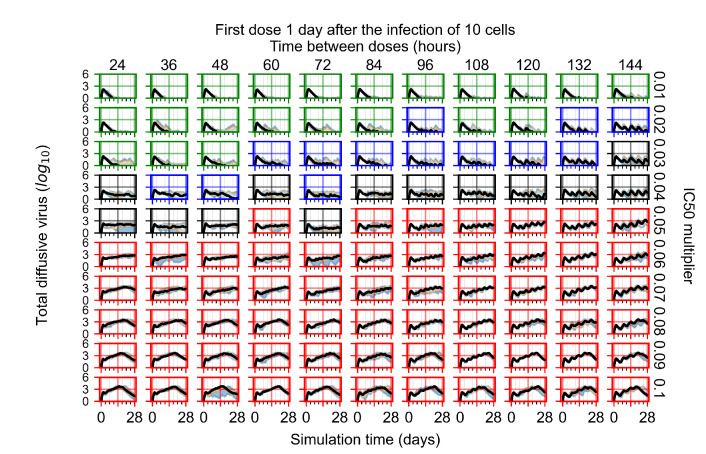
Extracellular diffusive virus quantities (arbitrary units), Y axis in log scale, and exponent values as tick marks.

**Figure A96:**
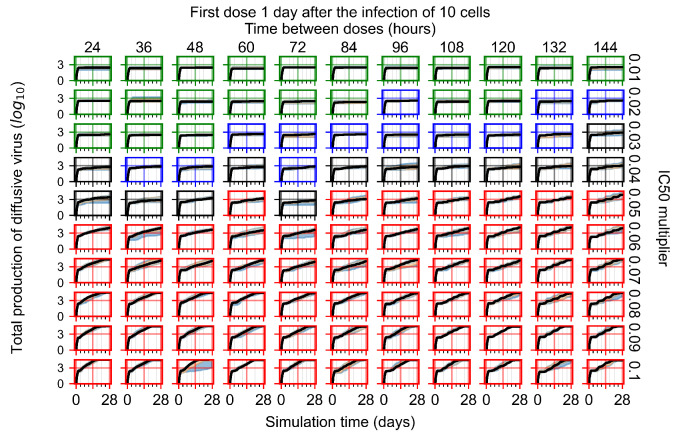
Total diffusive virus produced (AUC), Y axis in log scale, and exponent values as tick marks.

**Figure A97:**
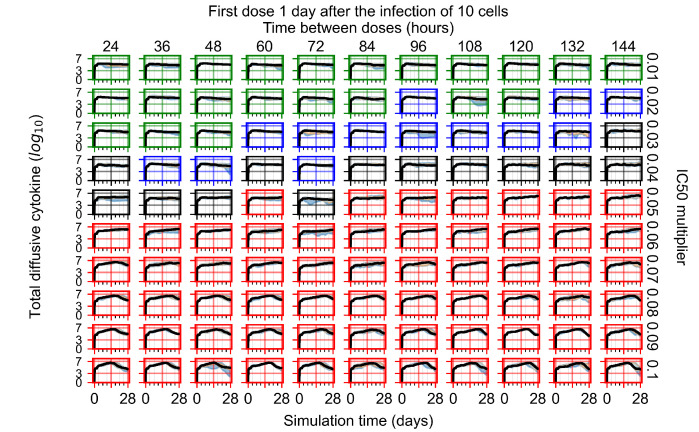
Diffusive cytokine amount for eight replicas of the treatment simulation, Y axis in log scale, and exponent values as tick marks.

**Figure A98:**
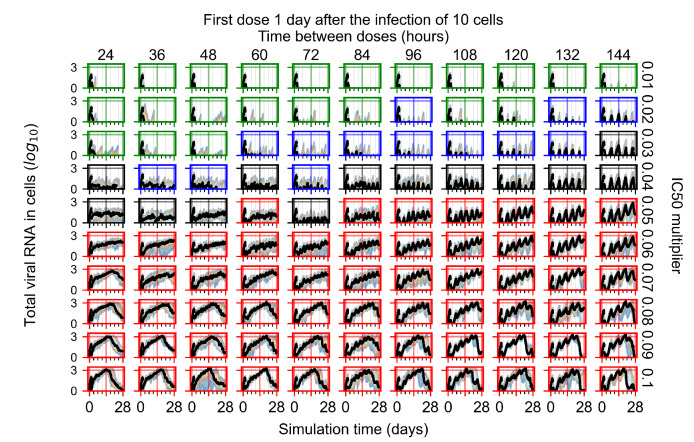
Amount of viral RNA in infected cells (arbitrary units), Y axis in log scale, and exponent values as tick marks.

**Figure A99:**
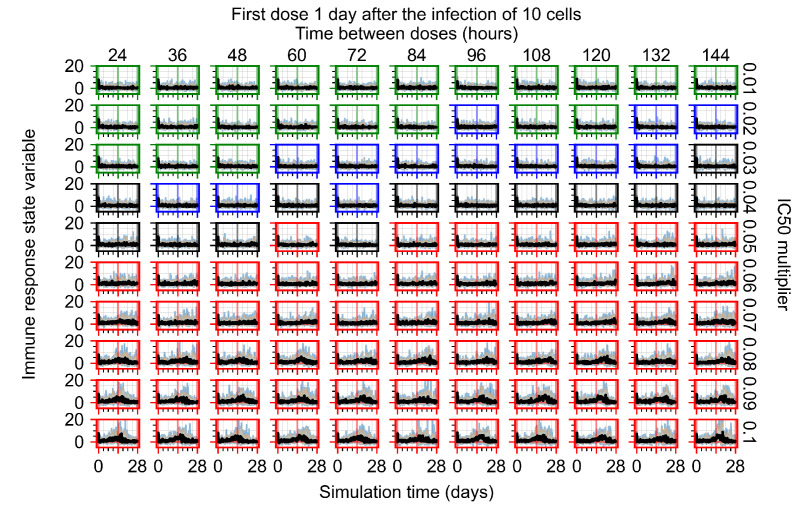
Immune response state variable number. Positive values correspond to an inflammatory state, and negative to an anti-inflammatory state.

**Figure A100:**
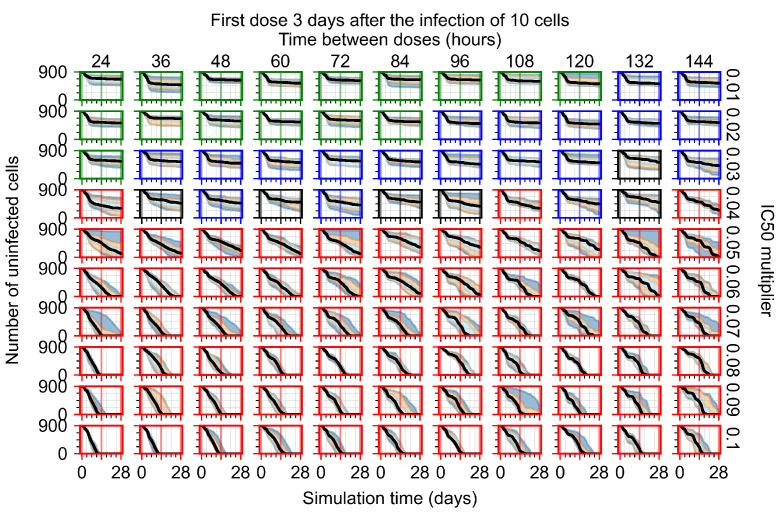
Uninfected population.

**Figure A101:**
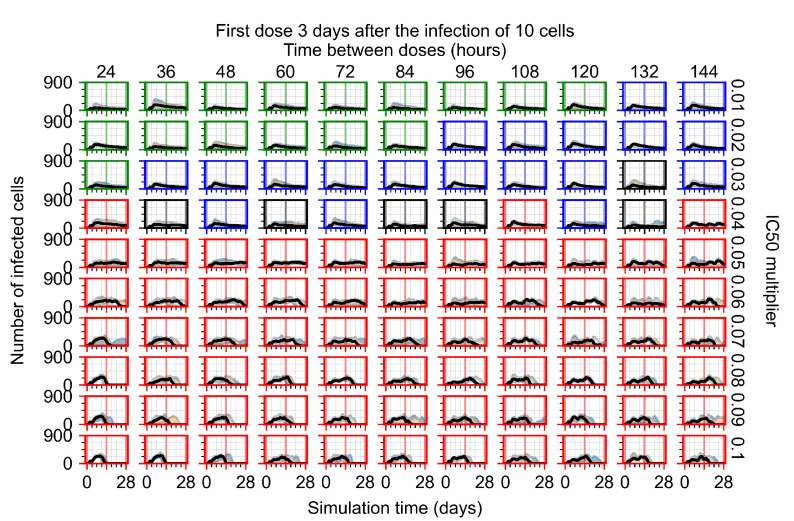
Infected (eclipse phase) population.

**Figure A102:**
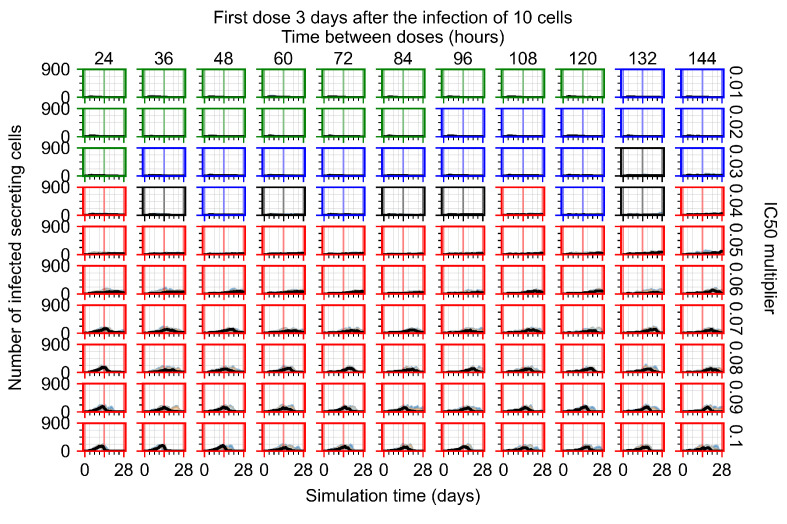
Infected (secreting extracellular virus) population.

**Figure A103:**
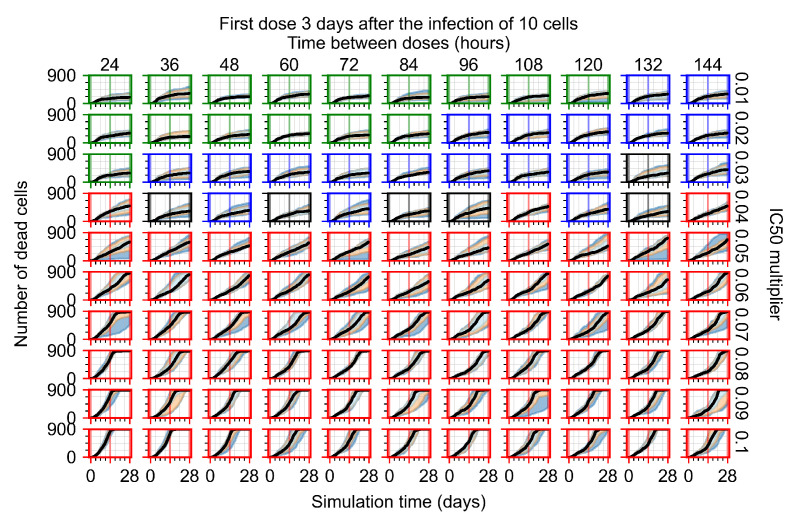
Dead population.

**Figure A104:**
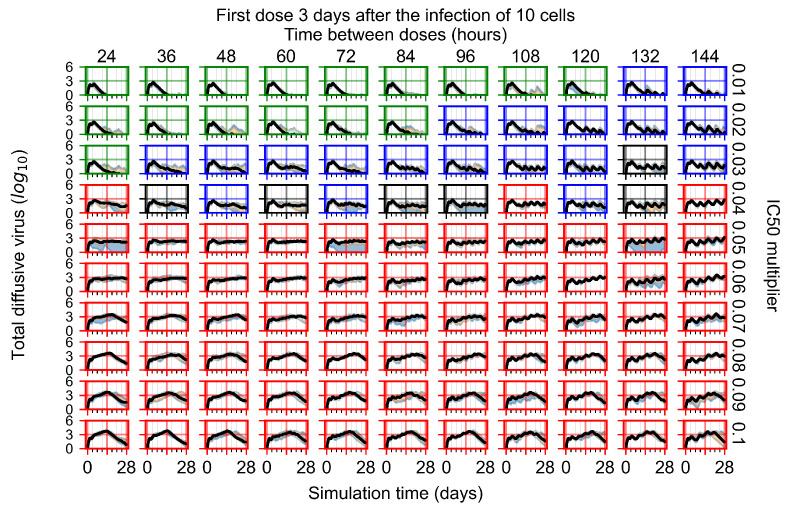
Extracellular diffusive virus quantities (arbitrary units), Y axis in log scale, and exponent values as tick marks.

**Figure A105:**
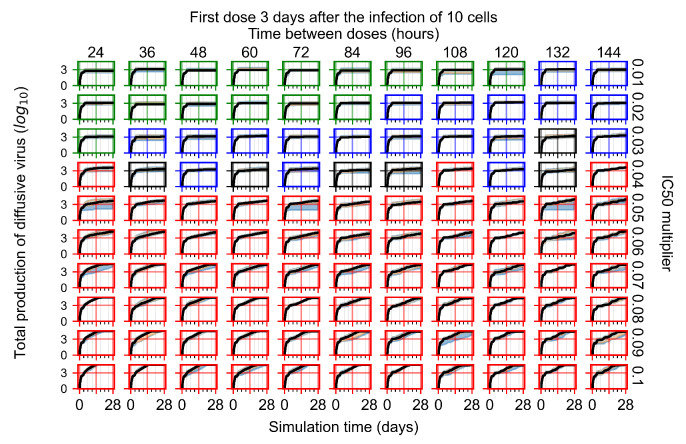
Total diffusive virus produced (AUC), Y axis in log scale, and exponent values as tick marks.

**Figure A106:**
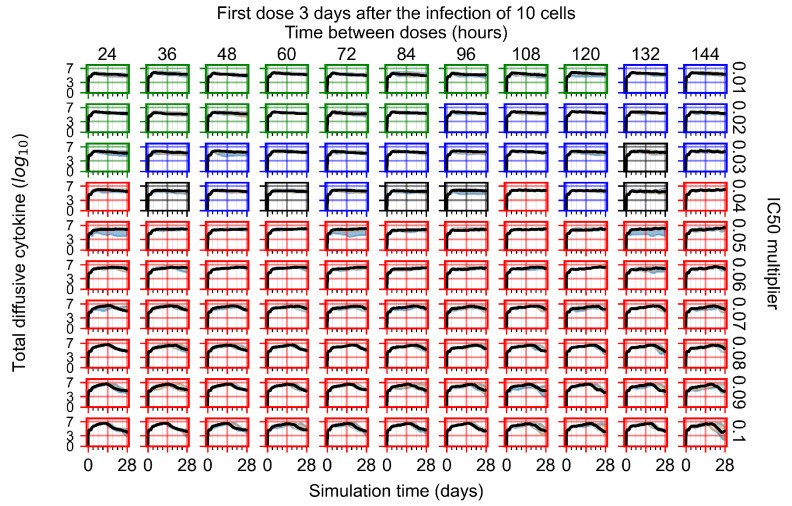
Diffusive cytokine amount, Y axis in log scale, and exponent values as tick marks.

**Figure A107:**
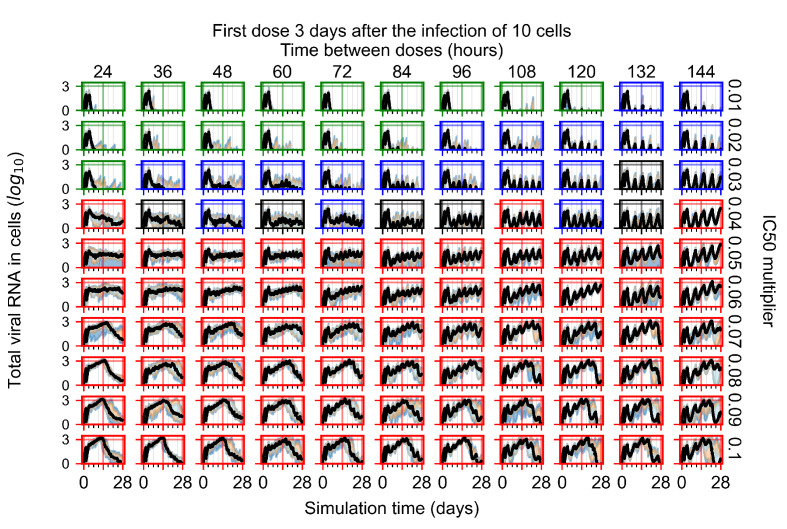
Amount of viral RNA in infected cells (arbitrary units), Y axis in log scale, and exponent values as tick marks.

**Figure A108:**
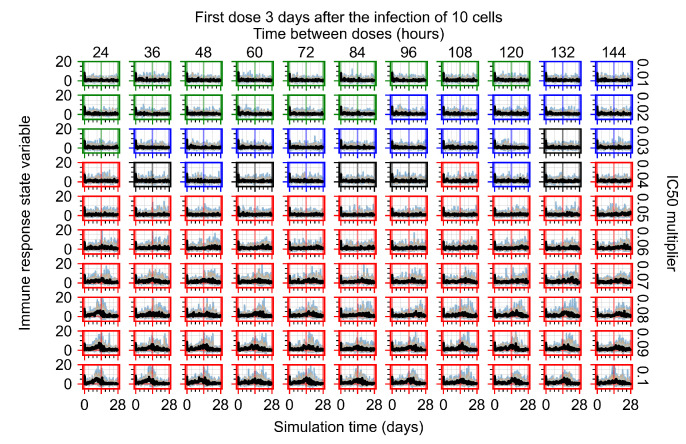
Immune response state variable number. Positive values correspond to an inflammatory state, and negative to an anti-inflammatory state.

**Figure A109:**
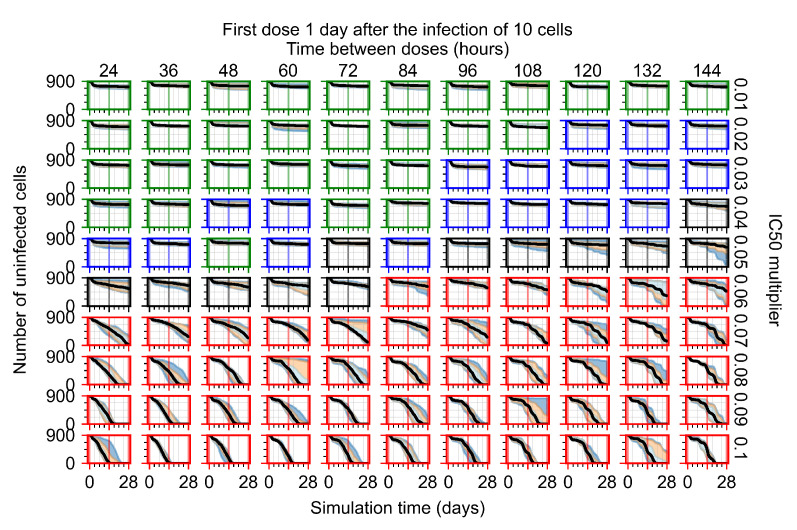
Uninfected population.

**Figure A110:**
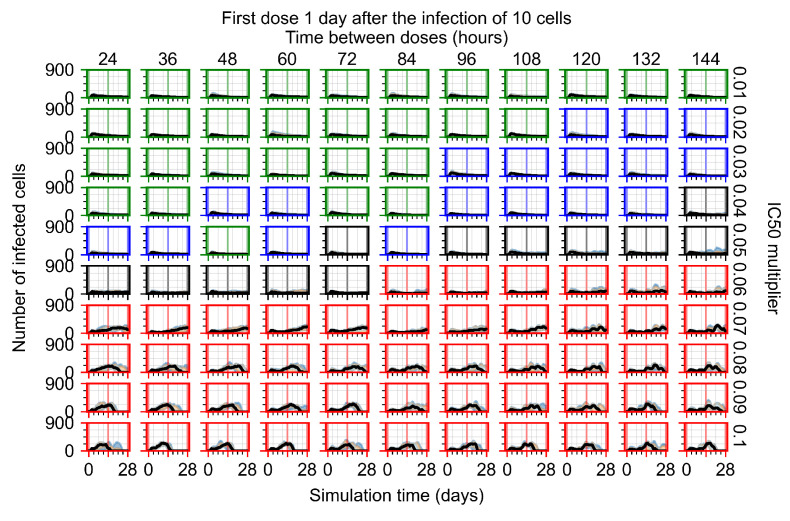
Infected (eclipse phase) population.

**Figure A111:**
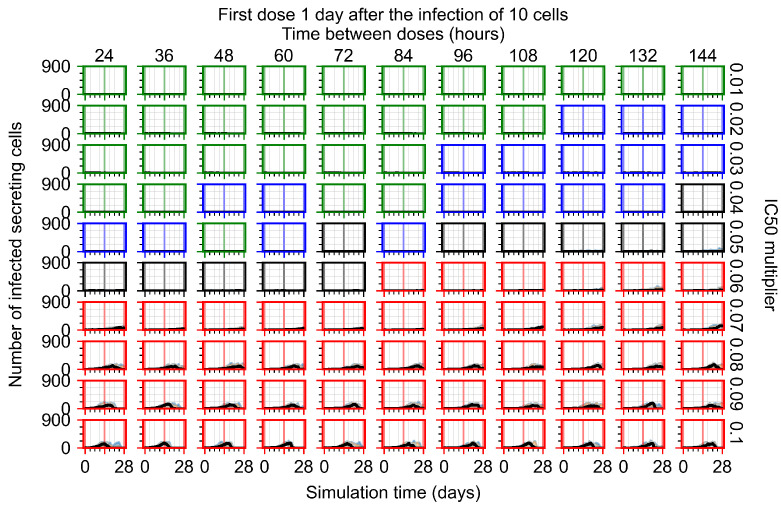
Infected (secreting extracellular virus) population.

**Figure A112:**
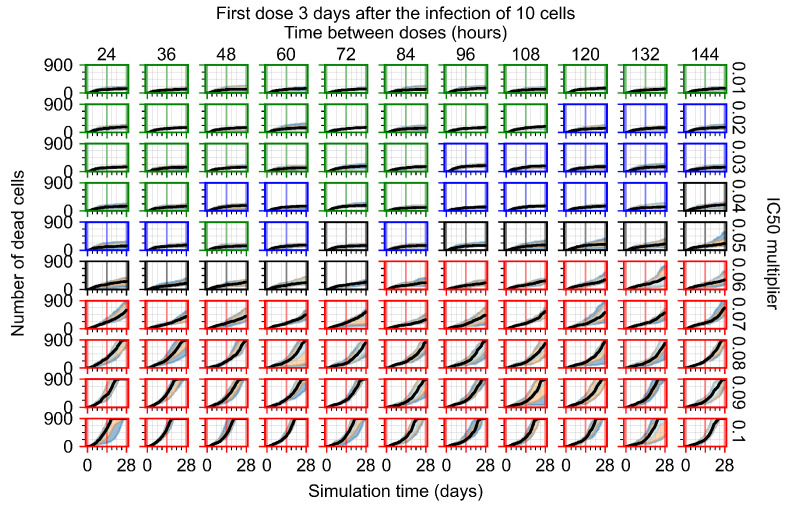
Dead population.

**Figure A113:**
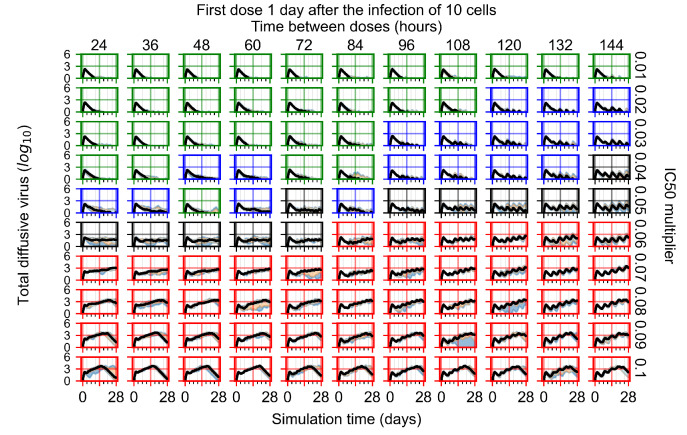
Extracellular diffusive virus quantities (arbitrary units), Y axis in log scale, and exponent values as tick marks.

**Figure A114:**
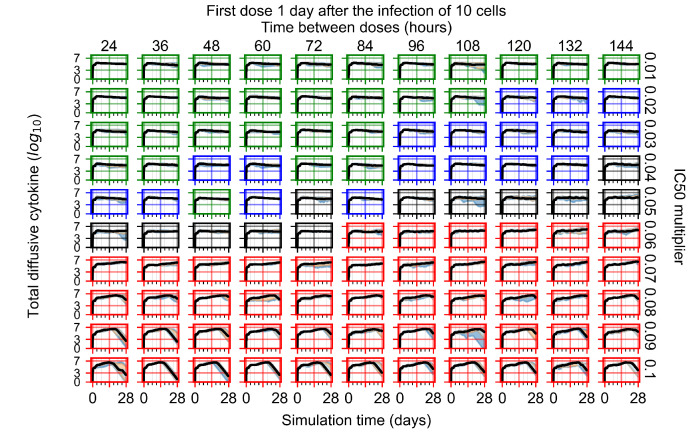
Diffusive cytokine amount for eight replicas of the treatment simulation, Y axis in log scale, and exponent values as tick marks.

**Figure A115:**
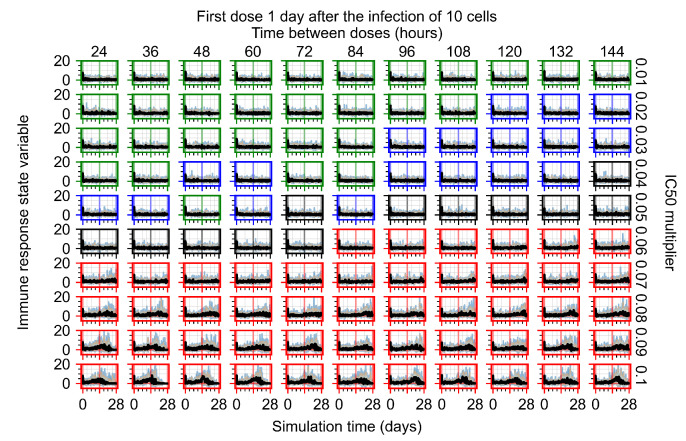
Immune response state variable number. Positive values correspond to an inflammatory state, and negative to an anti-inflammatory state.

**Figure A116:**
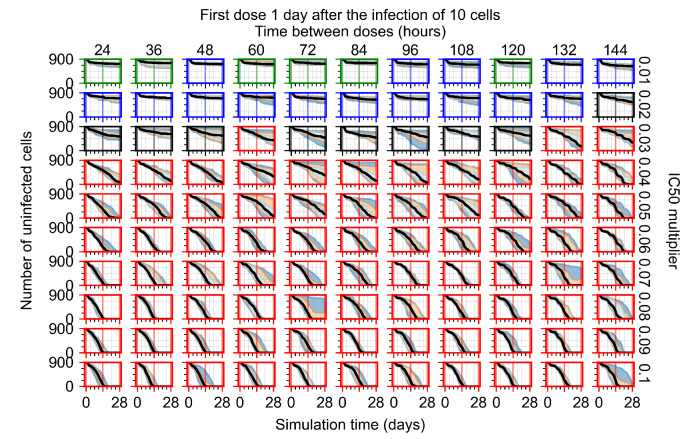
Uninfected population.

**Figure A117:**
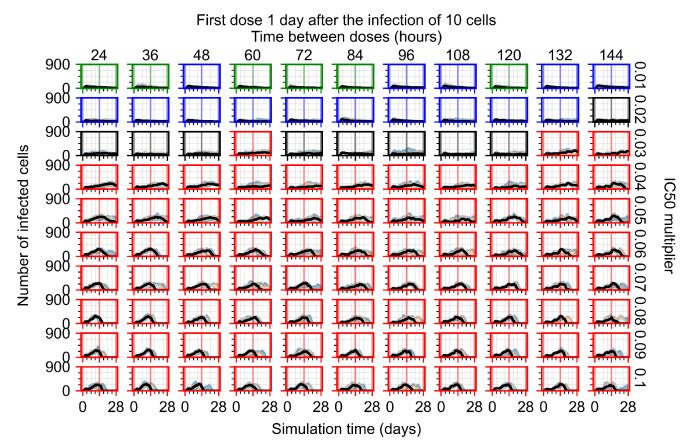
Infected (eclipse phase) population.

**Figure A118:**
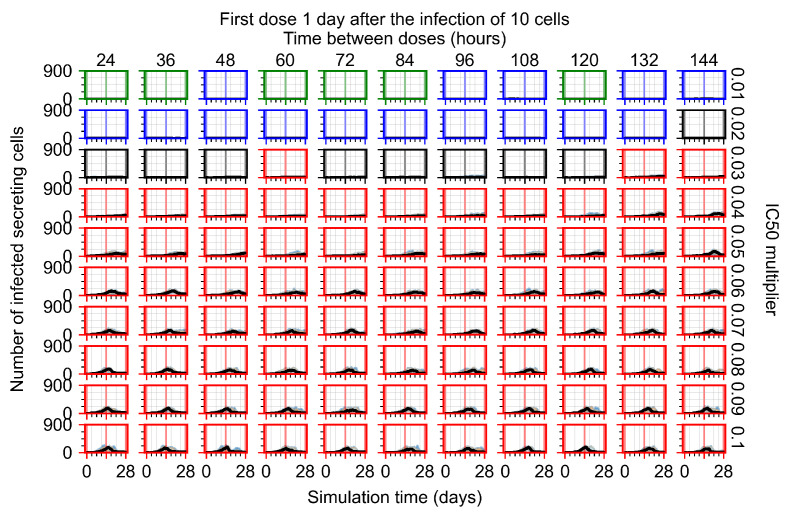
Infected (secreting extracellular virus) population.

**Figure A119:**
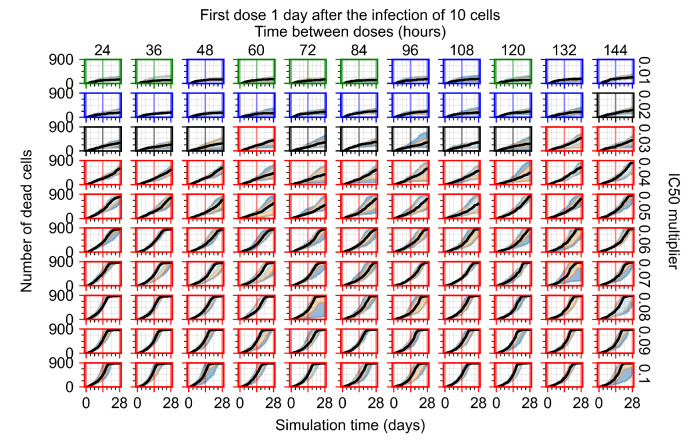
Dead population.

**Figure A120:**
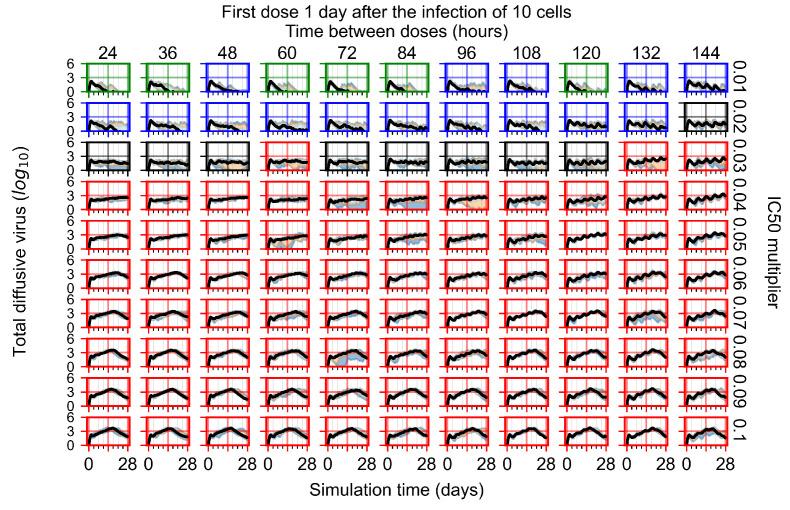
Extracellular diffusive virus quantities (arbitrary units), Y axis in log scale, and exponent values as tick marks.

**Figure A121:**
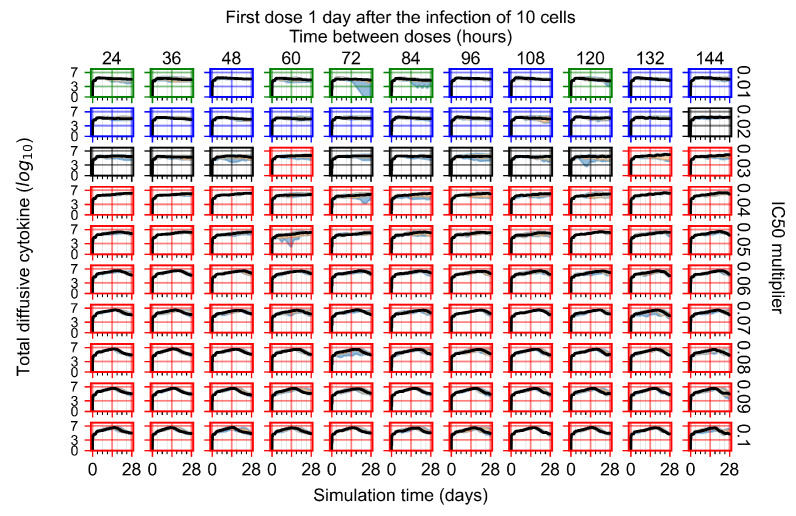
Diffusive cytokine amount for eight replicas of the treatment simulation, Y axis in log scale, and exponent values as tick marks.

**Figure A122:**
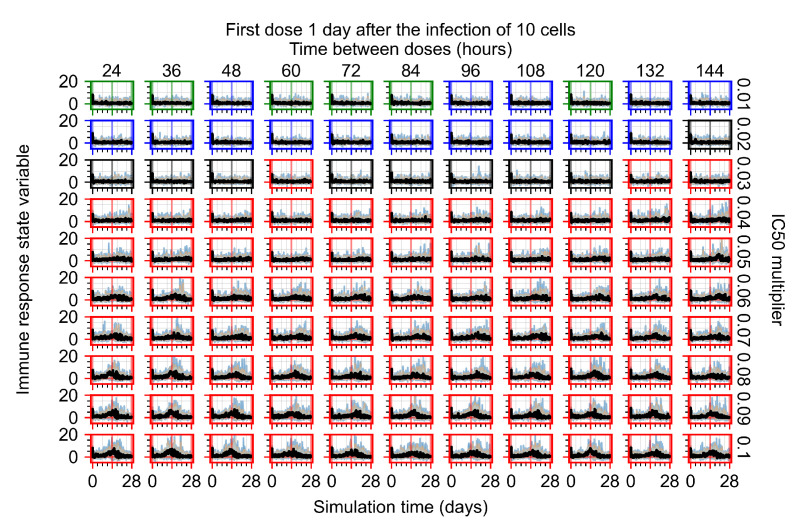
Immune response state variable number. Positive values correspond to an inflammatory state, and negative to an anti-inflammatory state.

**Figure A123:**
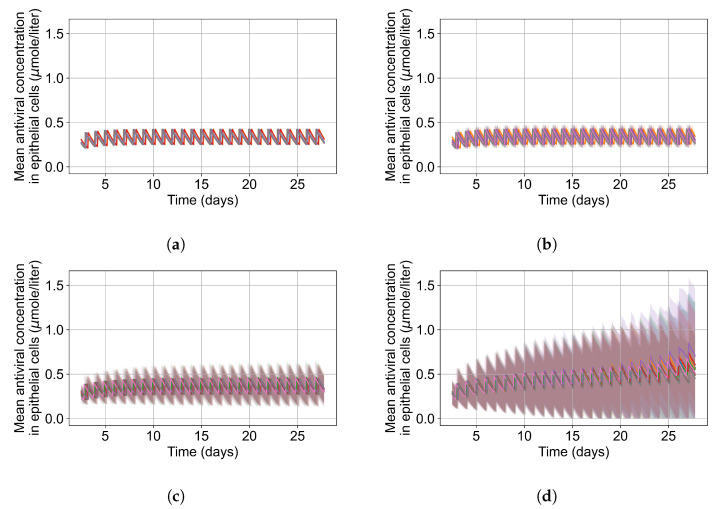
Mean antiviral drug levels in virus-releasing infected cells (solid lines) with standard deviation (shaded regions) versus time for individual simulation replicates under different simulation options. (**a**) is treated with no metabolism heterogeneity, (**b**) is treated with a metabolism standard deviation of 0.1, (**c**) is treated with a metabolism standard deviation of 0.25, and (**d**) is treated with a metabolism standard deviation of 0.25.

**Figure A124:**
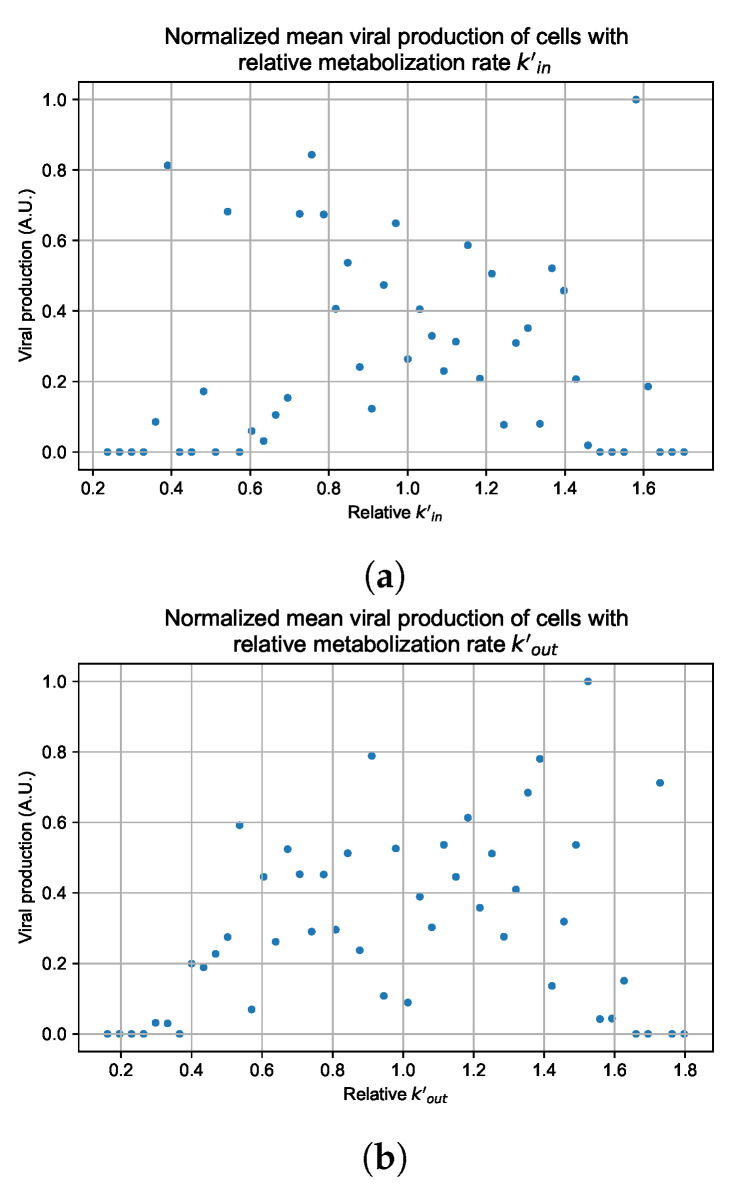
Mean viral production of cells versus their metabolism rates normalized by the maximum mean production with rapid clearance parameters. (**a**) Results for simulations varying only $k_{in}$. (**b**) Results for simulations varying only $k_{out}$.

**Figure A125:**
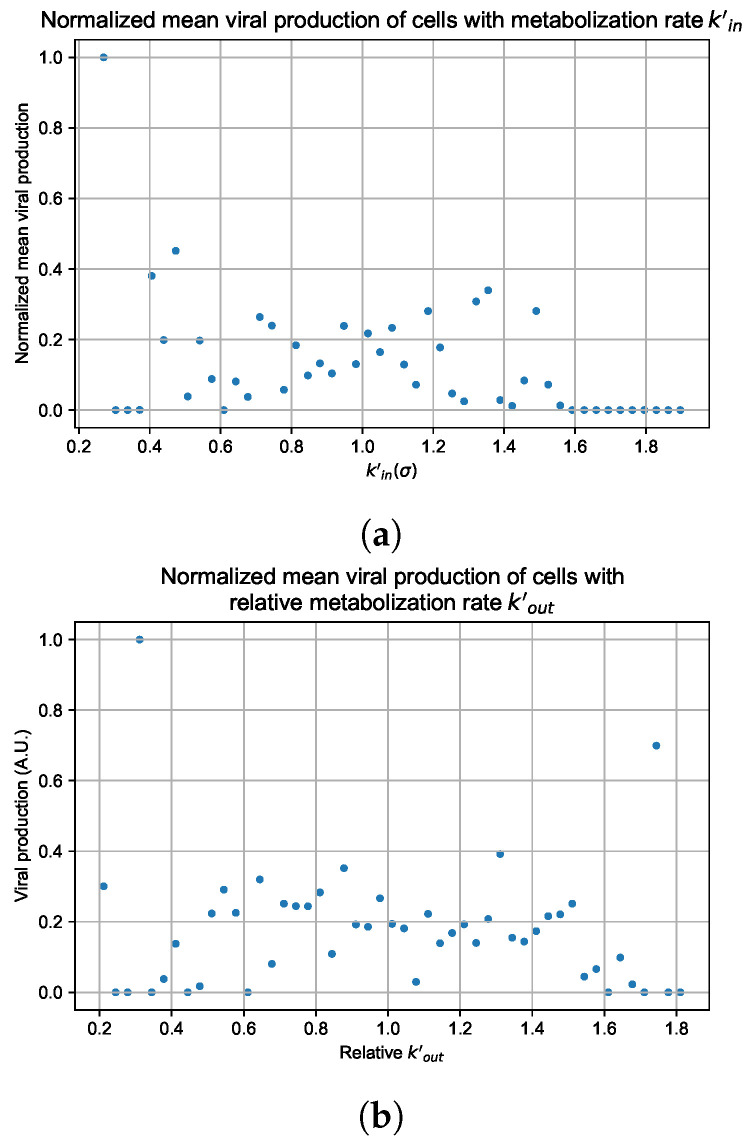
Mean viral production of cells versus their metabolism rates normalized by the maximum mean production with rapid clearance parameters. (**a**) Results for simulations varying only $k_{in}$. (**b**) Results for simulations varying only $k_{out}$.

**Figure A126:**
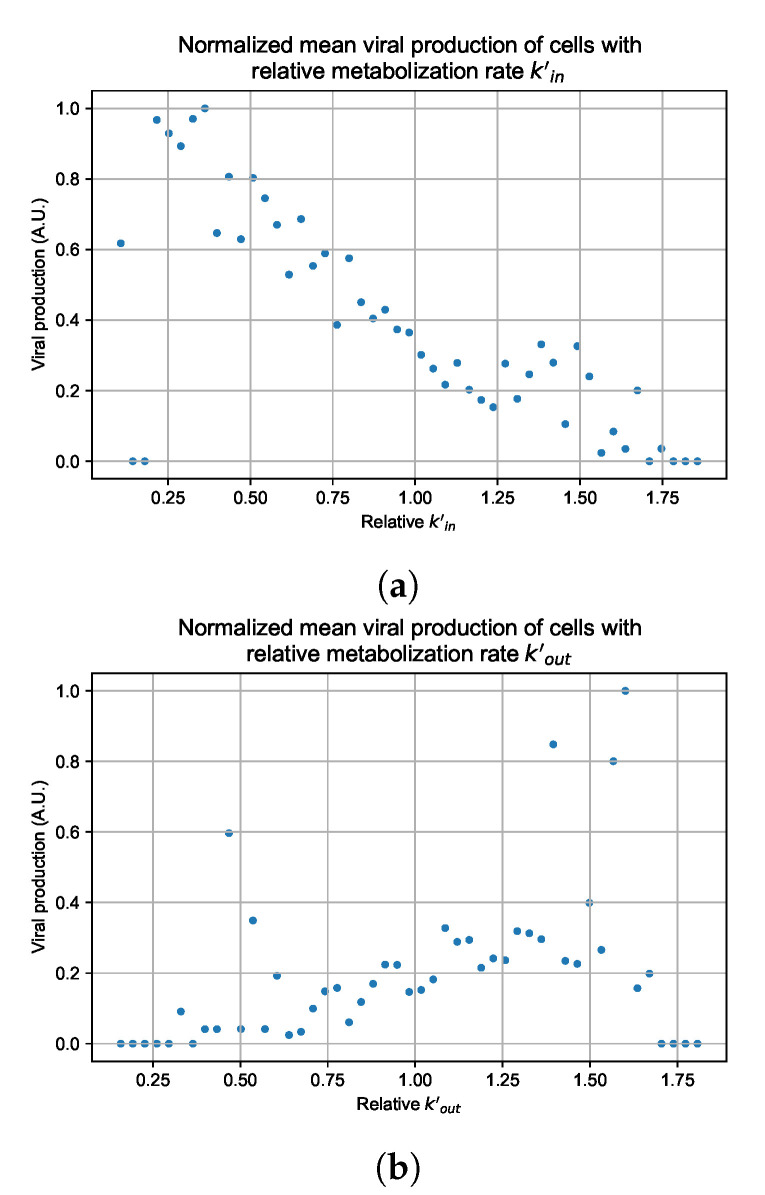
Mean viral production of cells versus their metabolism rates normalized by the maximum mean production with slow clearance parameters. (**a**) Results for simulations varying only $k_{in}$. (**b**) Results for simulations varying only $k_{out}$.

**Figure A127:**
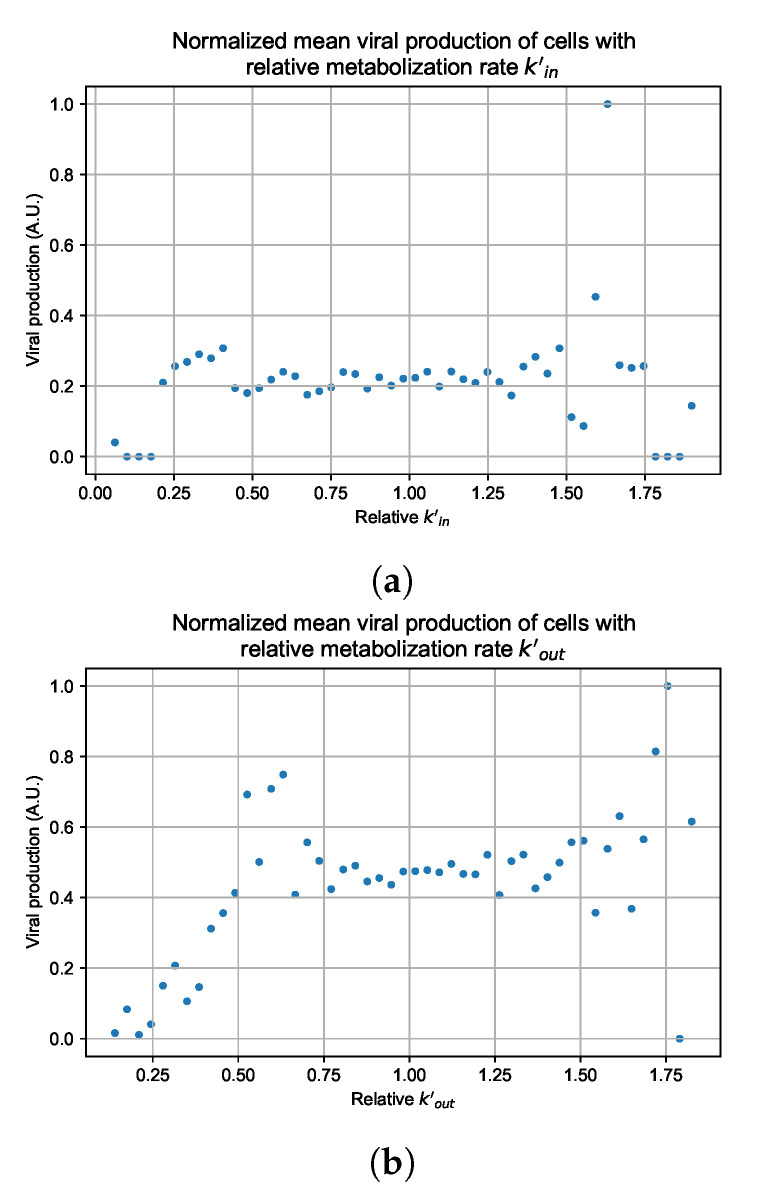
Mean viral production of cells versus their metabolism rates normalized by the maximum mean production with widespread infection parameters. (**a**) Results for simulations varying only $k_{in}$. (**b**) Results for simulations varying only $k_{out}$.

## Data Availability

The authors declare that data supporting the findings of this study are available online at https://go.iu.edu/48S7 (accessed on 3 November 2021) . COPASI and CompuCell3D are free software and can be downloaded at, respectively, http://copasi.org/ (accessed on) and www.compucell3d.org (accessed on 3 November 2021). The COPASI and CompuCell3D codes used are hosted in the gitHub repository https://github.com/JulianoGianlupi/covid-tissue-response-models/ (accessed on 3 November 2021) that can be downloaded as a https://github.com/JulianoGianlupi/covid-tissue-response-models/zipball/fossilized-repo/ (accessed on 3 November 2021) single zip file. Instructions on how to run the COPASI code are found in Appendix A. Instructions for CompuCell3D are in Appendix D.

## Abbreviations

The following abbreviations are used in this manuscript:

ABM	Agent-based model
COPASI	Complex Pathway Simulator
CPM	Cellular Potts model
EBOV	Ebola virus
GS-443902	Remdesivir Triphosphate, CAS: 1355149-45-9, CHEBI:150869
MDPI	Multidisciplinary Digital Publishing Institute
MOA	Mechanism of action
ODE	Ordinary differential equation
PBMC	Peripheral blood mononuclear cells
PBPK	Physiological based pharmacokinetic model
PD	Pharmacodynamic model
PK	Pharmacokinetic model
RdRP	RNA-dependent RNA polymerase
SBML	Systems Biology Markup Language

## Appendix A. Simple PK Model for Remdesivir and GS-443902

Gallo [18] developed a model to predict the intracellular concentration of remdesivir triphosphate (GS-443902) after IV infusion of remdesivir in humans. Their model is based on measurement of GS-443902 in peripheral blood mononuclear cells (PBMC) following IV infusion of remdesivir. That model is then used as a surrogate for predicting the concentration in lung cells assuming the exposure, uptake and metabolism of the two cell types are similar. Here, we develop a simpler model designed to just predict the GS-443902 concentration in lung cells based on the data and model of Gallo, along with data from Humeniuk et al. [7], and with additional PBMC data from the Gilead application to the European Medicines Agency’s for the compassionate use of remdesivir to treat COVID-19 patients [38]. The goal of this model is simply to estimate reasonable tissue concentrations of GS-443902 versus time as a function of remdesivir dose in repetitive dosing scenarios. These dosing scenarios are like those used by Gallo and in the compassionate use document. The model layout is shown in Figure 1 (in the main paper) and the ordinary differential equations,

(A1) $$\frac{d(C_{GS}\cdot vol)}{dt}=\left(\frac{D_{rmd}\cdot k_{in}}{\tau_{I}}\right)-vol\cdot k_{out}\cdot C_{GS}\;\;.$$

(A2) $$\frac{d(C_{GS}^{AUC})}{dt}=C_{GS}\;\;.$$

Differential equations for the minimized remdesivir PK model. Note that vol in Equations (A1) and (A2), in Figure 1 and in Table 1, is the effective volume of the single compartment in the model. Here, $D_{rmd}$ is in mols. The model’s input for remdesivir is in milligrams, which the COPASI code converts to moles (e.g., “Remdes_dose_mol”) so that conversion of remdesivir to GS-443902 is one-to-one.

Our simplified model was constructed in COPASI (http://copasi.org (accessed on 3 November 2021)), an SBML compliant biochemical system simulator well suited for modeling chemical and biological processes represented by ordinary differential equations (ODEs). COPASI allows for easy model definition, including the use of links to biological ontologies for unique identification of species and concepts, as well as a suite of tools for running simulations, parameter fitting, sensitivity analysis, etc. The COPASI file (which is setup to do the parameter estimation task) along with the parameter files based on Gallo, Humeniuk et al. and the compassionate use document are available at https://github.com/JulianoGianlupi/covid-tissue-response-models/ (accessed on 3 November 2021), https://github.com/JulianoGianlupi/covid-tissue-response-models/ (accessed on 3 November 2021), or the entire repository can be downloaded from https://github.com/JulianoGianlupi/covid-tissue-response-models/zipball/fossilized-repo/ (accessed on 3 November 2021), https://github.com/JulianoGianlupi/covid-tissue-response-models/zipball/fossilized-repo/ (accessed on 3 November 2021). Table A1 summarizes the parameters and measurements from the Humeniuk study and compassionate use documents that we used to calibrate our model.

**Table A1:** Data used to calibrate the simple remdesivir to GS-443902 prediction model. * Humeniuk’s Table 4 in [7]; ** EU Compassionate Use’s Table 16 in [38]; *** infusion duration not given, assumed to be 1 h.

Parameter	Unit	Humeniuk * Cohort 7	Humeniuk * Cohort 8	EU Compassionate Use **
Remdesivir Dose	mg	150	75	200
Infusion Duration	h	2	0.5	1.0 ***
GS-443902 $C_{max}$	uM	6.0	5.9	9.8
GS-443902 $C_{24hr}$	uM	3.7	3.3	6.9
GS-443902 $t_{1/2}$	1/h	36	49	
GS-443902 $AUC_{24h}$	h × uM			157.4
GS-443902 $AUC_{inf}$	h × uM	297	394	
GS-443902 $AUC_{last}$	h × uM	272	340	

### Appendix A.1. COPASI Codes

The COPASI model files are available on GitHub as described earlier. Simulations were developed in COPASI version 4.30 (Build 240) and have been checked through version version 4.34 (Build 251). Below, we give details for running the two models.

#### Appendix A.1.1. COPASI Model File for Parameter Fitting

The file GS-443902_PBMC_PK_v05_3data_plusEurope.cps does the parameter estimation task based on the data of [7]. The data files are included in the GitHub repository. This COPASI file is set up to do both the parameter fitting task and a basic time course simulation. Load the file in COPASI and ensure that the following data files are in the same folder that contains the COPASI .cps file:

- Humeniuk_PK_data_Europe_Table_16.txt
- Humeniuk_PK_data_Table_4_Cohort7.txt
- Humeniuk_PK_data_Table_4_Cohort8.txt

The COPASI model implements the infusion period with an event named “Infusion”, which changes the variable “$k_{in}$” from 1 to 0 when the model’s time exceeds the length of the “$\tau_{I}$” parameter (see Equation (A1) and the COPASI code). Several other events are used to determine the $T_{max}$, $C_{max}$, AUC at 24 h etc. The remdesivir dose is input in milligrams and converted to moles by the COPASI code. To run the parameter estimation, select “Task”, “Parameter Estimation”, then select an estimation method. We used the “Particle Swarm” method with the default values. In the Upper right of the COPASI window the button “Experimental Data” will open the data window. If the data files listed above are in the same directory as the .cps file then this should be populated with the data mappings for each of the three files. These values are also summarized in Table A1. In the upper right of the COPASI window, click the “update model” checkbox so the fitted parameters are updated into the starting values for the COPASI model. Click “Run” and the parameter are estimated and available on the “Results” sub-item. The Particle Swarm should iterate down to an objective value of approximately 6.2 × 10^−11^. The model has now been updated with the results for the terminal clearance half life and the effective compartment volume, typically 30.2 h and 38.4 L, respectively. A time course can be run using these parameters by selecting “Tasks”, “Time Course” then “Run”. This COPASI model generate several graphs, some of which are for the fitting task and some for the time course task.

#### Appendix A.1.2. COPASI Model File for Calculating GS-443902 from Repetitive Remdesivir Doses

The file GS-443902_PBMC_PK_v05_3data_repeatDose_Gallo.cps simulates repetitive doses of 200, then 100 × 4 mg/day. This model is based on the parameter fitting model described above. The variable $k_{in}$ controls the infusion periods as well as the infusion dose since it can be thought of as a multiplier on the infusion dose (see Equation (A1)). During the initial infusion, $k_{in}$ is 1 and in subsequent infusion periods 0.5 to implement the initial 2× loading dose. This factor in combination with the remdesivir dose (Remdes_dose_mg) value gives the 200 mg then four times 100 mg dosing pattern. The infusion timing is shown in the “$k_{in}$” plot generated by the COPASI code. Loading this model into COPASI, then “Task”, “Time Course”, “Run” produces output similar to what is shown in Figure A1. Note that the maximum units in COPASI on the Y-axis are mole/liter with values near $10^{-5}$ mol/L, which corresponds to the ∼10 μM values in Figure A1.

## Appendix B. Table of Parameters from Sego et al.

Here, we present all the parameters from Sego et al. [24]. In this work, we only changed the simulation step time length, going from 1200 s to 300 s. We have marked it with an asterix and included our value in parenthesis. The reasoning for the choice of each of the parameters can be found in their original paper in Table 1.

**Table A2:** Sego et al.’s conversion factors. We changed the simulation step time length from Sego’s simulation [24], going from 1200 s to 300 s. We have marked it with an asterix and included our value in parenthesis.

Conversion Factors	Value
Simulation step Δt	1200.0 s * (300.0 s)
Lattice width	4.0 μm
Scale factor for concentration	$10^{-14}$ mol

**Table A3:** Sego et al.’s parameters part 1.

Simulation Parameter Name	Value	Simulation Parameter Name	Value
Cell diameter	12.0 μm	Viral decay rate $\gamma_{vir}$	7.71 × 10^−6^ s^−1^
Replication rate $r_{max}$	(1/12)10^−3^ s^−1^	Cytokine diffusion coefficient $D_{cyt}$	0.16 μm^2^ s^−1^
Translating rate $r_{t}$	(1/18)10^−3^ s^−1^	Cytokine diffusion length $\lambda_{cyt}$	100 μm
Unpacking rate $r_{u}$	(1/6)10^−3^ s^−1^	Cytokine decay rate $\gamma_{cyt}$	1.32 × 10^−5^ s^−1^
Packaging rate $r_{p}$	(1/6)10^−3^ s^−1^	Maximum cytokine immune secretion rate $\sigma_{cyt}\left(immuneactivated\right)$	3.5 × 10^−4^ pMs^−1^
Release rate $r_{s}$	(1/6)10^−3^ s^−1^	Immune secretion midpoint $V_{cyt}\left(immuneactivated\right)$	1 pM
Scale factor for number of mRNA per infected cell $mRNA_{avg}$	1000 cell^−1^	Cytokine immune uptake rate $\omega_{cyt}\left(immuneactivated\right)$	3.5 × 10^−4^ pMs^−1^
Viral dissociation coefficient $r_{half}$	2000	Maximum cytokine infected cell secretion rate $\sigma_{cyt}\left(infected\right)$	3.5 × 10^−3^ pMs^−1^
Viral diffusion coefficient $D_{vir}$	0.01 μm^2^ s^−1^	Infected cell cytokine secretion mid-point $V_{cyt}\left(infected\right)$, $V_{cyt}\left(virus-releasing\right)$	0.1
Viral diffusion length $\lambda_{vir}$	36 μm	Cytokine secretion Hill coefficient $h_{cyt}$	2
Immune cell cytokine activation $EC_{50,cyt,ac}$	10 pM	Virally-induced apoptosis dissociation coefficient $V_{apo}$	100
Immune cell equilibrium bound cytokine $EQ_{ck}$	210 pM	Virally-induced apoptosis characteristic time constant $\alpha_{apo}$	20 min
Immune cell bound cytokine memory $\rho_{cyt}$	0.99998 s^−1^	Immune cell activation Hill coefficient $h_{act}$	2
Immune cell activated time	10 h	Immune response add immune cell coefficient $\beta_{add}$	1/1200 s^−1^
Oxidation Agent diffusion coefficient $D_{oxi}$	0.64 μm^2^ s^−1^	Immune response subtract immune cell coefficient $\beta_{sub}$	1/6000 cell^−1^ s^−1^
Oxidation Agent diffusion length $\lambda_{oxi}$	36 μm	Immune response delay coefficient $\beta_{delay}$	1.2 × 10^6^ s
Oxidation Agent decay rate $\gamma_{oxi}$	1.32 × 10^−5^ s^−1^	Immune response decay coefficient $\beta_{decay}$	1/12,000 s^−1^
Immune cell oxidation agent secretion rate $\sigma_{oxi}$	3.5 × 10^−3^ pMs^−1^	Immune response cytokine transmission coefficient $\alpha_{sig}$	0.5
Immune cell $C_{cyt}$ threshold for Oxidation Agent release $t_{sec}$	10 A.U. = 1.5625 pM	Immune response probability scaling coefficient $\alpha_{immune}$	0.01
Tissue cell $C_{oxi}$ threshold for death $t_{death-oxi}$	1.5 A.U. = 0.234375 pM	Number of immune cell seeding samples $n_{seeding}$	10

**Table A4:** Sego et al.’s parameters part 2.

Simulation Parameter Name	Value	Simulation Parameter Name	Value
Initial density of unbound cell surface receptors $R_{o}$	200 cell^−1^	Initial immune cell target volume	64 μm^3^
Virus–receptor association affinity $k_{on}$	1.4 × 10^4^ M^−1^ s^−1^	Immune cell lambda volume $\lambda_{volume}$	9
Virus–receptor dissociation affinity $k_{off}$	1.4 × 10^4^ s	Initial number of immune cells	0
Infection threshold	1	Immune cells lambda chemotaxis $\lambda_{chemotaxis}$	1
Uptake Hill coefficient $k_{upt}$	2	Intrinsic Random Motility $H^{M}$	10
Uptake characteristic time constant $\alpha_{upt}$	20 min	Contact coefficients J (all interfaces)	10
Virally-induced apoptosis Hill coefficient $h_{apo}$	2		

## Appendix C. Quantitative Metrics of Treatment Outcome

The classification of treatment outcomes into fast clearance, slow clearance, partial containment, or widespread infection is performed by using quantitative metrics in an algorithm. We always use the median measurements of the simulation replicas for each parameter combination. We first look at the median time course of the uninfected population, second at the median time course of the extracellular viral load, and third at the extracellular virus AUC from treatment start.

If the median uninfected population at simulation end is below ten or less than half the uninfected population at the start of treatment, we classify it as ineffective with widespread infection.

The next metric we look at is median extracellular viral load. If the viral load goes below a threshold of 1.3 A.U., the simulation has cleared the virus at least once (there may be subsequent releases of extracellular virus) but we still do not know if the infection was contained or not. If the extracellular virus was cleared in less than 14 days of treatment and the extracellular virus concentration does not rise above a slightly higher threshold of 1.1 × 1.3 A.U. = 1.43 A.U. after treatment initiation, we classify the treatment as effective with fast containment.

If the virus is cleared but then there is a rise above 1.43 A.U., we look at the maxima and minima of the logarithm of extracellular viral load post 14 days of treatment. We calculate the difference of the last maxima and the first minima (ΔM) and we compare it to another threshold of 10^−4^ A.U. If the difference is close to zero, |ΔM| < 10^−4^ A.U., we classify the treatment as ineffective with a partial containment. If the difference is less than the negative of the threshold (ΔM < −10^−4^ A.U.), we classify the treatment as effective and the clearance as slow. If the difference is above the positive threshold ($\Delta M>10^{-4}A.U.$), we classify the result as widespread infection.

If the extracellular virus level never goes below the 1.3 A.U. threshold, we first check against the 1.43 A.U. threshold; if the levels have gone below it, we classify the treatment as partial containment. If not, we look at ΔM (as before) and at the AUC from treatment initiation ($AUC_{TI}$). If $AUC_{TI}$ is below 300 A.U., we classify the treatment as ineffective with a partial containment. If not, we use ΔM and the threshold of 10^−4^ A.U. for the classification.

## Appendix D. Instructions for Running the Multiscale CompuCell3D Simulations and for Analyzing the Results

The simulation code as well as the scripts used to analyze the results are hosted in gitHub at https://github.com/JulianoGianlupi/covid-tissue-response-models/ (accessed on 3 November 2021), https://github.com/JulianoGianlupi/covid-tissue-response-models/zipball/fossilized-repo/ (accessed on 3 November 2021) download repository.

### Appendix D.1. CompuCell3D Simulations

To run the ABM, you can use either your personal computer or a cluster. You need to download CompuCell3D version 4.2.3 (or newer; investigations were perform in 4.2.3), https://compucell3d.org/SrcBin (accessed on 3 November 2021). For local installations, run the python script cellular-model/batch_run.py, and (optionally) define the output directory in the script. For cluster execution, change the output directory in cellular-model/batch_exec.py and run the script cellular-model/batch_exec.sh. The script is set up for Slurm scheduling systems. In those files, you can define the output directory (variable sweep_output_folder).

All parameters that were varied and investigated are in the file cellular-model/ investigation_dictionaries.py and are imported to batch_run.py and batch_exec.py. To change the investigated parameters, change the dictionary used as mult_dict in one of those files, e.g.,

- mult_dict = treatment_starts_0, parameters varied in the fine investigation with treatment starting with the infection of 10 epithelial cells;
- mult_dict = treatment_starts_3_halved_half_life, parameters varied in the fine investigation with treatment starting3 days post the infection of 10 epithelial cells and with the half life of GS-443902 halved.

Those files also support defining how many replicas to be executed for each parameter combinations to do with the variable num_rep. The workflow will then run through all combinations of parameters in mult_dict, generating and running “num_rep” simulations for each. All results will be stored in “sweep_output_folder”. Each parameter combination is called a “set” and all results of a set are in their respective folder, e.g., for the following parameter dictionary  the first set (set_0) uses first_dose=0, dose_interval=1, ic50_multiplier=0.01, and t_half_mult=1. The results for it will be in sweep_output_folder/set_0, each replica will be in sweep_output_folder/set_0/run_0, sweep_output_folder/ set_0/run_1, [...], sweep_output_folder/set_0/run_num_rep. The second set (set_1) then uses dose_interval=1.5 and ic50_multiplier=0.01; and so on.

### Appendix D.2. Results Analysis

There are two steps for the analysis of the ABM results. In the first step, the median behaviors of the simulation replicas are measured and used for classifying the treatment among the classes defined in the methods Section 2.6. The second step is to, then, generate the parameter variation Figures (e.g., Figure 6b).

The file cellular-model/grid_color_picker.py does step one. You only need to set the variable base_path to your path to the “set” folders. Then step two is performed by cellular-model/PostMultiSet.py, change base_path in it to be the same as in step one.

## Appendix E. Supplementary Results for the Untreated Simulations with Different Initial Conditions

Here, we have the results for the untreated simulations with different initial conditions, namely with 1, 2, 5 and 10 infected cells at the start of the simulation. All of the results for this appendix use 400 simulation replicas for each set of parameters used. For all subfigures, the median measurement of simulation replicas is the black line, the 0th to 100th quantiles are shaded as dark blue, 10th to 90th shaded in orange, and 25th to 75th as light blue.

## Appendix F. Supplementary Results from Treatment Initiation Delay, Antiviral Potency, and GS-443902 Half-Life Variation

All of the results for this appendix use eight simulation replicas for each set of parameters used. For all subfigures, the median measurement of simulation replicas is the black line, the 0th to 100th quantiles are shaded as dark blue, 10th to 90th shaded in orange, and 25th to 75th as light blue. The subplots with axis colored in green are classified as repid clearance, in blue as slow clearance, in black partial containment, and in red widespread infection. The sections names describe if the results in it are for homogeneous or heterogeneous metabolism, if the half-life of GS-443902 was altered, and how long the delay is from the infection of ten epithelial cells to treatment initiation.

### Appendix F.5. Heterogeneous Metabolism Using Other Standard Deviations

In these simulations, treatment was initialized one day after the infection of 10 cells.

#### Appendix F.5.3. How Heterogeneity Affects Intracellular Drug Levels

Here, we show how different levels of metabolism heterogeneity affect intra-cellular drug levels. For high heterogeneity, some cells have an internal concentration of antiviral close to or at zero.

## Appendix G. Supplementary Results for Viral Production Metabolism Rate Correlation

For the figures of this appendix as well as Figure 12, we performed 4 simulation replicas for each parameter set. We group the cells of all parameter set replicas into 50 bins by their metabolism rates, we then compute the mean production of that bin and the maximum viral production of all bins. We plot the mean of the bin divided by the maximum mean production of all bins versus the metabolism rate.
